# Single-cell analysis reveals a subpopulation of adipose progenitor cells that impairs glucose homeostasis

**DOI:** 10.1038/s41467-024-48914-w

**Published:** 2024-06-06

**Authors:** Hongdong Wang, Yanhua Du, Shanshan Huang, Xitai Sun, Youqiong Ye, Haixiang Sun, Xuehui Chu, Xiaodong Shan, Yue Yuan, Lei Shen, Yan Bi

**Affiliations:** 1 Department of Endocrinology, Drum Tower Hospital Affiliated to Nanjing University Medical School, Branch of National Clinical Research Centre for Metabolic Diseases, Nanjing, China; 2 Shanghai Institute of Immunology, Shanghai Jiao Tong University School of Medicine, Shanghai, China; 3 Department of General Surgery, Drum Tower Hospital Affiliated to Nanjing University Medical School, Nanjing, China

**Keywords:** Type 2 diabetes, Mechanisms of disease, Adipocytes

## Abstract

Adipose progenitor cells (APCs) are heterogeneous stromal cells and help to maintain metabolic homeostasis. However, the influence of obesity on human APC heterogeneity and the role of APC subpopulations on regulating glucose homeostasis remain unknown. Here, we find that APCs in human visceral adipose tissue contain four subsets. The composition and functionality of APCs are altered in patients with type 2 diabetes (T2D). CD9^+^CD55^low^ APCs are the subset which is significantly increased in T2D patients. Transplantation of these cells from T2D patients into adipose tissue causes glycemic disturbance. Mechanistically, CD9^+^CD55^low^ APCs promote T2D development through producing bioactive proteins to form a detrimental niche, leading to upregulation of adipocyte lipolysis. Depletion of pathogenic APCs by inducing intracellular diphtheria toxin A expression or using a hunter-killer peptide improves obesity-related glycemic disturbance. Collectively, our data provide deeper insights in human APC functionality and highlights APCs as a potential therapeutic target to combat T2D. All mice utilized in this study are male.

## Abstract (web summary)

Adipose tissue harbors functionally distinct progenitor cell subsets. Using scRNAseq and functional assays, we disclose a pathogenic adipose progenitor subset, which impairs glycose homeostasis through the construction of a detrimental niche.

## Introduction

The dramatically increasing incidence of type 2 diabetes (T2D) has become one of the largest public healthy burdens worldwide^1^. Over 60-90% of T2D patients are accompanied with obesity, with obesity-induced adipose dysfunction being a major driving force in the pathogenesis of T2D^2–5^. Mature adipocytes, immune cells and stromal cells are the major cellular components of adipose tissue. Previous studies have indicated that adipocytes and immune cells participate in the regulation of metabolic homeostasis and are dysregulated in obesity and T2D. However, the effect of stromal cells on maintaining of adipose tissue homeostasis remains largely unknown.

Adipose progenitor cells (APCs) represent a prominent stomal cellular component of adipose tissue and are now identified as highly heterogenous populations^6–10^. In a steady state, APCs orchestrate adipose homeostasis by interacting with immune cells, sympathetic neurons, and adipocytes^11,12^. In obesity, APCs are dramatically expanded in both mice and humans and contribute to the development of adipose tissue fibrosis and inflammation^8–10,13–15^, suggesting their potential role in impairing glucose homeostasis. However, the influence of obesity on human APC heterogeneity and the role of these APC subpopulations in regulating T2D development remain unknown.

In the current study, using a cohort of lean individuals, participants with obesity and T2D patients with obesity, we uncovered the alterations in human APC composition and functionality and identified an APC subpopulation contributing to T2D development. Notably, these pathogenic APCs secreted abundant bioactive proteins and form a detrimental niche, impairing the function of non-immune cell components. We further highlighted that depleting pathogenic APCs in mice, either genetically or pharmacologically, improved obesity-related glycemic disturbances. These findings advance current insights into human APC functionality and highlight APCs as potential therapeutic targets for combating T2D.

## Results

### ScRNA-seq identifies four subsets of human visceral adipose tissue APCs

**Fig. 1:**
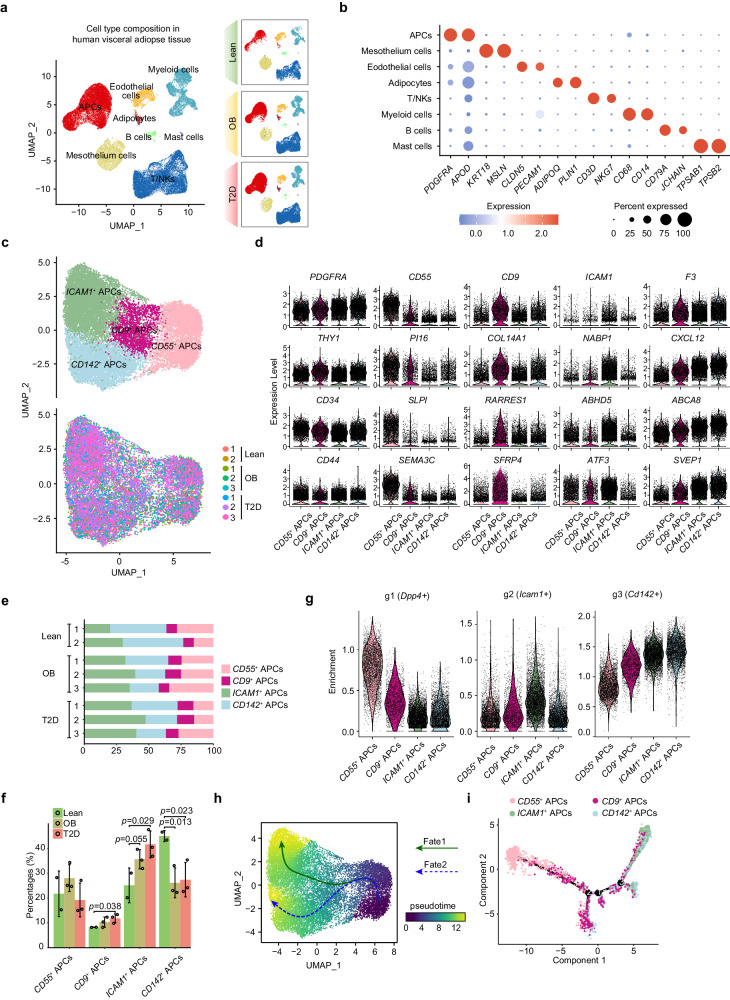
ScRNA-seq reveals the APC heterogeneity in human visceral adipose tissue. **a** Uniform manifold approximation and projection (UMAP) analyses of transcriptional profiles of human adipose stromal vascular fraction (SVF) (*n* = 55705 cells). Each cluster was shown in different color. Lean, lean individuals; OB, participants with obesity; T2D, patients with type 2 diabetes and obesity. **b** Dot plots showing average expression of known marker genes in indicated cell clusters. The dot size represents percent of cells expression the genes in each cluster. The expression intensity of markers is shown. **c** Unsupervised clustering of 18606 APCs reveals four distinct subpopulations. Lean, lean individuals; OB, participants with obesity; T2D, patients with type 2 diabetes and obesity. **d** Violin plots showing the expression levels and distribution of representative marker genes. **e** Proportion of four APC subpopulations showing in bar plots in different donors. Lean, lean individuals; OB, participants with obesity; T2D, patients with type 2 diabetes and obesity. **f** Proportion of four APC subpopulations in three groups. Lean, lean individuals; OB, participants with obesity; T2D, patients with type 2 diabetes and obesity. **g** Violin plots showing the enrichment score of gene signatures of APCs subpopulation reported by Merrick et al. in our APCs subsets. *Dpp4*^+^ subset indicates multipotential progenitors. *Icam1*^+^ subset indicates committed progenitors. *Cd142*^+^ subset indicates adipogenesis-regulatory progenitors. The scores were calculated by the average expression of each gene signature. **h**, **i** Cell trajectory analysis of APCs by Monocle3 (**h**) and Monocle2 (**i**). Data are means ± SD. Data in (**f**) were analyzed using Wilcoxon rank-sum test. Source data are provided as a Source data file.

To elucidate the compositional alteration of adipose stromal-vascular fraction (SVF) in the development of T2D, omental adipose tissue was surgically obtained from lean control participants (*n* = 2), participants with obesity (*n* = 3) and newly diagnosed T2D patients with obesity (*n* = 3), whose clinical characteristics are summarized in Supplementary Table 1. Fresh specimens were processed to delineate the human adipose SVF atlas using 10× Genomics scRNA-seq (Supplementary Fig. 1a). After filtering the scRNA-seq data to exclude dead cells, putative cell doublets, erythrocytes, and neutrophils, 55705 cell transcriptomes were obtained for subsequent analysis (11248 from lean individuals; 20381 from participants with obesity; 24076 from T2D patients with obesity). Using graph-based clustering to distinguish the dataset, eight canonical cell clusters were identified and visualized, with each cell population containing cells from the lean individuals, participants with obesity and T2D patients with obesity (Fig. 1a). The cell populations were annotated based on the expression of canonical marker genes (Fig. 1b, and Supplementary Fig. 1b). Expression of *PDGFRA* and *APOD*, the main markers of progenitor cells, was restricted to the APCs (*n* = 18606). Mast cells (*n* = 184) were defined based on *TPSAB1* and *TPSB2* expression. Endothelial cells (*n* = 3104) were distinguished using *CLDN5* and *PECAM1*. B cells (*n* = 741) expressed classical markers *CD79A* and *JCHAIN*. Expression of *ADIPOQ* and *PLIN1* defined adipocytes (*n* = 515) that may be newly differentiated. Mesothelial cells (*n* = 6370) were positive for *KRT18* and *MSLN*. Myeloid cells (*n* = 8104) were marked by *CD68* and *CD14*, and T/natural killer (NK) cells (*n* = 18081) expressed the T-cell receptor signaling mediators *CD3D* and *NKG7*, which contain various immune cell types (Supplementary Fig. 1c, d). Although eight major cell types were present in all the three groups, the percentage of each cell type was different. As shown in Supplementary Fig. 1e, APC, T/NK cells, and myeloid cells comprised the major cell fractions, of which APC constituted the largest proportion of SVF. Meanwhile, compared with the lean control group, the frequency of APC was significantly increased in the T2D group; in contrast, the frequency of mesothelium cells was decreased, while litter changes were observed in other cell types (Supplementary Fig. 1f).

We further investigated the composition and diversity of 18606 APCs from all samples. The UMAP analysis identified four distinct clusters, each containing cells from all donors, suggesting that there was no donor-specific transcriptomic profile (Fig. 1c, Supplementary Fig. 2a). The *CD9*^*+*^ APCs were enriched in the expression of *CD9*, *COL14A1*, *RARRES1* and *SFRP4* (Fig. 1d, Supplementary Fig. 2b). The expression of *CD55*, *PI16*, *SLPI*, and *SEMA3C* distinguished *CD55*^*+*^ APCs from the other APC clusters. *ICAM1*^*+*^ APCs were characterized by the expression of *ICAM1*, *NABP1*, *ABHD5*, and *ATF3*. The remaining APC cluster was distinguished by relatively high expression levels of *F3*, *CXCL12*, *ABCA8* and *SVEP1*. Compared with lean controls, the percentages of *CD9*^*+*^ APCs and *ICAM1*^*+*^ APCs appeared to be higher in participants with obesity and T2D patients with obesity, whilst *CD142*^*+*^ APCs tended to be lower (Fig. 1e, f). Gene ontology (GO) analysis indicated that *CD55*^*+*^ APCs, *CD9*^*+*^ APCs, and *CD142*^*+*^ APCs were highly enriched in genes associated with the extracellular structure organization pathway (Supplementary Fig. 2c). Both *CD9*^*+*^ APCs and *CD55*^*+*^ APCs were enriched in the response to TGFβ stimulation. *ICAM1*^*+*^ APCs were enriched in many biological process terms, such as response to mechanical stimulus, fibroblast growth factor, and calcium ions. The ontology term for angiogenesis regulation was shown to be related to *CD142*^*+*^ APCs (Supplementary Fig. 2c).

To better place our APC data into the overall context of the published literature, we integrate our single-cell data with those reported by recent studies^6,7,16^ (Supplementary Fig. 2d). Four APC subpopulations identified in our study could be finely distinguished in the integrated dataset. *Icam1*^+^ committed progenitor and *Dpp4*^*+*^ multipotential progenitor identified by individual studies were generally preserved after integration. Next, we mapped our dataset to previously identified APC subpopulations (Fig. 1g, and Supplementary Fig. 2e, f). *CD55*^*+*^ APCs strongly resembled previously identified multipotent progenitors. *ICAM1*^*+*^ APCs expressed a gene signature similar to that of committed preadipocytes, whereas *CD142*^*+*^ APCs tended to be adipogenesis-regulatory cells. Notably, *CD9*^*+*^ APCs did not correspond to any of these subpopulations, indicating the presence of a previously undefined subpopulation. To assess the intrinsic relationship between the four progenitor clusters, we performed in silico pseudotemporal trajectory analysis. *CD55*^*+*^ APCs which correspond to multipotent progenitors were selected as the root state. We observed a differentiation trajectory from *CD55*^*+*^ APCs to *CD9*^*+*^ APCs, which further went to *ICAM1*^*+*^ APCs or *CD142*^*+*^ APCs, respectively (Fig. 1h). Another trajectory analysis inferred that *CD55*^*+*^ APCs can transition through the first and second branch points to become *CD9*^*+*^ APCs. Some *CD55*^*+*^ APCs continue transit through the third branch point to form the cluster of *ICAM1*^*+*^ or *CD142*^*+*^ APCs (Fig. 1i). In summary, human visceral APCs form heterogeneous populations comprising four subsets: *CD55*^*+*^ APCs, *CD9*^*+*^ APCs, *ICAM1*^*+*^ APCs, and *CD142*^*+*^ APCs.

### CD9^+^CD55^low^ APCs are associated with T2D development

**Fig. 2:**
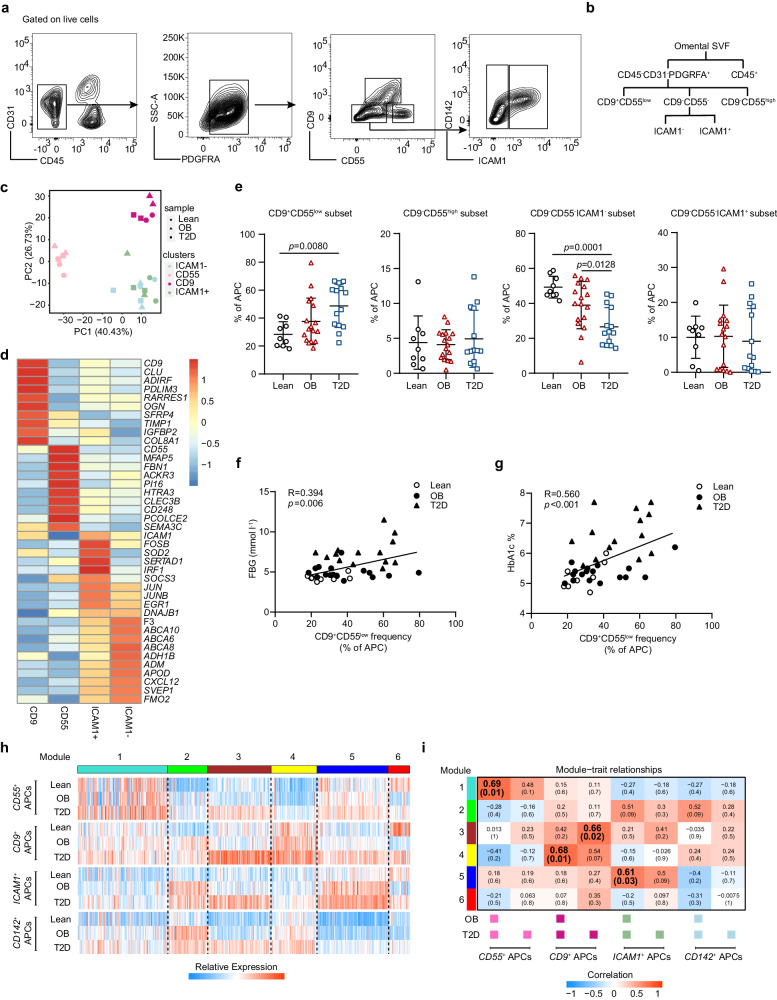
CD9^+^CD55^low^ APCs correlate with the development of T2D. **a** Gating strategy of four APC subpopulations by flow cytometry. **b**–**d** Four APC subpopulations were sorted and performed bulk RNA-seq. **b** Schematic of FACS-based selection of four APC subpopulations. **c** PCA score plot. Each APC cluster was shown in different color. Lean, lean individuals; OB, participants with obesity; T2D, patients with type 2 diabetes and obesity. **d** A heatmap of expression of marker genes defining the clusters indicated in (**c**). **e** Frequency of each APC subpopulation in SVF of lean control subjects (*n* = 9), patients with obesity (*n* = 17), and T2D patients with obesity (*n* = 14). Lean, lean individuals; OB, participants with obesity; T2D, patients with type 2 diabetes and obesity. **f**, **g** Correlation between fasting blood glucose and HbA1c levels with frequency of CD9^+^CD55^low^ APCs (*n* = 40). FBG, fasting blood glucose. Lean, lean individuals; OB, participants with obesity; T2D, patients with type 2 diabetes and obesity. **h** A heatmap showing the expression patterns of each module of genes generated by WGNCA. Lean, lean individuals; OB, participants with obesity; T2D, patients with type 2 diabetes and obesity. **i** A heatmap of Pearson correlation analysis between the gene modules and the four APCs subsets under obesity or T2D status. Color of the cells in heatmap represents the correlation coefficient. OB, participants with obesity; T2D, patients with type 2 diabetes and obesity. Data are means ± SD. Data in **e** were analyzed using one-way ANOVA followed by Tukey’s multiple comparison test; for **f**, **g**, Spearman’s bivariate correlation test (One-tailed) were used. Source data are provided as a Source data file.

To validate the existence of the four APC clusters identified by scRNA-seq, unique marker genes distinguishing these four clusters were identified by screening for genes encoding cell surface proteins (Supplementary Fig. 3a). Flow cytometry corroborated the findings of our scRNA-seq analysis and demonstrated that CD9, CD55, and ICAM1 could clearly distinguish the four APC subpopulations (Fig. 2a,b, Supplementary Fig. 3b). To further elucidate the molecular identities of these progenitor clusters, individual APC subpopulations were sorted using fluorescence-activated cell sorting (FACS) to perform bulk RNA-seq. Principal component analysis (PCA) showed that the first principal component distinguished CD9^−^CD55^high^ APCs, while the second principal component separated CD9^+^CD55^low^ APCs from the others (Fig. 2c). The transcriptional profiles of sorted cells were mapped using our scRNA-seq dataset. Remarkably, the transcription signatures of the four clusters from the bulk RNA-seq were consistent with those from the scRNA-seq (Fig. 2d). For instance, CD9^−^CD55^high^ APCs expressed pronounced levels of marker genes of *CD55*^*+*^ APCs, such as *CD55* and *PI16*. *CD9* and *RARRES1* were enriched in CD9^+^CD55^low^ APCs (Fig. 2d).

To investigate APC alterations in T2D, another cohort, including lean controls, participants with obesity, and T2D patients with obesity, was enrolled. Compared to the lean control group, the frequency of CD9^+^CD55^low^ APCs was significantly higher in T2D patients with obesity (Fig. 2e). In contrast, compared to controls, the frequency of CD9^−^CD55^-^ICAM1^-^ APCs was decreased in participants with obesity, which was further decreased in T2D patients with obesity (Fig. 2e). The frequencies of the remaining APCs did not differ significantly among the three groups (Fig. 2e). Interestingly, the frequency of CD9^+^CD55^low^ APCs positively correlated with fasting blood glucose and HbA1c levels (Fig. 2f, g). To investigate whether and how APCs contribute to the pathogenesis of obesity and T2D, we performed WGCNA to elucidate the functional alterations in APCs under disease conditions. Compared with lean controls, the up-regulated genes in participants with obesity and T2D patients with obesity were identified and divided into six functional modules (Fig. 2h). Among these functional modules, the genes in T2D patients were mainly enriched in modules 3, 4, and 5, which were preferentially prominent in the *CD9*^*+*^ APCs (Fig. 2h). The increased expression of many module genes in CD9^+^CD55^low^ APCs from T2D patients was further confirmed by qPCR assay, compared to that in lean controls (Supplementary Fig. 3c, d). Additionally, module-trait relationship analysis revealed that modules 3 and 4 positively correlated with *CD9*^*+*^ APCs, whereas module 5 was related to *ICAM1*^*+*^ APCs (Fig. 2i). Subsequent GO analysis showed that, module 3 exhibited ontological terms consistent with cell chemotaxis and stress responses (Supplementary Fig. 3e). Module 4 contained multiple genes related to extracellular matrix remodeling and cell adhesion, whereas module 5 displayed ontology terms related to responses to external stimuli (Supplementary Fig. 3e). Taken together, these data indicate that CD9^+^CD55^low^ APCs are associated with the development of T2D, characterized by marked expansion and functional reprogramming.

### CD9^+^CD55^low^ APCs promote glycemic disturbance in vivo

**Fig. 3:**
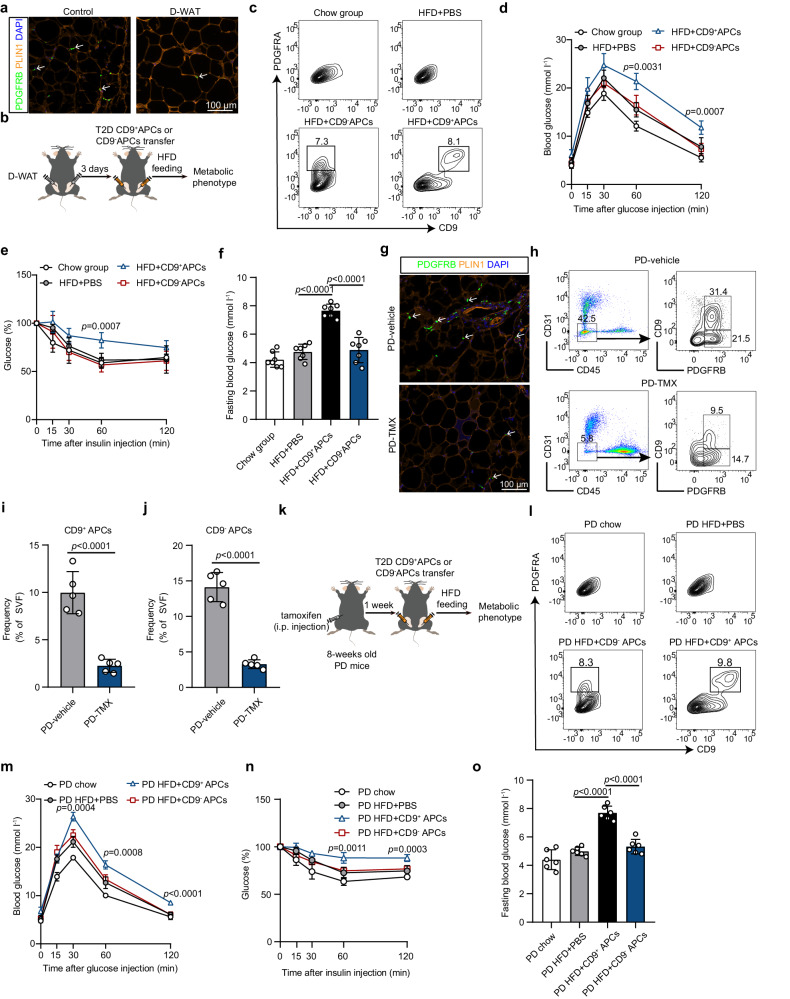
CD9^+^CD55^low^ APCs contribute to the development of T2D. **a** Representative images showing APC staining in eWAT of D-WAT treated and control mice. White arrows indicate typical stained cells. For **b**–**f**, Three days after D-WAT treatment in C57BL/6 mice, about 1×10^6^ CD9^+^CD55^low^ APCs or CD9^-^ APCs isolated from T2D patients with obesity were transplanted into APC-depleted mice, which were fed a HFD for two months (*n* = 10 per group). **b** Schematic of cell transplantation experiment. **c** One week later, flow cytometry was conducted to detect transplanted APCs by staining with anti-human PDGFRA and anti-human CD9 antibodies. Representative plots indicate detected human APCs in recipient mice. One month later, glucose tolerance test (**d**) and insulin tolerance test (**e**) were performed (*n* = 5 per group). **f** Two month later, fasting blood glucose levels were measured (*n* = 7 per group). For **g**–**j**, eight-weeks old PD mice were injected with tamoxifen or vehicle for 5 days (*n* = 5 per group). One week later, remaining APCs were detected by immunofluorescence and flow cytometry. **g** Representative images showing APC staining. White arrows indicate typical stained cells. **h** Representative plots indicate CD9^+^ and CD9^-^ APCs in eWAT of tamoxifen treated PD mice. **i**, **j** Frequencies of CD9^+^ and CD9^-^ APCs in eWAT of tamoxifen treated PD mice (*n* = 5 per group). For **k**–**o**, One week after tamoxifen injection in eight-weeks old PD mice, about 1×10^6^ CD9^+^CD55^low^ APCs or CD9^-^ APCs from T2D patients were transplanted into APC-depleted mice, which were subsequently challenged with HFD for two months (*n* = 9 per group). **k** Schematic of cell transplantation experiment. **l** One week after cell transplantation, flow cytometry was conducted to detect transplanted APCs by staining with anti-human PDGFRA and anti-human CD9 antibodies. Representative plots indicate transferred APCs in recipient mice. One month later, glucose tolerance test (**m**) and insulin tolerance test (**n**) were performed (*n* = 5 per group). **o** Two month later, fasting blood glucose levels were measured (*n* = 6 per group). Data are means ± SD. For **d**–**f** and **m**–**o**, One-way ANOVA followed by Tukey’s multiple comparison test was used; for **i**, **j**, Two-tailed unpaired Student’s *t* test was used. Source data are provided as a Source data file.

To determine whether CD9^+^CD55^low^ APCs contribute to the development of T2D, CD9^+^CD55^low^ APCs and CD9^−^ APCs from T2D patients were adoptively transferred to mice, which were then fed a high-fat diet (HFD) to induce obesity. To minimize the influence of resident mice APCs, the peptide D-WAT, which has been shown to selectively eliminate progenitor cell^17,18^, was used to deplete APCs in the epididymal adipose tissue (eWAT) of recipient mice via in situ injection. As illustrated by immunostaining, APCs were efficiently depleted in the eWAT of the D-WAT-treated mice (Fig. 3a). Flow cytometry analysis further confirmed that the frequencies of both CD9^+^ APCs and CD9^−^ APCs in the eWAT of the D-WAT-treated mice were decreased (Supplementary Fig. 4a–c). Next, CD9^+^CD55^low^ APCs and CD9^−^ APCs were isolated from T2D patients with obesity and transplanted into the eWAT of APC-depleted mice, which were fed a HFD for two months (Fig. 3b). One week after cell transplantation, both the CD9^+^ and CD9^−^ transplanted APCs were detected in the eWAT of the recipient mice (Fig. 3c). Transplanting CD9^+^ APCs led to impaired glucose intolerance and insulin sensitivity compared to the mice receiving CD9^−^APC transplantation (Fig. 3d, e). Two months after cell transplantation, compared to mice transplanted with CD9^−^ APCs, fasting blood glucose levels were notably elevated in the mice that received CD9^+^APC transplantation (Fig. 3f). Of note, transplanting CD9^−^ APCs had no effect as compared to injecting PBS.

To further ascertain the deleterious effects of CD9^+^CD55^low^ APCs on glucose homeostasis, we generated *Pdgfra*-CreERT2;DTA^flox/−^ (PD) mice by crossing *Pdgfra*-CreERT2 mice with Rosa26-LSL-DTA mice, which allowed us to genetically deplete APC through tamoxifen-induced DTA expression. As expected, immunostaining showed that the eWAT of tamoxifen-treated PD mice contained fewer APCs than the eWAT of untreated mice (Fig. 3g). In accordance with the histological data, flow cytometry analysis confirmed that the frequencies of both CD9^+^ APCs and CD9^−^ APCs in eWAT from the tamoxifen-treated mice dramatically decreased compared to that in the untreated mice (Fig. 3h–j). Intriguingly, APC depletion did not affect metabolic parameters in the PD mice, such as body weight, food intake, adipocyte morphology, or glucose tolerance (Supplementary Fig. 4d–k). Using this mouse model, CD9^+^CD55^low^ APCs and CD9^−^ APCs from diabetic patients were sorted and transplanted in situ into the eWAT of APC-depleted PD mice, which were subsequently challenged with HFD for two months (Fig. 3k). One week after cell transplantation, transplanted CD9^+^ APCs and CD9^−^ APCs were detected in the eWAT of the recipient mice (Fig. 3l). Consistently, CD9^+^APC transplantation resulted in more severe impairment on glucose tolerance and insulin sensitivity in the recipient mice compared to the mice transplanted with CD9^−^ APCs (Fig. 3m, n). Two months thereafter, higher fasting blood glucose levels were observed in the recipient mice transplanted with CD9^+^ APCs compared to CD9^−^APC transplanted mice (Fig. 3o). Meanwhile, transplanting CD9^−^ APCs had no effect as compared to injecting PBS. Collectively, these in vivo data demonstrated the crucial role of CD9^+^CD55^low^ APCs in driving T2D progression.

### Secretome of CD9^+^CD55^low^ APCs is dramatically altered in diabetes

**Fig. 4:**
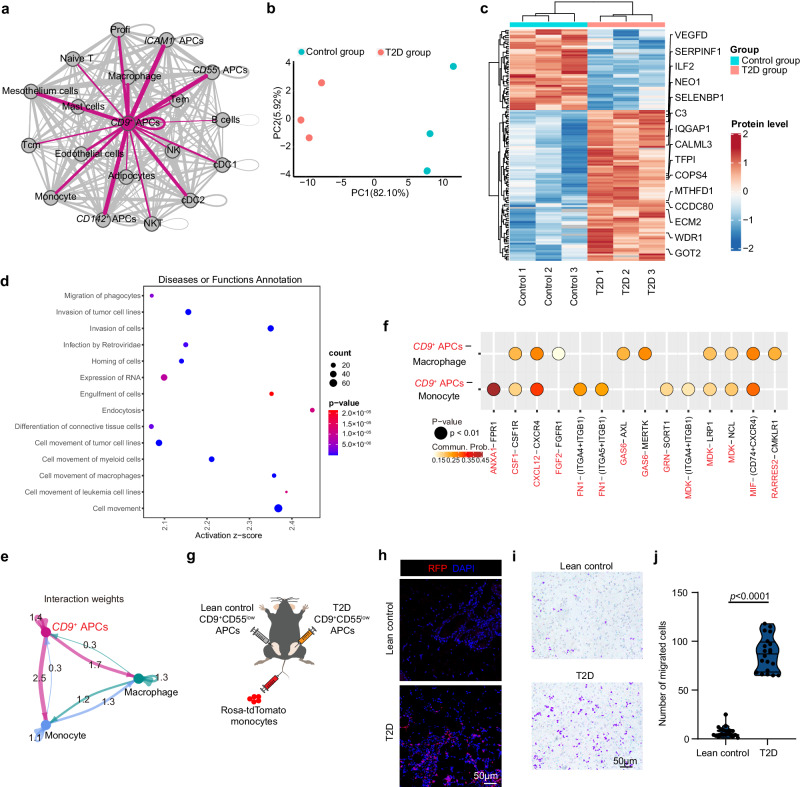
Secretome of CD9^+^CD55^low^ APCs is dramatically altered in diabetes. **a** The interaction network established by Cellphone DB between APCs clusters and other major cell types; size and number of lines represents the interaction counts. **b**–**d** CD9^+^CD55^low^ APCs from lean control subjects and T2D patients with obesity were isolated and cultured in serum free PAM for 48 hours. Conditioned mediums were collected to analyze differential secreted proteins by DIA-MS. **b** PCA score plot. **c** A heatmap showing the differential secreted proteins in T2D patients with obesity. **d** IPA analysis of increased secreted proteins by CD9^+^CD55^low^ APCs from T2D patients with obesity. **e** The interaction weights of ligand-receptor pairs calculated by Cellphone DB between *CD9*^*+*^ APCs, macrophages and monocytes. **f** Representative ligand-receptor pairs involved in the interaction between *CD9*^*+*^ APCs and macrophages or monocytes. **g**–**j** Approximately 1×10^6^ CD9^+^CD55^low^ APCs from lean control subjects and T2D patients with obesity were isolated by flow cytometry and transferred into the unilateral eWAT of C57BL/6 mice. Approximately 5×10^5^ monocytes from the blood of 10-week-old Rosa26-tdTomato mice were freshly isolated using flow cytometry and injected into mice receiving APC transfer via the tail vein. Two days later, the Tomato^+^ cells in the eWAT of recipient mice were analyzed by immunofluorescence. **g** The experiment strategy of the in vivo monocyte infiltration assay. **h** Representative staining of infiltrated monocytes in eWAT of recipient mice. Data are representative of three independent experiments. **i**, **j** Human blood monocytes were treated with conditioned mediums of CD9^+^CD55^low^ APCs from lean control subjects or T2D patients with obesity. Twenty-four hours later, migrated monocytes were stained and quantified. **i** Representative images of migrated monocytes. **j** Quantification of migrated cells. Data are representative of three independent experiments. Data are means ± SD. Data in **e** were analyzed using Right-Tailed Fisher’s Exact Test. Data in **f** were analyzed using permutation test. Data in (**j**) were analyzed using Two-tailed unpaired Student’s *t* test. Source data are provided as a Source data file.

Based on our scRNA-seq dataset, we attempted to elucidate the pathogenic mechanisms through which CD9^+^CD55^low^ APCs cause metabolic dysregulation. We first utilized the cellphone DB, an unbiased ligand-receptor interaction analysis, to reveal the cellular interactome in human adipose tissue. As illustrated in Fig. 4a, *CD9*^*+*^ APCs exhibited extensive interactions with other cell components. To perform a detailed analysis, we conducted data-independent acquisition mass spectrometry (DIA-MS) to delineate the secretome of CD9^+^CD55^low^ APCs from lean controls and T2D patients with obesity. A total of 684 proteins were captured across all samples, with little variance between samples. PCA revealed that the secretome of CD9^+^CD55^low^ APCs was notably altered in T2D patients (Fig. 4b). Among the detected proteins, 84 were upregulated, and 45 were downregulated in the T2D group (Fig. 4c). These proteins secreted by CD9^+^CD55^low^ APCs from T2D patients can be divided into several broad categories based on their known biological functions, including chemokines, growth factors, and catalytic enzymes. Accordingly, ingenuity pathway analysis (IPA) showed that multiple pathways were enriched, characterized by those responsible for cell migration as well as the regulation of angiogenesis and protein synthesis (Fig. 4d).

Consistent with the high chemotaxis activity revealed by IPA, CellPhone DB analysis also showed numerous interactions between ligands and cognate receptors expressed by the *CD9*^*+*^ APCs and macrophages, among which multiple chemokine-receptor interactions were observed (Fig. 4e, f). To confirm the chemotactic capacity of CD9^+^CD55^low^ APCs, in vivo migration assay was conducted. CD9^+^CD55^low^ APCs from lean control individuals and T2D patients with obesity were transplanted in situ into the unilateral eWAT of C57BL/6 mice, after which exogenous tomato-red monocytes from Rosa26-tdTomato transgenic mice were intravenously injected (Fig. 4g). Increased infiltration of exogenous monocytes was found in the eWAT of mice receiving diabetic CD9^+^CD55^low^ APCs transplantation compared to in that of mice receiving control CD9^+^CD55^low^ APCs transplantation (Fig. 4h). Functional assays further demonstrated that the conditioned medium of CD9^+^CD55^low^ APCs cultured from T2D patients promoted the migration of circulating monocytes compared to that cultured from lean controls (Fig. 4I, j). After comparing the DIA-MS data with the CellPhone DB analysis, four proteins (MDK, APP, RARRES2, and GRN) were further evaluated, which showed corresponding receptor expression in macrophages (Supplementary Fig. 5a). For validation, sorted monocytes from blood of T2D patients were exposed to culture media supplemented with recombinant human MDK, APP, and GRN proteins. Among these proteins, recombinant MDK significantly increased the number of migrated monocytes, whereas this effect was not observed for the other proteins (Supplementary Fig. 5b, c).

### CD9^+^CD55^low^ APCs increase adipose tissue lipolysis

**Fig. 5:**
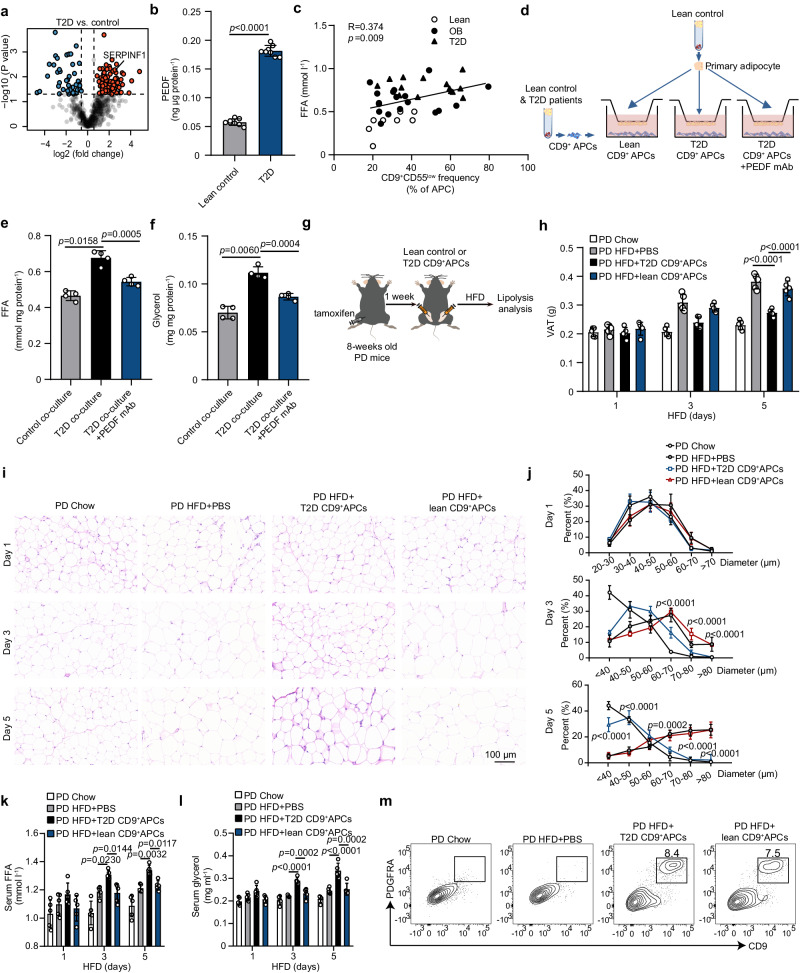
CD9^+^CD55^low^ APCs promote adipocyte lipolysis. **a** Volcano plot of differentially secreted proteins in culture medium of CD9^+^CD55^low^ APCs between T2D patients with obesity and lean control subjects. The increased proteins and decreased proteins are highlighted in red and blue. **b** The concentration of PEDF in the culture medium of CD9^+^CD55^low^ APCs from lean control individuals (*n* = 8) and T2D patients with obesity (*n* = 8). **c** Correlation between frequency of CD9^+^CD55^low^ APCs and serum FFA levels (*n* = 40). **d**–**f** Human primary adipocytes were isolated from lean control subjects and co-cultured with CD9^+^CD55^low^ APCs from lean control individuals or T2D patients with obesity; meanwhile, neutralizing PEDF antibody (25 μg ml^−1^) was added in the lower chamber. Three days after co-culture, culture mediums and adipocytes were collected. **d** Illustration of the co-culture experiments. **e,f** Levels of FFA (**e**) and glycerol (**f**) in culture medium (*n* = 4 per group), FFA, free fatty acid. For **g**–**m**, eight-weeks old PD mice were treated with tamoxifen to deplete APC. One week later, 1×10^6^ CD9^+^CD55^low^ APCs from lean control subjects and T2D patients with obesity were transferred in situ into eWAT of receipt mice, which were subsequently challenged with HFD. Serum and eWAT were collected at the indicated time points. **g** Schematic of the cell transplantation experiment. **h** eWAT weight of mice in three groups (*n* = 5 per group). **i** Representative H&E images of eWAT in three groups. **j**, Quantification of adipocyte diameter (*n* = 5 per group). **k,l** Levels of FFA (**k**) and glycerol (**l**) in serum of mice at each time point (*n* = 5 per group). **m** Five days after HFD feeding, the eWAT of receipt mice was collected to harvest SVF. Flow cytometry analysis was conducted to detect transplanted human APCs by staining with anti-human PDGFRA and anti-human CD9 antibodies. Representative plots indicate transferred human APCs in the eWAT of recipient mice. Data are means ± SD. The following tests were used. **a** Unpaired two side Welch’s *t* test. **b** Two-tailed unpaired Student’s *t* test. **c** Spearman’s bivariate correlation test (One-tailed) were used. **e**, **f**, **h**, **j–l** One-way ANOVA followed by Tukey’s multiple comparison test. Source data are provided as a Source data file.

Herein, elucidation of the APC secretome allowed us to gain a deeper insight into the functionality of CD9^+^CD55^low^ APCs during T2D progression. Surprisingly, by mining these proteins secreted by CD9^+^CD55^low^ APCs from T2D patients, we found that SERPINF1 was highly enriched (Fig. 5a). SERPINF1, also called pigment epithelium-derived factor (PEDF), promotes insulin resistance by inducing adipose lipolysis by binding to adipose triglyceride lipase^19,20^. To substantiate our DIA-MS data, we quantified PEDF levels in the culture medium of CD9^+^CD55^low^ APCs and found that the PEDF levels were markedly higher in T2D patients with obesity, compared to that in lean participants (Fig. 5b). Consistent with previous findings, the administration of recombinant PEDF protein triggered increased lipolysis, as illustrated by higher levels of glycerol and free fatty acids (FFA) in the culture media of PEDF-treated human adipocytes (Supplementary Fig. 5d, e). In the clinical cohort enrolled to detect APC alteration, the frequency of CD9^+^CD55^low^ APCs positively correlated with serum FFA levels (Fig. 5c). These observations suggest that CD9^+^CD55^low^ APCs directly regulate adipose lipolysis. To test this hypothesis, we performed in vitro co-culture experiments using human primary adipocytes and CD9^+^CD55^low^ APCs (Fig. 5d). We observed that the levels of FFA and glycerol were notably increased in the culture medium of primary adipocytes co-cultured with CD9^+^CD55^low^ APCs from T2D patients, which was notably reversed by the administration of a PEDF-neutralizing antibody (Fig. 5e, f).

To further verify the effect of CD9^+^CD55^low^ APCs on adipose lipolysis in vivo, CD9^+^CD55^low^ APCs from lean control subjects and T2D patients with obesity were sorted and transferred in situ into the eWAT of APC-depleted PD mice, which were subsequently fed chow or HFD for a short time (Fig. 5g). We found that body weight was slightly increased in the mice on HFD for 5 days compared to the chow-fed mice (Supplementary Fig. 5f). A weight reduction in eWAT in mice receiving diabetic CD9^+^APC transfer was observed compared to that in the mice transplanted with normal CD9^+^ APCs and mice injecting PBS (Fig. 5h). Notably, the eWAT of mice receiving diabetic CD9^+^APC transfer contained a higher proportion of small adipocytes at 3 and 5 days after HFD feeding compared to the mice receiving normal CD9^+^APC transfer and mice injecting PBS (Fig. 5i, j). Serum FFA and glycerol levels were significantly higher in the mice receiving diabetic CD9^+^APC transfer than in the other groups (Fig. 5k, l). Intriguingly, increased hepatic lipid deposition was observed in the livers of mice transplanted with diabetic CD9^+^ APCs, as well as increased liver triglyceride content (Supplementary Fig. 5g, h). As shown in Fig. 5m, transplanted APCs could be still detected in the eWAT of receipt mice at the end of treatment. Taken together, these data provided insights into the paracrine function of human CD9^+^CD55^low^ APCs and underscore the potentially important role of CD9^+^CD55^low^ APCs in promoting adipose lipolysis.

### Genetically depleting APCs improves obesity-related metabolic impairments

There are differences in APC heterogeneity among species. Before investigating the potential therapeutic effects of targeting APC in mice with obesity-related metabolic disorders, we compared APC heterogeneity between humans and mice. In contrast to human APCs, although mouse APCs could be clearly distinguished by CD9 and CD26, CD26^+^ APCs also expressed high levels of CD9 (Supplementary Fig. 6a). To gain deeper insights, CD9^+^CD26^+^ APCs and CD9^+^CD26^-^ APCs in the eWAT of C57BL/6 mice were sorted using flow cytometry and performed RNA-Seq analysis. After comparing the transcriptomes of mice APCs with identity genes of human scRNA-seq data, we found that both CD9^+^CD26^+^ APCs and CD9^+^CD26^-^ APCs in mice express a lot of identity genes of human *CD9*^*+*^ APCs (Supplementary Fig. 6b). Correlation analysis further showed that, transcriptional signature of mouse CD9^+^CD26^-^ APCs correlate with *CD9*^*+*^ APCs, while mice CD9^+^CD26^+^ APCs correlate with both human *CD9*^*+*^ APCs and *CD55*^*+*^ APCs (Supplementary Fig. 6c). Nevertheless, CD9^+^ APCs dramatically increased and constituted the predominant subpopulation in the eWAT of HFD-induced obese mice (Supplementary Fig. 6d, e).

**Fig. 6:**
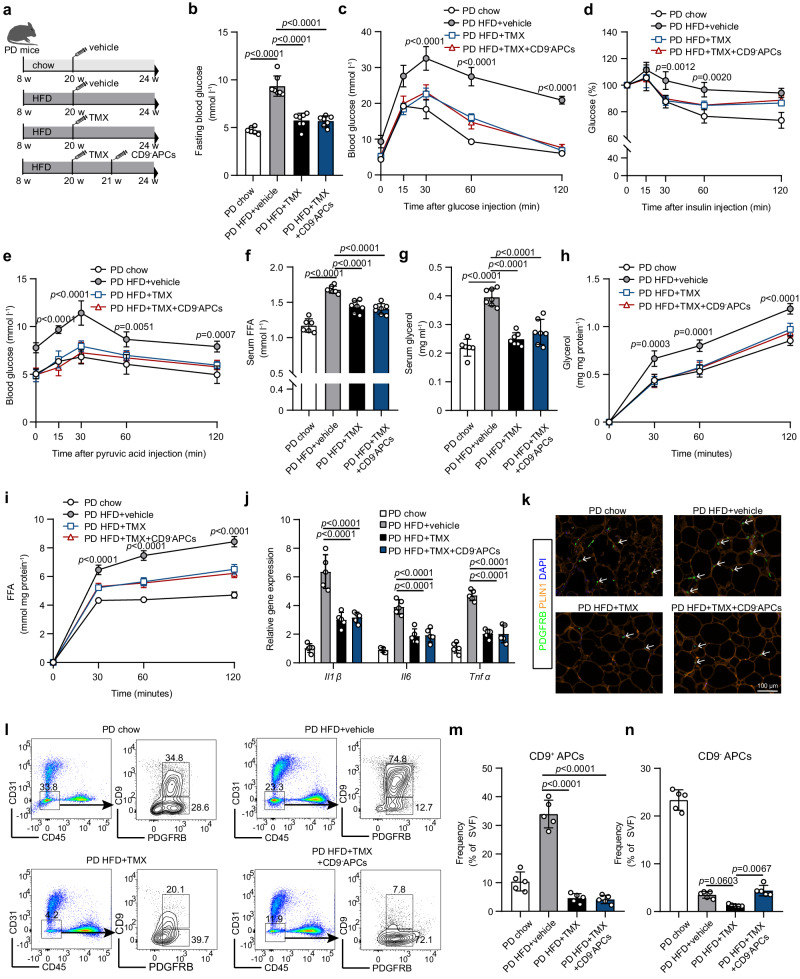
Genetically depleting APCs improves obesity-related metabolic impairments. Eight-weeks old PD mice were fed with HFD for 3 months and APCs were depleted to investigate the metabolic alterations. Meanwhile, about 3.5×10^4^ CD9^-^ APCs isolated from untreated obese PD mice were transplanted into the eWAT of APC-depleted mice. **a** Schematic representation of the intervention strategy. TMX, tamoxifen. **b** After one month, fasting blood glucose levels were measured (chow, *n* = 6; all other groups, *n* = 7). Glucose tolerance test (**c**), insulin tolerance test (**d**), and pyruvate tolerance test (**e**) in mice after 1 month of tamoxifen administration (*n* = 5 per group). Serum and eWAT of all mice were then collected to conduct the following experiments (**f–n**). Serum levels of FFA (**f**) and glycerol (**g**) (chow, *n* = 6; all other groups, *n* = 7). The eWAT of mice was cultured in vitro and levels of glycerol (**h**) and FFA (**i**) in culture medium were detected at the indicated time points (*n* = 5 per group). **j** mRNA level of the indicated genes was determined by qRT-PCR (*n* = 5 per group). **k** Representative images showing the staining of APCs in mouse eWAT of four group. White arrows indicate typical stained cells. **l**–**n** Frequencies of APC subpopulations in mouse eWAT of four group were detected by flow cytometry (**l**) and quantified (**m,n**) (*n* = 5 per group). Data are means ± SD. For statistical analysis, **b**–**j**, **m**, **n** One-way ANOVA was used followed by Tukey’s multiple comparison test. Source data are provided as a Source data file.

Next, we fed PD mice an HFD for 3 months to induce obesity and glycemic disturbances (Supplementary Fig. 6f, g), and then investigated the metabolic alterations caused by CD9^+^APC depletion. To minimize the influence of CD9^−^APC depletion on glycemic metabolism, we calculated the number of decreased CD9^−^ APCs in tamoxifen-treated obese PD mice and transplanted equivalent CD9^−^ APCs into APC depleted mice (Supplementary Fig. 6h, Fig. 6a). Food intake and body weight were not statistically different among groups of HFD-fed PD mice (Supplementary Fig. 6i-k). Compared to vehicle treated HFD mice, the fasting blood glucose levels were notably lower in APC-depleted mice and CD9^−^ APCs reconstituted mice (Fig. 6b). Furthermore, compared to vehicle treated HFD mice, APC-depletion markedly improved impaired glucose tolerance and insulin sensitivity, either CD9^−^ APCs were reconstituted or not (Fig. 6c, d). Meanwhile, pyruvate tolerance test (PTT) was also dramatically improved in the APC-depleted mice, suggesting the contribution of improved hepatic gluconeogenesis to improved whole-body glucose metabolism (Fig. 6e). The eWAT of APC-depleted mice contained a higher proportion of large adipocytes compared to the vehicle treated HFD mice (Supplementary Fig. 6l, m). Notably, serum FFA and glycerol levels, which significantly increased in the HFD-fed mice, were markedly reduced in APC-depleted mice and CD9^−^APC reconstituted mice (Fig. 6f, g). In vitro assay further confirmed that, fewer FFA and glycerol were released by eWAT from the APC-depleted than from the vehicle treated HFD mice, reflecting mitigated adipose tissue lipolysis (Fig. 6h, i). Additionally, APC depletion appeared to restore the expression of proinflammatory genes in eWAT to a degree similar to that in the chow-fed mice (Fig. 6j). APCs in eWAT were detected at the end of treatment to ascertain whether these metabolic benefits were due to APC depletion. As shown in Fig. 6k, the eWAT of APC-depleted mice contained fewer APCs than that of vehicle treated HFD mice. Flow cytometric analysis further indicated that the frequency of CD9^+^ APCs in the eWAT of the APC-depleted mice remained lower than that of vehicle treated HFD mice, whereas the frequency of CD9^−^ APCs in mice receiving CD9^−^APC transplantation was comparable to the vehicle treated HFD mice (Fig. 6l-n).

### Pharmacological elimination of APCs in eWAT improves obesity-related metabolic impairments

**Fig. 7:**
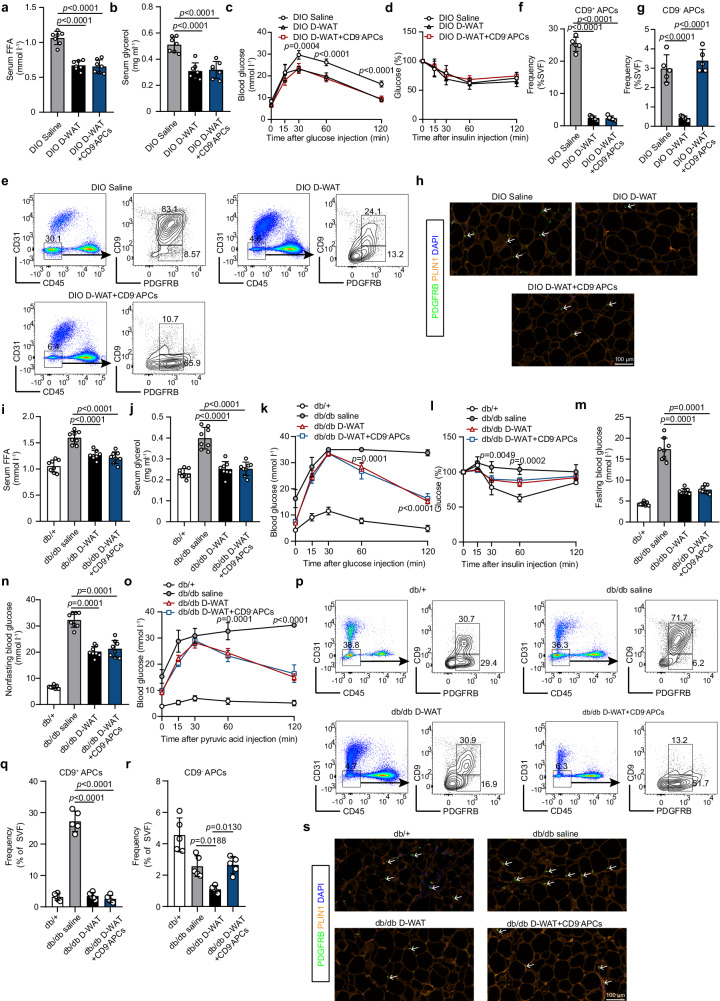
Pharmacological elimination of APCs in eWAT improves obesity-related metabolic impairments. **a**–**g** DIO mice were treated with D-WAT (8 mg per mouse) or same dosage of saline. Three days later, about 4×10^4^ CD9^-^ APCs were sorted from DIO mice and transplanted in situ into bilateral eWAT of D-WAT-treated mice. Two weeks after treatment, metabolic parameters were analyzed. Levels of FFA (**a**) and glycerol (**b**) in serum of DIO mice (*n* = 7 per group). **c** Glucose tolerance test (*n* = 5 per group). **d** Insulin tolerance test (*n* = 5 per group). **e**–**g** APC subpopulations in mouse eWAT of three groups were detected by flow cytometry (**e**) and quantified (**f,g**) (*n* = 5 per group). **g** Representative images showing the staining of APCs in mouse eWAT of three groups. White arrows indicate typical stained cells. **i**–**s** *Db/db* mice were treated with D-WAT (8 mg per mouse) or same dosage of saline. Normoglycemic control db/+ mice were also injected with saline. Three days after D-WAT treatment, about 3.5×10^4^ CD9^-^APCs were sorted from *db/db* mice and transplanted in situ into bilateral eWAT of D-WAT-treated mice. Four weeks after treatment, metabolic parameters were analyzed. Levels of FFA (**i**) and glycerol (**j**) in serum of *db/db* and control mice (*n* = 8 per group). **k** Glucose tolerance test (*n* = 5 per group). **l** Insulin tolerance test (*n* = 5 per group). **m,n** fasting blood glucose levels (**m**) and nonfasting blood glucose (**n**) in *db/db* and control mice (*n* = 8 per group). **o** Pyruvate tolerance test (*n* = 5 per group). **p**–**r** APC subpopulations in mouse eWAT of four groups were detected by flow cytometry (**p**) and quantified (**q**, **r**) (*n* = 5 per group). **s** Representative images showing the staining of APCs in mouse eWAT of four groups. White arrows indicate typical stained cells. Data are means ± SD. For statistical analysis, One-way ANOVA was used followed by Tukey’s multiple comparison test. Source data are provided as a Source data file.

To further explore the translational potential of targeting pathogenic APCs pharmacologically, peptide D-WAT was administered to two classical mouse models with obesity-related metabolic impairments, diet-induced obese (DIO) mice and *db/db* mice. Three days after D-WAT treatment, the number of decreased CD9^−^ APCs in D-WAT-treated mice was calculated (Supplementary Fig. 7a). To minimize the influence of CD9^−^APC depletion on glycemic metabolism, CD9^−^ APCs were sorted from untreated DIO mice and transplanted in situ into bilateral eWAT of APC depleted mice (Supplementary Fig. 7b). Food intake and body weight showed no statistical difference between three groups (Supplementary Fig. 7c, d). Compared to mice receiving saline injection, significantly reduced serum FFA and glycerol levels were observed in the serum of D-WAT-treated mice and CD9^−^APC reconstituted mice (Fig. 7a, b). Accordingly, D-WAT-treated mice and CD9^−^APC reconstituted mice showed improved glucose tolerance, although insulin sensitivity was not altered (Fig. 7c, d). Depletion of APCs in eWAT was sustained in D-WAT-treated mice, as indicated by flow cytometry and immunostaining analyses (Fig. 7e–h). Of note, CD9^−^APC transplantation restored the frequency of CD9^−^ APCs to a degree comparable to that of saline-treated mice (Fig. 7g).

Next, we determined whether D-WAT administration could also exert metabolically benefits in *db/db* mice. Similar to the intervention strategy in DIO mice, the number of decreased CD9^−^ APCs in D-WAT-treated *db/db* mice was calculated (Supplementary Fig. 7e). Next, CD9^−^ APCs were sorted from untreated *db/db* mice and transplanted into APC depleted mice (Supplementary Fig. 7f). Food intake and body weight showed not statistically difference among groups (Supplementary Fig. 7 g,h). One month after D-WAT treatment, APC depletion resulted in statistically significantly lower levels of serum FFA and glycerol in the D-WAT-treated *db/db* mice than in the saline-treated *db/db* mice (Fig. 7i, j). Furthermore, both impaired glucose tolerance and insulin sensitivity improved in D-WAT-treated *db/db* mice (Fig. 7k, l). Meanwhile, recovery of CD9^−^ APCs did not affect these metabolic benefits achieved by APC depletion. Importantly, compared to in the saline-treated *db/db* mice, the levels of non-fasting and fasting blood glucose were markedly decreased in the D-WAT-treated and CD9^−^APC reconstituted *db/db* mice (Fig. 7m, n). Intriguingly, the stimulation of gluconeogenesis in the liver also improved in the APC-depleted *db/db* mice (Fig. 7o). As illustrated by immunostaining and flow cytometry analysis, APCs in eWAT of the *db/db* mice remained reduced, whereas the frequency of CD9^−^ APCs was restored in mice receiving CD9^−^APC transplantation (Fig. 7p-s). In summary, these above findings demonstrate that APC depletion is sufficient to achieve metabolic benefits in obesity-related metabolic disorders.

## Discussion

APCs play a critical role in orchestrating tissue homeostasis and driving tissue dysfunction in health and obesity. Although some clues have been provided to link APC to adipose dysfunction, whether specific APC subpopulations contribute to T2D development and the underlying mechanisms remain poorly understood. Herein, through scRNA-seq and experiments in human cohorts and mouse models, we showed that CD9^+^CD55^low^ APCs are molecularly and functionally distinct subpopulations that contribute to the development of T2D. Dysfunctional CD9^+^CD55^low^ APCs promote local and systemic metabolic deterioration through multi-layered pathogenic mechanisms involving the secretion of various bioactive proteins. Notably, genetic or pharmacological depletion of dysfunctional CD9^+^CD55^low^ APCs improves obesity-related metabolic deterioration, representing as a promising therapeutic target to combat T2D.

To determine which APC subpopulation contributes to the development of obesity-related glycemic disturbances, we performed scRNA-seq using omental adipose tissues from a cohort of lean individuals, participants with obesity and T2D patients with obesity. Heterogeneity of APC has been firmly delineated using single-cell technologies^6,7,10,15,16^, whereas clustering differs between previous studies, which may be ascribed to differences in the adipose depot, metabolic status, or species. Owing to enrolled T2D patients which previous studies do not include, we identified CD9^+^CD55^low^ APCs as a detrimental subpopulation in diabetes that correlate with T2D development. As further depicted by WGCNA, the reshaping of the transcriptional profile mostly occurred in CD9^+^CD55^low^ APCs, with the remaining subpopulations relatively quiescent. These findings suggest that the excessive expansion and functional alteration of CD9^+^CD55^low^ APCs participate in T2D development, which was further confirmed by in vivo cell transplantation experiments conducted in two mouse models. Supporting our data, a previous study observed an association between an increased number of APCs expressing CD9 and glycemic disturbance^14^. Thus, our data and previous studies suggest that APCs are potential therapeutic targets for treating obesity-related metabolic disorders. We found that APC depletion using either genetical or pharmacological methods resulted in impressive metabolic benefits in obesity. Although these two methods could not specifically deplete CD9^+^CD55^low^ APCs, we and previous studies observed that the majority of APCs were CD9^+^ cells in obese adipose tissue^10,13^. Importantly, reconstitution of CD9^−^ APCs, which retain adipogenic potential, do not affect the metabolic benefits achieved by APC depletion (Supplementary Fig. 8). Of note, in APC-depleted PD and *db/db* mice, total APC transplantation reversed metabolic improvement by APC depletion, indicating that effects of APC depletion are ascribed to specific effect of cell ablations (Supplementary Fig. 8). Thus, the metabolic benefits achieved after APC depletion in obesity may be mainly attributed to the clearance of pathogenic APCs. Intriguingly, a recent study showed that the clearance of senescent cells in adipose tissue improves metabolic dysfunction in obese mice, while APCs constituted a large proportion of eliminated senescence cells^21^. Although it is not clear whether these depleted APCs were mainly CD9 positive cells in their study, an activated senescence pathway in CD9^+^ APCs from T2D patients was observed in our dataset. Nevertheless, mouse models that specifically targeting the CD9^+^APC subpopulation are required. In addition, it is worth noting that APCs are critical to generate new adipocytes during the progression of obesity^22–24^, whether APC depletion impedes adipocyte generation needs further investigation. Moreover, depleting APCs at different stage of obesity may cause diverse consequences. Depleting APCs in subcutaneous adipose tissue through subcutaneously injection of D-WAT before the development of obesity could induce beige adipocyte differentiation, while long-term APC depletion causes adipocyte hypertrophy and tissue fibrosis^17,18^. Taken together, our data suggested that CD9^+^CD55^low^ APCs are novel and potentially translatable targets for T2D interventions.

The secretory proteome of CD9^+^CD55^low^ APCs, delineated by DIA-MS based analysis, expanded current insights into the functionality of human APCs, which are mainly established at the RNA level^16,25^. A recent study that performed proteomic analysis of murine APCs also proposed that descripting the functional heterogeneity of APCs at the protein level is more important than that at the transcriptional level^26^. In contrast to previous studies, we focused on the secreted proteins of pathogenic APCs related to T2D development. This is of particular importance in elucidating the abnormal communications between APC and other cell types in the development of adipose dysfunction; a recent study predicted that increased cellular interactions in obesity mainly occur between APC and other cell types^16^. For instance, a broad interactome was observed based on scRNA-seq analysis, especially between APCs and macrophages, which is consistent with recent studies^27^. Based on our secretome data, MDK was found to be an APC-derived chemokine, providing additional insights into the chemotaxis activity of APC. Regarding the immunoregulatory function of APCs, proteins produced under steady state, such as IL-33 and GDNF, may facilitate the maintenance of immune homeostasis^11,28^, while these aberrantly secreted proteins may be responsible for the development of local immune disturbances. Among the proteins secreted by CD9^+^CD55^low^ APCs, various have been shown to participate in immune disturbances and metabolic deterioration. For example, GRN has been reported to be an adipokine that correlates with macrophage infiltration and mediates HFD-feeding-induced insulin resistance^29,30^. GAS6, a secreted ligand for tyrosine kinase receptor, has been implicated in the control of innate immunity and inflammation^31,32^. Several proteins belonging to the IGFBP family, which may modulate insulin sensitivity by regulating the bioactivity of IGFs or through IGF-independent actions^33–35^. Moreover, these proteins may have non-immunoregulatory functions. CILP1, shown to promote myofibroblast proliferation in a mouse model of pathologically cardiac remodeling^36^, may serve as a signal to promote APC expansion in an autocrine manner during obesity progression. Another matricellular protein, CCN3, has been reported to be an adipokine involved in obesity-associated insulin resistance^37^. Nevertheless, the roles of most proteins abnormally secreted by CD9^+^CD55^low^ APCs in metabolic regulation remain unclear. In particular, whether some proteins are “pre-adipokines” and mediate organ communication deserves further investigation.

The bioactive proteins secreted by diabetic CD9^+^CD55^low^ APCs may also form a pathogenic microenvironment that influences other non-immune cell types in adipose tissue. In support of this notion, another interesting finding of this study is that CD9^+^CD55^low^ APCs from T2D patients promote adipose lipolysis, based on the strength of in vitro and in vivo experiments in both human and mouse models. This study provides direct experimental evidence demonstrating the role of APCs in regulating mature adipocyte function, although the cellular interactions between APCs and adipocytes have been recently predicted^16^. Unregulated lipolysis of hypertrophic adipose tissue is a key pathological manifestation of obesity, which leads to the aberrant accumulation of lipids in peripheral metabolic tissues and increased hepatic gluconeogenesis^38–41^. In agreement with increased adipose lipolysis, aberrant lipid deposition in the liver and increased hepatic gluconeogenesis were detected in APC-treated mice in this study, whereas APC depletion reversed these pathological manifestations. This may explain the impaired glycemic tolerance after APC transplantation and the improvement in glycemic intolerance after APC depletion. Further, besides their direct effect on lipolysis, CD9^+^CD55^low^ APCs may influence other functions of adipocyte. Similarly, our in vitro co-culture assay showed that gene expression of some adipokines in human primary adipocytes was altered after co-culture with CD9^+^CD55^low^ APCs from T2D patients, suggesting a potential role of these pathogenic APCs in the regulation of adipocyte endocrine function (Supplementary Fig. 9). Intriguingly, immunostaining showed that many CD9^+^ APCs were distributed around the adipocytes, indicating tight connections between these two cell types (Supplementary Fig. 8). In the future, experimental studies are needed to elucidate the detailed mechanisms by which APCs affect adipocyte function.

In addition, we analyzed the adipogenic properties of different human APC subpopulations. In contrast to the findings in murine studies^6,10^, all human APC subpopulations exhibited adipogenic potential when treated with standard differentiation factors in vitro (Supplementary Fig. 8). Consistent with our findings, a previous study showed that human APC subpopulations retain similar adipogenic potential but give rise to adipocytes with distinct properties^15^. In this regard, the expanded reservoir of APCs may facilitate adipogenesis in the context of obesity, but may be inhibited under local micro-circumstances. Furthermore, our data and previous studies^10,14^ all showed that CD9^+^ APCs promote adipose tissue fibrosis, which might be regulated by TGFβ signaling (Supplementary Fig. 8). In addition, in inguinal adipose tissue of aged mice, impaired de novo differentiation of APC into beige adipocyte in response to cold exposure was related to upregulation of CD9 and other fibrotic genes^42^.

One limitation of this study is that with genetically depleting APCs in mice, we could not exclude the contribution of other metabolic organs. Although we provide evidence that depleting APCs in adipose tissue using D-WAT is sufficient to improve obesity-related metabolic dysfunction, novel therapeutic methods specifically targeting CD9^+^CD55^low^ APCs in visceral adipose tissue need exploration. In addition, as broad interactions between APCs and other non-immune cell components are predicted in adipose tissue, further functional studies are required to identify the biomatter mediating these communications and clarify the underlying mechanisms. Besides, although the role and underlying mechanism of CD9^+^CD55^low^ APCs in diabetes are disclosed, altered transcriptional signatures are also observed in other APC subpopulations. How the cellular fate changes and to what extent these alterations affect systemic metabolism need further investigation.

In conclusion, our findings provide insights into human APC heterogeneity and reveal the crucial role of dysfunctional CD9^+^CD55^low^ APCs in T2D development. These dysfunctional CD9^+^CD55^low^ APCs trigger a series of tissue impairments with profound and deleterious effects on metabolic modulation. Targeting these dysfunctional CD9^+^CD55^low^ APCs may provide a novel strategy for restoring adipose function and systemic metabolism.

## Methods

### Study participants

Overall, 48 subjects, including 37 participants with obesity who underwent laparoscopic Roux-en-Y gastric bypass (RYGB) surgery and 11 lean controls who underwent elective abdominal surgery, were enrolled in this study. None of the participants had a history of diabetes, and 14 participants with obesity were diagnosed with T2D according to the American Diabetes Association criteria^43^. All participants completed questionnaires for medical history assessment and underwent anthropometric measurements. BMI was calculated as weight divided by height in meters squared. Exclusion criteria were: acute infections or steroid treatment in the past three months; smoking or myocardial infarction; autoimmune diseases, such as rheumatoid arthritis and systemic lupus erythematosus; chronic digestive diseases; malignant tumors; abnormal liver or renal dysfunction; pregnancy; other endocrine system diseases, such as thyroid diseases and Cushing’s syndrome.

This study was approved by the Ethics Review Committee of Nanjing Drum Tower Hospital Affiliated to Nanjing University Medical School (approval number: 2017-030-08). At enrollment, written informed consent was obtained from all participants.

### Animal study

The following strains were used in this study: C57BL/6, *db*/*db*, and *Pdgfra*-CreERT2;DTA^flox/−^ mice. All animals were purchased from GemPharmatech, and *Pdgfra*-CreERT2;DTA^flox/−^ mice were generated by crossing *Pdgfra*-CreERT2 mice (stock number T052695) with Rosa26-LSL-DTA mice (stock number B009762). All mice were kept in a SPF level facility and provided with adequate food and water, as well as normal light, temperature and humidity (12-h light/dark cycle, 60-70% humidity). *Pdgfra*-CreERT2;DTA^flox/−^ mice were intraperitoneally injected with tamoxifen (2 mg day^-1^ per mouse) for 5 days, and other treatments were performed as indicated in the text.

To pharmacologically deplete APCs or transfer human APCs into eWAT of receipt mice, in situ injection was performed as described before^44^. In brief, to pharmacologically deplete APCs in the eWAT of C57BL/6 mice, mice were anesthetized and abdominal midline incision in skin was made. Then, the abdominal wall was carefully cut open to expose the eWAT. A total of 10 μl of D-WAT (0.2 mg μl^-1^) or saline was injected (2 μl per injection) to five different positions along each fat pad using a Hamilton syringe, and the needle was left in place for 1 additional minute. After injection, the abdominal wall and skin incisions were sutured. For DIO and *db/db* mice, 20 μl of D-WAT (0.2 mg μl^-1^) or saline was evenly administrated (2 μl per injection) along each fat pad. After D-WAT or saline administration, mice were utilized for further treatment as indicated in the text. For cell transplantation assay, operations were carried out following the same procedure, except freshly isolated cells were injected into the eWAT of receipt mice as detailed described below. As previously described^18^, the peptide D-WAT (_d_CSWKYWFGEC-_d_KLAKLAK_2_) was synthesized by GL Biochem (Shanghai, China).

All mice utilized in the experiments were males. All the animal procedures were approved by the Research Animal Care Committee of Drum Tower Hospital Affiliated to Nanjing University Medical School (approval number: 2019AE01002). CO_2_ euthanasia was performed according to the AVMA Guidelines for the Euthanasia of Animals (2020 Edition).

### Blood sample analysis

Fasting blood samples were collected for HbA1c, triglycerides, total cholesterol, high-density lipoprotein cholesterol, low-density lipoprotein cholesterol, and FFA measurements. Blood glucose concentration was assessed by the hexokinase method on TBA-200FR (Tokyo, Japan). HbA1c was quantitated by high-performance liquid chromatography (Bio-Rad D-10). Concentrations of triglyceride, total cholesterol, high-density lipoprotein cholesterol, and low-density lipoprotein cholesterol were determined by immunoassays (Cobas e601, Roche). Serum FFA levels were assessed by an enzymatic method (DiaSys Diagnostic Systems).

### scRNA-seq

As for scRNA-seq assay, periumbilical adipose tissue samples (about 10 g) at the omental region were collected from 2 lean control subjects, 3 participants with obesity and 3 newly-diagnosed T2D patients with obesity. Adipose tissue samples were transported to laboratory on ice immediately, cut into small pieces and digested with 0.1% type II collagenase (Gibco, 17100-015) for about 50 minutes. After centrifugation, the resulting supernatants were aspirated and the remaining pellets were resuspended in red blood cell (RBC) lysis buffer (Thermo, 00-4300) for 5 minutes. These SVF samples were washed with PBS and went to the procedure of cell capture and cDNA synthesis. Using single cell 3’ Library and Gel Bead Kit V3 (10×Genomics, 1000075) and Chromium Single Cell B Chip Kit (10×Genomics, 1000074), the SVF suspension (300-600 living cells per microliter determined by Count Star) was loaded onto the Chromium (10×Genomics) single cell controller to generate single-cell gel beads in the emulsion following the manufacturer’s instruction. In brief, single cells were suspended in PBS containing 0.04% BSA. Approximately 50000 cells were added to each channel and the target cell will be recovered as estimated to be about 10000 cells. Captured cells were then lysed and the released RNA were barcoded via reverse transcription in individual GEMs. Reverse transcription was conducted on a S1000TM Touch Thermal Cycler (Bio Rad) at 53 °C for 45 minutes, followed by 85 °C for 5 minutes, and hold at 4 °C. The generated cDNA was then amplified and quality assessed using an Agilent 4200. Next, following the manufacture’s protocol, Single-cell RNA-seq libraries were constructed using Single Cell 3’ library and Gel Bead Kit V3. The libraries were finally sequenced using an Illumina Novaseq6000 sequencer with a sequencing depth of at least 50000 reads per cell with pair-end 150 bp reading strategy.

### scRNA-seq data analysis

Raw reads processing was performed with the standard pipelines of Cell Ranger (version 3.0.2, 10x Genomics) and mapped to the GRCh38 human reference genome. The preliminary gene expression matrixes were then analyzed using the R package Seurat v3.2.2^45^ and cells with <200 or >6000 detected genes, with <300 or >40000 UMI counts were filtered out, as well as cells that contained > 30% mitochondrial gene counts. The Python package Scrublet v0.2.3^46^ was used with default parameter to remove predicted doublets. After quality control, a total of 55,705 cells from 8 samples were remained for downstream analysis.

The standard workflow in Seurat was used for dimension reduction and unsupervised clustering. Briefly, UMI counts measuring gene expression were log-normalized. The top 2000 highly variable genes (HVGs) were detected using ‘Find Variable Features’ function. The data were scaled by regressing out the variation in total UMI counts, percentages of mitochondrial or ribosomal gene counts. Then, principal component analysis (PCA) was performed based on HVG expression, and the R package Harmony v1.0^47^ was used to eliminate the batch effect. With using the top 20 Harmony principal component (PCs), clusters were identified with the ‘Find Neighbors’ and ‘Find Cluster’ functions in Seurat, and visualized with the uniform manifold approximation and projection (UMAP) algorithm. After the first-round of unsupervised clustering, we annotated major cell types including APCs, mesothelial cells, endothelial cells, adipocytes, T/natural killer (NK) cells, myeloid cells, B cells and mast cells according to canonical known cell markers. To identify cell clusters within each major cell type, we performed a second-round of unsupervised clustering on APCs, NK/T cells and myeloid cells respectively.

### Marker genes identification and cell-type annotation

Cell cluster-specific marker genes were identified using the ‘Find All Markers’ function in Seurat package. In brief, each cell cluster was compared with the rest clusters using the following parameters: min.pct=0.2, logfc.threshold = 0.25, only.pos = T. *P*-values was obtained by Wilcoxon rank-sum test and adjusted based on Bonferroni correction. We used heatmap to visualize marker genes based on gene expression after the log-transformed and scaling.

To compare with previously identified clusters of APCs, we downloaded the representative gene signatures of each cluster to be compared from the public data^6,7,16^ and calculated the cluster score as the average log2 (LogNormalizedUMI+1) of all genes in one signature.

### Integration of published APCs datasets

For data integration, we obtained three publicly available APCs datasets, each accompanied by associated metadata defining cell subclusters^6,7,16^. Initially, we transformed genes of two mouse datasets into homologous human genes. Subsequently, we conducted dataset normalization, identified highly variable genes, and employed Seurat’s SelectIntegrationFeatures () function to combine these genes across datasets. Following this, we performed data scaling and PCA analysis for each dataset. To harmonize the datasets, we employed the Reciprocal PCA (RPCA) method, projecting each dataset into the PCA space of the others while maintaining consistent neighborhood criteria. Then, we performed PCA analysis and UMAP dimensionality reduction visualization on the integrated data. To preserve the original cell definitions from the source publications, we applied color coding based on the respective cell cluster definitions. To compare and assess the similarities and differences among our APCs clusters and those from publicly available datasets, we downloaded representative gene signatures for each cluster from publicly available datasets and computed cluster scores for our APCs clusters by calculating the average log2 (LogNormalizedUMI+1) of all genes within a given signature.

### Single cell trajectory analysis

We used R package Monocle2 v2.14.0^48^ and Monocle3 v0.2.2^49^ to determine the trajectory of APCs. Data preprocessing and pseudotemporal ordering of APCs were conducted using Monocle2. Briefly, clusters of APCs were loaded and created as an object by the newCellDataSet function within Monocle2 with the parameter expressionFamily = negbinomial.size. Genes with an average expression level ≥ 0.1 were preserved, and the top 500 signature genes for pseudotemporal cell sorting were determined using the differentialGeneTest functions within Monocle2. Dimensionality reduction was accomplished using the reduceDimension() function, with parameters reduction_method = “DDRTree” and max_components = 2. Subsequently, the minimum spanning tree of cells was visualized by utilizing the “plot_cell_trajectory” function within Monocle2. Monocle3, incorporating graph theory and dimensionality reduction, was employed to generate a graph-based visualization of the trajectories. We also applied R package Slingshot v1.4.0^50^ to confirm the trajectory result from the Monocle3. The corresponding UMAP matrix was fed into Slingshot, considering *CD55*^*+*^ APCs as a root state when calculating the trajectories and the pseudotime.

### Analysis of differentially expressed genes

Differentially expressed genes (DEGs) between any two groups in each APC cluster were identified by using ‘Find Markers’ function in Seurat. In any given comparison, only genes that met the following filtering thresholds were considered DEGs: (1) at least 25% of cells express the gene in either population, (2) at least 25 cells per group compared, (3) log2 average expression difference greater than 0.25, and (4) an adjusted *P*-value less than 1E-5. The Wilcoxon rank-sum test was used to obtain *P*-values for comparisons, and the adjusted P-values based on Bonferroni correction were calculated. We combined all the DEGs for downstream Weighted Correlation Network Analysis (WGCNA).

### WGCNA

The genes co-expression network was constructed by using the R package WGCNA v1.70-3^51^. A total of 12 cell groups from 4 APC clusters (*CD55*^*+*^ APCs, *CD9*^*+*^ APCs, *ICAM1*^*+*^ APCs, *CD142*^*+*^ APCs) in 3 subject status were loaded as ‘traits’ and the associated gene expression matrix was used to calculate Pearson’ s correlation matrices representing co-expression similarity of genes. The weighted adjacency matrix was created using the Pearson correlation coefficient test and transformed into a topological overlap measure (TOM) matrix to minimize the effects of noise and spurious associations. Then, a clustering dendrogram of genes was constructed based the average linkage hierarchical clustering of the TOM matrix. The minimal gene module size was set to 30 and the threshold to merge similar modules was set to 0.25. Finally, the different module eigengenes (MEs) were correlated with the above traits to find gene modules associated with clusters or diseases.

### Functional annotation of genes

We used “enrich GO” and “enrich KEGG” function in R package cluster Profiler v3.14.3^52^ to performed Gene Ontology (GO) analysis and Kyoto Encyclopedia of Genes and Genomes (KEGG) pathway enrichment analysis of cluster marker genes or DEGs. The results are annotated along ontology of biological processes with the following parameters: pvalueCutoff  =  0.01, pAdjustMethod = “BH” (Benjamani and Hochberg) and qvalueCutoff  =  0.05.

### Cell interaction analysis

The python package CellPhone DB v2.0.0^53^ was used to find ligand-receptor interactions between APCs clusters and other major cell types. Briefly, as suggested by the developers, single-cell data from curated seurat object was exported into text files of gene expression counts and metadata. Then, the data was processed in CellPhone DB command using default settings with 1000 statistical iterations. Significant interactions were considered as having a *P*-value < 0.01.

### Flow cytometry and cell sorting

Human adipose SVFs were prepared as described above. Mouse adipose SVFs were prepared using 0.1% type I collagenase (Gibco, 17100-017). Briefly, eWAT was physically dissociated using scissors and incubated for 45 minutes in digest solution (1 mg ml^-1^ type I collagenase in RPMI supplemented with 5% fetal calf serum, 1% L-glutamine, 1% penicillin-streptomycin, and 10 mM HEPES). Resulting dissociated tissue was passed through 100μm nylon mesh, centrifuged, and adipocytes were removed from the supernatant. Red blood cells were lysed using RBC lysis buffer.

Human adipose SVFs were stained with the following antibodies for flow cytometry analysis and cell sorting: CD45 (HI30, Biolegend, 1:100), CD31 (WM59, BD Biosciences, 1:100), PDGRFA (aR1, BD Biosciences, 1:100), CD9 (M-L13, BD Biosciences, 1:100), CD55 (IA10, BD Biosciences, 1:100), ICAM1 (HA58, eBioscience, 1:100), and CD142 (HTF-1, BD Biosciences, 1:100). For animal studies, the following fluorophore-conjugated antibodies were used: CD45 (30-F11, BD Biosciences, 1:100), CD31 (MEC13.3, BD Biosciences, 1:100), PDGRFB (APB5, Biolegend, 1:100), CD9 (KMC8, BD Biosciences, 1:100), and CD26 (H194-112, BD Biosciences, 1:100). Dead cells were stained with fixable viability stain 780 (BD Biosciences, 1:1000) and removed. The stain protocol as used as described previously^54^. In brief, prepared human or mouse SVF suspensions were washed with PBS and resuspended in FACS buffer (2% FBS, 2 mM EDTA, 0.05% NaN_3_ in PBS), following by staining with the antibodies indicated in figures in FACS buffer for 30 minutes at 4°C. After washing with FACS buffer, the stained cells were resuspended in 400 μl of FACS buffer and analyzed on an LSR Fortessa II flow cytometer (BD Biosciences) or sorted on a FACSAria II flow cytometer (BD Biosciences). The gating strategies for flow cytometry analysis are shown in figures. All flow data were acquired by BD FACSDiva software and analyzed by FlowJo software version 10.8.1 (Tree Star, Inc).

### Bulk mRNA-seq and data analysis

Bulk mRNA sequencing was performed using a SMART-Seq HT Kit following the manufacturer’s protocol (Takara, 634436). In brief, FACS-sorted APC subpopulations were centrifuged at 50×*g* for 5 minutes, and washed with PBS for one time. About 3000 cells of each sample were pipetted into a new tube and centrifuged at 50×*g* for 5 minutes. Then, 10.5 μl 10× lysis buffer and 0.5 μl RNase inhibitor were added into each tube. The tubes containing reaction mixtures were stored in -80 °C until cDNA synthesis. We applied the HISAT2 v2.2.1 (https://github.com/DaehwanKimLab/hisat2) tool to map raw sequences to reference genome (human: GRCh38 version; mouse: mm10 version). Stringtie v2.2.1 software (https://ccb.jhu.edu/software/stringtie/) was used to generate the gene counts matrix and quantitate the gene expression as transcripts per million (TPM). Standard DEseq2 v1.26.0 workflow was then used to identify DEGs between two different conditions and the *P*-values are attained by the Wald test and corrected (padj) for multiple testing using the Benjamini and Hochberg method by default. The DEGs met the criteria for |log2-fold change | > 0.58 (equivalent to 1.5-fold change) and *P*-value < 0.05, and were visualized by heatmap based on log-transformed and scaled gene expression. The built-in R function ‘prcomp’ was used to perform PCA for multiple datasets, and the first two PCs were visualized by scatter-plot.

### Cell transplantation

Eight-weeks old C57BL/6 mice were pretreated by injecting peptide D-WAT into bilateral eWAT under isoflurane inhalation. Three days later, CD9^+^CD55^low^ APCs and CD9^−^ APCs were freshly sorted from the omental adipose tissue of T2D patients with obesity using a BD FACSAria II. Sorted cells were washed one time with PBS and pelleted at 400×*g* for 5 minutes. Then, approximately 1×10^6^ CD9^+^CD55^low^ APCs or CD9^−^ APCs were suspended in 20 μl of PBS and transferred in situ into bilateral eWAT of each receipt mouse.

For PD mice, 8-weeks old mice were injected intraperitoneally with tamoxifen for 5 days and allowed to recover for 1 week. Then, CD9^+^CD55^low^ APCs and CD9^−^ APCs were freshly sorted from the omental adipose tissue of T2D patients with obesity or lean subjects by flow cytometry. After one wash with PBS, approximately 1×10^6^ CD9^+^CD55^low^ APCs or CD9^−^ APCs were resuspended in 20 μl of PBS and transferred in situ into the bilateral eWAT of each recipient mice.

For intervention assays, CD9^−^ APCs were freshly sorted from the eWAT of obese PD, DIO and *db/db* mice. After one wash with PBS and pelleted, CD9^−^ APCs were resuspended in 20 μl of PBS and transferred in situ into the bilateral eWAT of the recipient mice as indicated in the text.

### Glucose, insulin, and pyruvate tolerance tests

For the glucose tolerance test (GTT), mice were fasted for 12 hours before intraperitoneal injection of a 20% glucose solution (2 g kg^-1^ body weight). Blood samples were obtained from the tail tip, and glucose levels were measured using a glucometer (Abbott). For *db*/*db* mice, mice were injected with a glucose solution (1 g kg^-1^ body weight) after fasting overnight. For the insulin tolerance test (ITT), mice were fasted for 4 hours before insulin (0.5 U kg^-1^ body weight) was intraperitoneally injected. Blood samples were collected and glucose levels were measured at the indicated time points. For pyruvate tolerance test (PTT), the mice were fasted overnight (12 hours) and injected with sodium pyruvate (1 g kg^-1^ body weight). Blood samples were collected from the tail vein at the indicated time points.

### DIA-MS analysis

Approximately 5×10^6^ CD9^+^CD55^low^ APCs from lean controls and T2D patients with obesity were freshly isolated using flow cytometry and cultured in serum-free PAM for 48 hours. The conditioned mediums were collected and centrifuged at 12000×*g* for 15 minutes. The supernatants were stored at -80 °C until peptide extraction. The re-dissolved peptides of each sample were analyzed using a nano Elute UHPLC (Bruker Daltonics, Germany) coupled to a timsTOF Pro (Bruker Daltonics, Germany) equipped with a CaptiveSpray ion source. Peptide powder was reconstituted in buffer A (0.1% formic acid in water). Peptide digests were separated at a flowrate of 300 nl min^-1^ using 60 minutes gradient on a 15 cm analytical column (75 μm ID, 1.9 μm, C18 beads, homemade) with an integrated Toaster column oven at 50 °C. Mobile phase B contained 0.1% formic acid in CAN. The B phase was increased from 5-27% in 50 minutes, 27-40% in 10 minutes, and 40-80% in 2 minutes, and was sustained at 80% for 3 minutes. The timsTOF Pro was operated in a positive ion data-dependent acquisition Parallel Accumulation Serial Fragmentation (PASEF) mode. The capillary voltage was set at 1400 V. The MS and MS/MS spectra were acquired from 100-1700 m/z, and an ion mobility range (1/K0) from 0.7-1.3 Vs/cm2. The ramp and accumulation times were set to 100 ms to achieve a duty cycle close to 100%. To perform the diaPASEF acquisition, we defined 28 Th isolation windows from m/z 384 to 1059. Ingenuity Pathway Analysis (IPA) software was used to analyze the data.

### Monocyte migration assay

In vitro monocyte migration assay was conducted using human blood CD14^+^ monocytes isolated from PBMC samples. PBMC were obtained from T2D patients using vacutainer cell preparation tubes (Becton Dickinson, 362761), according to the manufactory’s instructions. Then, human CD14 positive selection kit (STEMCELL Technologies, 17858) was used to isolate monocytes from PBMC. Briefly, about 1×10^7^ PBMC cells were labeled with 100 μl selection cocktail for 10 minutes. Next, 100 μl magnetic particles were added and mixed. Three minutes later, the recommended medium was added to the sample at the indicated volume and placed into the magnet for another 3 minutes. Unwanted cells were removed by removing the supernatant, and the remaining cells were washed twice with stain buffer. Then, isolated monocytes were resuspended in RIPA 1640 medium at a density of 3×10^5^ cells μl^-1^. Co-culture insert with an 8.0 μm permeable membrane (Costar, 3422) was used to perform cell migration assay. Approximately 3×10^4^ monocytes were seeded in the upper chamber, and the medium containing recombinant protein or conditioned culture medium of human CD9^+^CD55^low^ APCs was placed in the lower chamber. Twenty-four hours later, the migrated monocytes were fixed with 4% PFA for 2 minutes, permeabilized with absolute methanol for 15 minutes, stained with 1% crystal violet for 10 minutes, and photographed under a microscope (Leica), and counted using Image J software (version 1.52 v). The following recombinant proteins were used: MDK (Sino Biological, 10247-HNAB), APP (Sino Biological, 10703-H02H), and GRN (Sino Biological, 10826-H08H).

For the in vivo monocyte infiltration assay, 1×10^6^ CD9^+^CD55^low^ APCs from lean control subjects and T2D patients with obesity were isolated by flow cytometry and transferred into the unilateral eWAT of C57BL/6 mice. Monocytes from the blood of 10-week-old Rosa26-tdTomato mice were isolated using flow cytometry. Monocytes were counted, and 5×10^5^ viable cells were resuspended in PBS and injected into C57BL/6 mice receiving APC transfer via the tail vein. Two days later, the Tomato^+^ cells in the eWAT of recipient C57BL/6 mice were analyzed by immunofluorescence.

### Enzyme-linked Immunosorbent Assay (ELISA)

To determine the concentrations of PEDF in the culture medium of human CD9^+^CD55^low^ APCs, an ELISA kit was used following the manufacturer’s protocol (CUSABIO, CSB-E08818h). ELISA was performed according to the manufacturer’s protocol to determine the levels of FFA (Sigma, MAK044) and glycerol (Sigma, TR0100) in mouse serum. For mouse liver triglyceride analysis, the triglyceride content in the harvested liver tissue was determined using an ELISA kit following the manufacturer’s protocol (Applygen, E1013-50).

### Isolation of human primary adipocyte and in vitro co-culture assay

Primary human adipocytes were isolated as previously described^55^. Briefly, omental adipose tissue samples from T2D patients were digested in a manner similar to the SVF isolation protocol. After tissue digestion, samples were centrifuged at 50×*g* for 5 minutes. Next, the floating adipocyte fractions were carefully passed through a 250 μm strainer, and washed twice with RIPA 1640 medium. After centrifugation at 50×*g* for 5 minutes, packed adipocytes were carefully collected and used as indicated in the text. The recombinant PEDF protein used in this study was purchased from Novoprotein (P36955).

For the co-culture assay, a 6.5 mm Transwells (Costar, 3413) was used. CD9^+^CD55^low^ APCs from lean control participants and T2D patients with obesity were isolated using flow cytometry. 1×10^5^ CD9^+^CD55^low^ APCs were seeded at the bottom of each well, with neutralizing PEDF antibody (XpressBio, AB-PEDF1) added in separate group. Packed primary adipocytes (30 μl, about 60000 cells) isolated from lean control participants were pipetted onto the membrane of culture insert and placed into the respective well. Three days after co-culture, the culture mediums and adipocytes were collected.

### Immunohistological and immunofluorescence analysis

Adipose, liver, and pancreas tissues were washed with PBS, fixed in paraformaldehyde, paraffin embedded, and sliced into 4μm sections. These sections were stained with hematoxylin and eosin. The stained images were captured and digitalized using an Olympus microscope (Olympus, Japan).

For immunofluorescence, after deparaffinization and rehydration, the adipose tissue sections were incubated with xylene for 10 minutes and dehydrated in pure ethanol for 5 minutes, followed by dehydration in an ethanol gradient of 85% and 75% ethanol, respectively. To recover the antigenicity, the slides were immersed in EDTA antigen retrieval buffer (pH8.0) and maintained at a sub-boiling temperature for 8 minutes, and then followed by another sub-boiling temperature for 7 minutes. After air-cooling, the slides were washed with PBS thrice. Next, 3% BSA was used to cover the marked tissue and block non-specific binding for 30 minutes. Then, tissue sections were incubated with primary antibodies in PBS containing 3% BSA and 0.1% Tween-20 at 4 °C overnight. After washing thrice with PBS, the secondary antibody was added to cover the target tissue and incubated at room temperature for 50 minutes in the dark. Nucleus were counterstained with DAPI at room temperature for 10 minutes in dark condition. A spontaneous fluorescence quenching reagent was then added and incubated for 5 minutes. After washing with running tap water for 10 minutes, cover slips with anti-fade mounting medium were carefully placed. Images were captured using a fluorescent microscopy (Nikon, ECLIPSE C1) and analyzed using an imaging system (Nikon, DS-U3). Immunofluorescence was performed using the following antibodies: PDGFRB (Abcam, ab69506, 1:50), PDGFRA (Abcam, ab203491, 1:50), PLIN1 (Abcam, ab172907, 1:50), CD9 (Abcam, ab236630, 1:50).

### In vitro lipolysis analysis of adipose explants

The eWAT (~100 mg per mouse) of mice were cut into small pieces (~1 mm^3^) and washed thrice with Opti-MEM (containing 2% fatty acid free BSA). Then, adipose tissues were cultured in Opti-MEM at 37 °C. Culture media were collected at the indicated time points. FFA and glycerol levels in the culture medium were determined using ELISA kits, as described above. At the end of the experiments, adipose explants were collected and total protein was extracted using RIPA buffer (Thermo, 89900).

### qRT-PCR

Total RNA was isolated using TRIzol reagent (Invitrogen, 15596026). For human primary adipocytes, RNA was extracted using a RNeasy Micro Kit (QIGEN, 74106). cDNA was synthesized using a PrimeScriptRT master kit (Takara, RRO36A). Relative gene expression levels were analyzed by quantitative PCR using a Real-Time PCR system (LightCycler 480 II, Roche). The results are presented as relative expression values normalized to RPS18 or TBP. The primers used are listed in the Supplementary Table 2.

### In vitro adipogenesis differentiation

FACS-sorted APC subpopulations from the SVF of lean control, participants with obesity and T2D patients with obesity were cultured with PAM (ScienCell, 7211) in 96 well plates. The medium was changed alternate day until the cells reached approximately 100% confluence. The PAM was then replaced with preadipocyte differentiation medium (ScienCell, 7221). The process of differentiation into mature adipocytes was sustained for 12 days, with fresh differentiation medium changed every 2-3 days. Adipogenesis was assessed by staining lipid droplets with Bodipy 493/503 (Invitrogen, D3922) and nucleus with Hoechst 33342 (Thermo Fisher, 62249). Photographs were captured using a Thunder Imager (Leica). Image J software was used to calculate adipogenic index. At least 50 pictures per group were analyzed.

Isolated mouse CD9^−^ APCs were plated on 96-well plates and cultured in DMEM/F12 containing 10% FBS. After incubated for 48 hours to facilitate attachment, a full adipogenic cocktail [20 nM insulin, 1 nM T3, 1 μM dexamethasone (Sigma, D4902), 0.5 μM isobutylmethylxanthine (Sigma, I5879) and 125 nM indomethacin (Sigma, I7378)] was added. Cells was incubated for 2 days and then transferred to adipogenic maintenance medium (20 nM insulin, 1 nM T3). Maintenance medium was changed every 2 days. The cells were analyzed after 8 days of differentiation.

### Statistical analysis

All statistical analyses were performed using the SPSS (version 22.0, SPSS Inc., USA) or GraphPad Prism 9 software. Differences in continuous variables between the two groups were determined using an unpaired two-tailed Students’ *t* test. Group differences were compared using one-way ANOVA variance for normally distributed variables, followed by Tukey’s multiple comparison test or Dunnett’s multiple comparisons test. Spearman’s bivariate correlation tests were conducted to investigate these associations. *P* values of 0.05 or less were considered statistically significant.

### Reporting summary

Further information on research design is available in the Nature Portfolio Reporting Summary linked to this article.

### Supplementary information

Supplementary information

Peer Review File

Reporting Summary

### Source data

Source Data

## Data Availability

The reference files for human genome (GRCh38 version) and mouse genome (mm10 version) were built from https://www.10xgenomics.com/support/software/cell-ranger/latest/tutorials/cr-tutorial-mr. The processed public scRNA datasets were download from Gene Expression Omnibus: GEO, https://www.ncbi.nlm.nih.gov/geo/ including GSE128889 (Merrick et al. mouse SAT), GSE176067 (Emont et al. human WAT) and ArrayExpress database (www.ebi.ac.uk/arrayexpress) with the accession numbers E-MTAB-6677 (Schwalie et al. mouse SAT). Raw data of scRNA-seq in this study are uploaded in the Genome Sequence Archive for Human with accession number HRA002549 that are publicly accessible at https://ngdc.cncb.ac.cn/gsa-human/browse/HRA002549. Source data are provided as a Source Data file. All other information is available within the manuscript, supplementary information file, or upon request to the corresponding author. All original code has been deposited at GitHub and is available at https://github.com/dyanhua/APCs_scRNA-Seq. Any additional information required to reanalyze the data reported in this paper is available from the lead contact upon request. Source data are provided with this paper.
