# Supplementary material for: Single-cell analysis reveals a subpopulation of adipose progenitor cells that impairs glucose homeostasis
Source: Nat Commun. 2024 Jun 6;15:4827. doi: 10.1038/s41467-024-48914-w (PMC11156882; doi:10.1038/s41467-024-48914-w)
Supplement: Supplementary file 1 — Supplementary information [file 41467_2024_48914_MOESM1_ESM.pdf]

Supplementary Information

Wang *et al.*

Single-cell analysis reveals a subpopulation of adipose progenitor cells that impairs glucose homeostasis

This file includes Supplementary Tables 1-2 and Supplementary Figures 1-9.

Correspondence and reprint requests

Yan Bi, Department of Endocrinology, Drum Tower Hospital affiliated to Nanjing University Medical School, Branch of National Clinical Research Centre for Metabolic Diseases, 321 Zhongshan Road, 210008, Nanjing, China, E-mail: biyan@nju.edu.cn.

Lei Shen, Shanghai Institute of Immunology, Shanghai Jiao Tong University School of Medicine, 227 Chongqing South Road, 200025, Shanghai, China, E-mail: lshen@shsmu.edu.cn.

Supplementary Tables

Supplementary Table 1 Demographics of subjects enrolled for single-cell RNA sequencing

	Lean	OB	T2D
Number of participants	2	3	3
Female sex (%)	100	33.3	33.3
BMI (kg m^{-2})	22.2±1.8	37.3±5.5	45.1±10.7

OB, participants with obesity; T2D, patients with type 2 diabetes and obesity; BMI, body mass index.

Supplementary Table 2 Primers used in this study

For human studies:

Number	Gene	Forward (5'-3')	Reverse (5'-3')
1	*ZFAND5*	GCTAGTGGTTCCAACAGTCCT	TCGGGGTAGTTATTTTGTCCTCT
2	*KLF4*	CCCACATGAAGCGACTTCCC	CAGGTCCAGGAGATCGTTGAA
3	*ZNF331*	TTCGCCGACGTAGCCATAGA	CGTCCCAGTACAGGTCCCT
4	*ADAMTS1*	ACTGGAAGCATAAGAAAGAAGCG	AATTCTGCCATCGACTGGTCT
5	*SEMA4A*	TGGATGGGATGCTCTATTCTGG	GCGGAGGAAGTTGTCGGTC
6	*MMP19*	GCTTCCTACTCCCCATGACAG	CCCATATTGTGACAGGTAGTCCA
7	*POSTN*	CTCATAGTCGTATCAGGGGTCG	ACACAGTCGTTTTCTGTCCAC
8	*MYC*	GGCTCCTGGCAAAAGGTCA	CTGCGTAGTTGTGCTGATGT
9	*NR4A1*	ATGCCCTGTATCCAAGCCC	GTGTAGCCGTCCATGAAGGT
10	*CSRNP1*	CCCCTGTCTATCCTGAAGCG	CCATACCCAGAGTACAGCCA
11	*NNMT*	ATATTCTGCCTAGACGGTGTGA	TCAGTGACGACGATCTCCTTAAA
12	*TIPARP*	AGAACGAGTGGTTCCAATCCA	TGGGTGCAAAAGATCAGTCTG
13	*INTS6*	AGCCGCCCTATGCTATCAAG	CAAGAGTCGTAAGTCCTTCAGC
14	*CHSY1*	CTGGGCACCACGGAAGAAAT	GGCACCATTCTCCGAAGCA
15	*PGAP1*	CTTCGGCTTCGAGGAGAATAAG	GGGATAGCGTTTTGCCAGTTT
16	*GADD45B*	TACGAGTCGGCCAAGTTGATG	GGATGAGCGTGAAGTGGATTT
17	*SERTAD1*	CAAGGGTCTGAAGCGGAAAC	GCTTGAGCACTGAGAGGTCAAA
18	*ANK2*	ACCTGCAATCAGAATGGACTCA	TGCAATGTGAAGAGCGGTATT
19	*RGS2*	AAGATTGGAAGACCCGTTTGAG	GCAAGACCATATTTGCTGGCT
20	*BGN*	CAGTGGCTTTGAACCTGGAG	GGGAGGTCTTTGGGGATGC
21	*CHEMERIN*	AGAAACCCGAGTGCAAAGTCA	AGAACTTGGGTCTCTATGGGG
22	*MCP1*	CAGCCAGATGCAATCAATGCC	TGGAATCCTGAACCCACTTCT
23	*ANGPTL8*	AGAAGGTGCTACGGGACAG	AGCGTGAGCCTTTAAGACCTC
24	*ANGPTL2*	GAACCGAGTGCATAAGCAGGA	GTGACCCGCGAGTTCATGTT
25	*ANG1*	AGCGCCGAAGTCCAGAAAAC	TACTCTCACGACAGTTGCCAT
26	*ANG2*	AACTTTCGGAAGAGCATGGAC	CGAGTCATCGTATTCGAGCGG
27	*LEPTIN*	TGCCTTCCAGAAACGTGATCC	CTCTGTGGAGTAGCCTGAAGC
28	*CFD*	GACACCATCGACCACGACC	GCCACGTCGCAGAGAGTTC
29	*RBP4*	AGGAGAACTTCGACAAGGCTC	GAGAACTCCGCGACGATGTT
30	*CTRP3*	TCTCCACAAACCGGAGGACTA	CCTTGGTAGCCTCGAAAGC
31	*IL1B*	ATGATGGCTTATTACAGTGGCAA	GTCGGAGATTCGTAGCTGGA
32	*TNFSF10*	TGCGTGCTGATCGTGATCTTC	GCTCGTTGGTAAAGTACACGTA
33	*APELIN*	GTCTCCTCCATAGATTGGTCTGC	GGAATCATCCAAACTACAGCCAG
34	*ADIPOQ*	TGGTGAGAAGGGTGAGAAAGG	CTCCAATCCCACACTGAATGC
35	*SERPINE1*	ACCGCAACGTGGTTTTCTCA	TTGAATCCCATAGCTGCTTGAAT
36	*NAMPT*	CGGCAGAAGCCGAGTTCAA	GCTTGTGTTGGGTGGATATTGTT
37	*FABP4*	ACTGGGCCAGGAATTTGACG	CTCGTGGAAGTGACGCCTT
38	*BMP4*	ATGATTCCTGGTAACCGAATGC	CCCCGTCTCAGGTATCAAACT
39	*SERPINF1*	TTCAAAGTCCCCGTGAACAAG	GAGAGCCCGGTGAATGATGG
40	*NRG4*	ATGCCAACAGATCACGAAGAG	AATGGGCTGGGAATAGTAGGT
41	*COL1A1*	GAGGGCCAAGACGAAGACATC	CAGATCACGTCATCGCACAAC

42	*COL3A1*	GGAGCTGGCTACTTCTCGC	GGGAACATCCTCCTTCAACAG
43	*COL6A1*	ACAGTGACGAGGTGGAGATCA	GATAGCGCAGTCGGTGTAGG
44	*TIMP1*	CTTCTGCAATTCCGACCTCGT	ACGCTGGTATAAGGTGGTCTG
45	*FN1*	CGGTGGCTGTCAGTCAAAG	AAACCTCGGCTTCCTCCATAA
46	*LOX*	CGGCGGAGGAAAACTGTCT	TCGGCTGGGTAAGAAATCTGA
47	*TGFB1*	GGCCAGATCCTGTCCAAGC	GTGGGTTTCCACCATTAGCAC
48	*RPS18*	GCGGCGGAAAATAGCCTTTG	GATCACACGTTCCACCTCATC
49	*TBP*	TTTGCTGCGGTAATCATGAGG	GCTGGAAAACCCAACTTCTGT

For animal studies:

Number	Gene	Forward (5’-3’)	Reverse (5’-3’)
1	*Il1b*	GAAATGCCACCTTTTGACAGTG	TGGATGCTCTCATCAGGACAG
2	*Il6*	CTGCAAGAGACTTCCATCCAG	AGTGGTATAGACAGGTCTGTTGG
3	*Tnfα*	CAGGCGGTGCCTATGTCTC	CGATCACCCCGAAGTTCAGTAG
4	*Rps18*	TTCCAGCACATTTTGCGAGTA	CACGCCCTTAATGGCAGTGAT

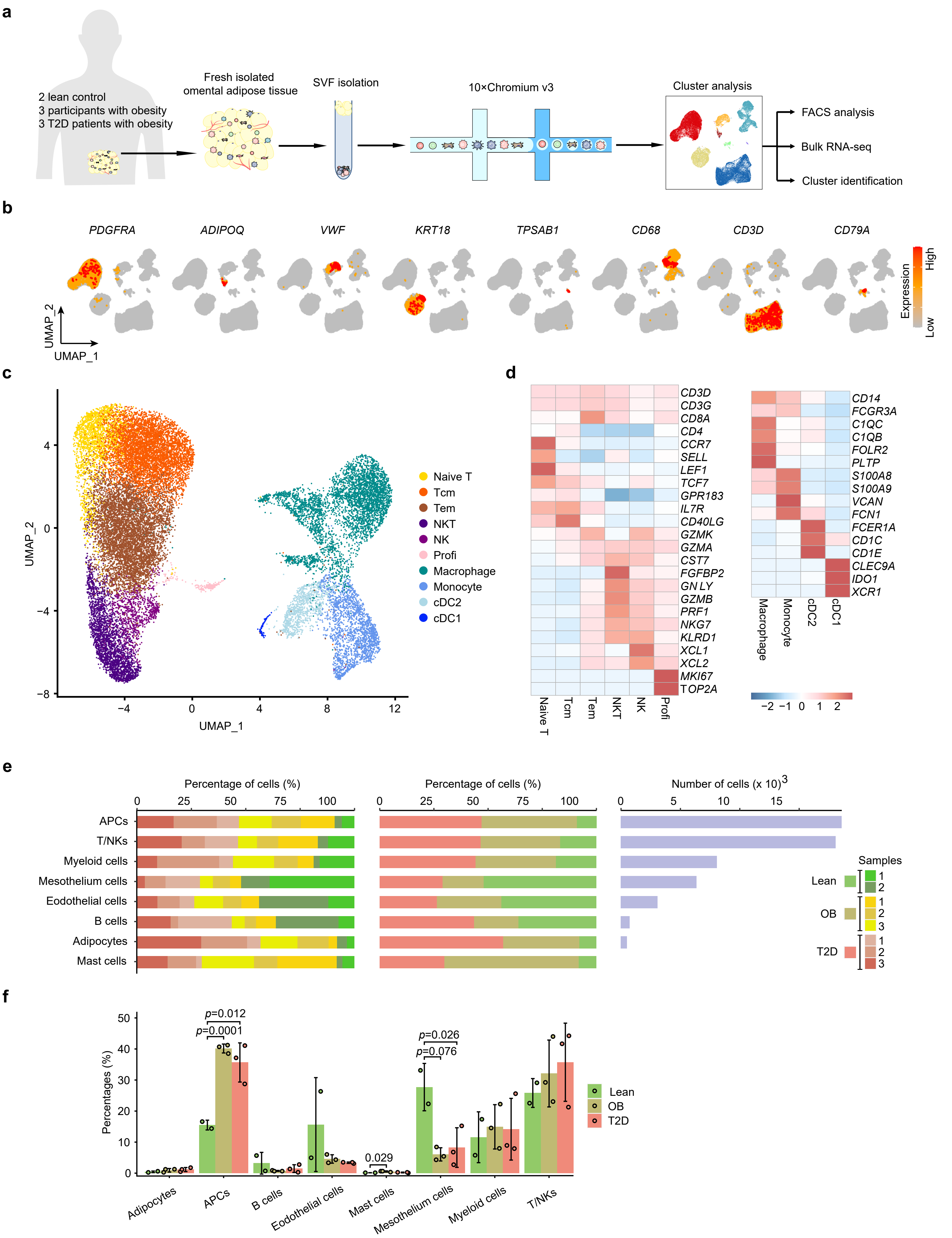

a
2 lean control
3 participants with obesity
3 T2D patients with obesity
Fresh isolated omental adipose tissue
SVF isolation
10×Chromium v3
Cluster analysis
FACS analysis
Bulk RNA-seq
Cluster identification
b
PDGFRA
ADIPOQ
VWF
KRT18
TPSAB1
CD68
CD3D
CD79A
Expression
High
Low
UMAP_2
UMAP_1
c
Naive T
Tcm
Tem
NKT
NK
Profi
Macrophage
Monocyte
cDC2
cDC1
d
CD3D
CD3G
CD8A
CD4
CCR7
SELL
LEF1
TCF7
GPR183
IL7R
CD40LG
GZMK
GZMA
CST7
FGFBP2
GNLY
GZMB
PRF1
NKG7
KLRD1
XCL1
XCL2
MKI67
TOP2A
CD14
FCGR3A
C1QC
C1QB
FOLR2
PLTP
S100A8
S100A9
VCAN
FCN1
FCER1A
CD1C
CD1E
CLEC9A
IDO1
XCR1
e
Percentage of cells (%)
Number of cells (x 10)3
APCs
T/NKs
Myeloid cells
Mesothelium cells
Eodothelial cells
B cells
Adipocytes
Mast cells
Samples
Lean
OB
T2D
f
Percentages (%)
p=0.012
p=0.0001
p=0.026
p=0.076
0.029
Lean
OB
T2D
Adipocytes
APCs
B cells
Eodothelial cells
Mast cells
Mesothelium cells
Myeloid cells
T/NKs

Supplementary Fig. 1 A single-cell atlas of human adipose SVF. **a** Schematic of workflow for scRNA-seq. Periumbilical adipose tissues were collected from 2 lean control subjects, 3 participants with obesity and 3 obese newly-diagnosed type 2 diabetic patients with obesity. SVF samples were isolated and processed into single-cell suspension. scRNA-seq was performed using 10×Genomics. **b** UMAP plot for the marker genes in eight cell populations of human adipose SVF. **c** UMAP plot for unsupervised clustering of immune cells in human adipose SVF. Each cluster was shown in different color. Tcm, central memory T cells; Tem, effective memory T cells; Profi, proliferation cells; cDC, conventional dendritic cell. **d** Heatmap of marker genes defining the clusters indicated in (**c**). **e**,**f** Proportion of each cluster showing in bar plots in different donors (**e**) and groups (**f**). Lean, lean individuals; OB, participants with obesity; T2D, patients with type 2 diabetes and obesity. Data are means ± SD. Data in **f** were analyzed using Wilcoxon rank-sum test. Source data are provided as a Source data file.

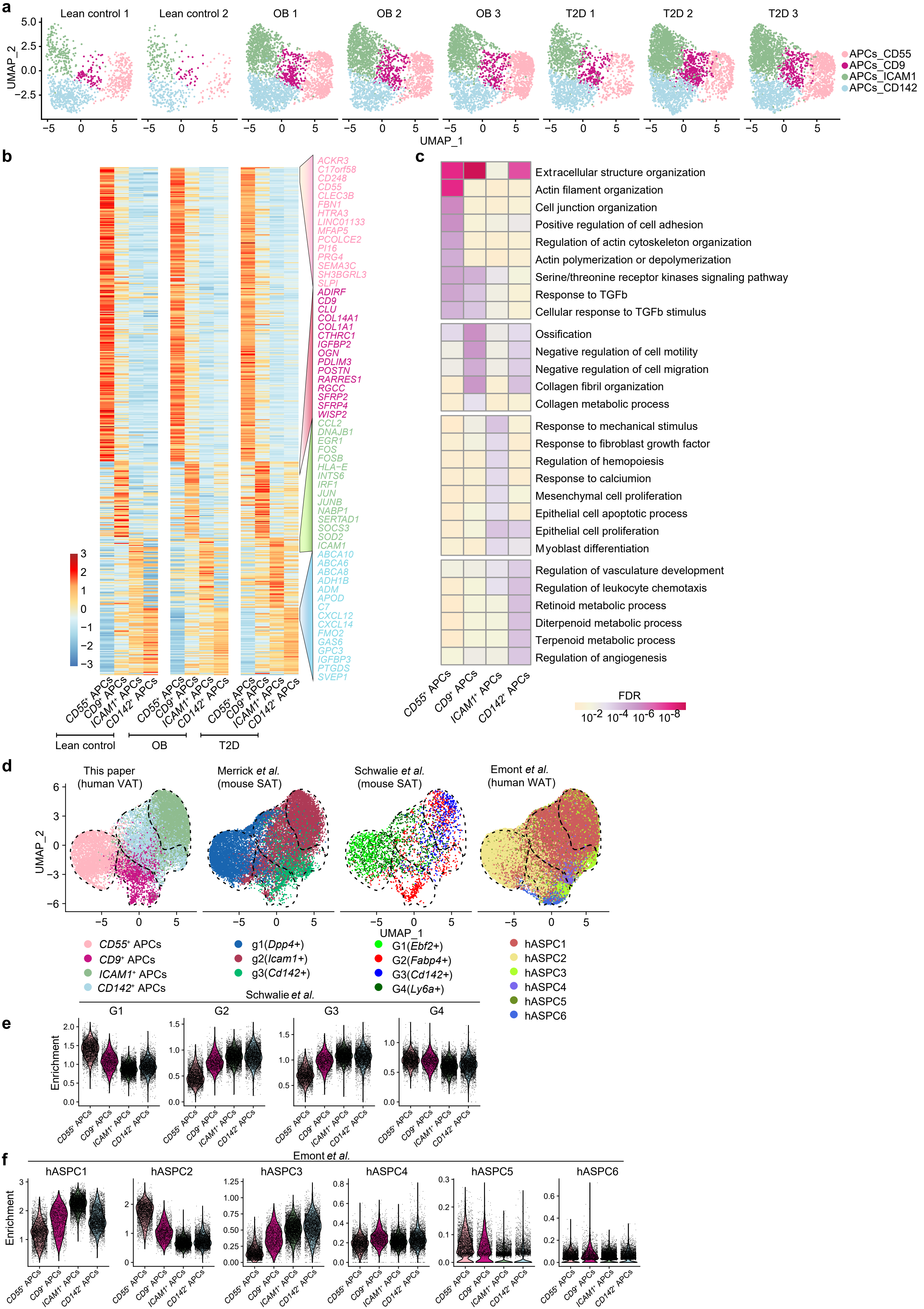

a
Lean control 1
Lean control 2
OB 1
OB 2
OB 3
T2D 1
T2D 2
T2D 3
UMAP_2
UMAP_1
APCs_CD55
APCs_CD9
APCs_ICAM1
APCs_CD142
b
ACKR3
C17orf58
CD248
CD55
CLEC3B
FBN1
HTRA3
LINC01133
MFAP5
PCOLCE2
PI16
PRG4
SEMA3C
SH3BGRL3
SLPI
ADIRF
CD9
CLU
COL14A1
COL1A1
CTHRC1
IGFBP2
OGN
PDLIM3
POSTN
RARRES1
RGCC
SFRP2
SFRP4
WISP2
CCL2
DNAJB1
EGR1
FOS
FOSB
HLA−E
INTS6
IRF1
JUN
JUNB
NABP1
SERTAD1
SOCS3
SOD2
ICAM1
ABCA10
ABCA6
ABCA8
ADH1B
ADM
APOD
C7
CXCL12
CXCL14
FMO2
GAS6
GPC3
IGFBP3
PTGDS
SVEP1
CD55+ APCs
CD9+ APCs
ICAM1+ APCs
CD142+ APCs
Lean control
OB
T2D
c
Extracellular structure organization
Actin filament organization
Cell junction organization
Positive regulation of cell adhesion
Regulation of actin cytoskeleton organization
Actin polymerization or depolymerization
Serine/threonine receptor kinases signaling pathway
Response to TGFb
Cellular response to TGFb stimulus
Ossification
Negative regulation of cell motility
Negative regulation of cell migration
Collagen fibril organization
Collagen metabolic process
Response to mechanical stimulus
Response to fibroblast growth factor
Regulation of hemopoiesis
Response to calciumion
Mesenchymal cell proliferation
Epithelial cell apoptotic process
Epithelial cell proliferation
Myoblast differentiation
Regulation of vasculature development
Regulation of leukocyte chemotaxis
Retinoid metabolic process
Diterpenoid metabolic process
Terpenoid metabolic process
Regulation of angiogenesis
CD55+ APCs
CD9+ APCs
ICAM1+ APCs
CD142+ APCs
FDR
10-2 10-4 10-6 10-8
d
This paper (human VAT)
Merrick et al. (mouse SAT)
Schwalie et al. (mouse SAT)
Emont et al. (human WAT)
UMAP_2
UMAP_1
CD55+ APCs
CD9+ APCs
ICAM1+ APCs
CD142+ APCs
g1(Dpp4+)
g2(Icam1+)
g3(Cd142+)
G1(Ebf2+)
G2(Fabp4+)
G3(Cd142+)
G4(Ly6a+)
hASPC1
hASPC2
hASPC3
hASPC4
hASPC5
hASPC6
e
Schwalie et al.
G1
G2
G3
G4
Enrichment
f
Emont et al.
hASPC1
hASPC2
hASPC3
hASPC4
hASPC5
hASPC6
Enrichment

Supplementary Fig. 2 This figure is related to Figure 1. **a** UMAP plot for unsupervised clustering of APC populations in human adipose SVF from each donor. OB, participants with obesity; T2D, patients with type 2 diabetes and obesity. **b** Heatmap of expression of marker genes defining four APC populations. OB, participants with obesity; T2D, patients with type 2 diabetes and obesity. **c** GO analysis of marker genes of four APC subpopulations. **d** Integration of APC from this paper with APC from other groups. VAT, visceral adipose tissue; SAT, subcutaneous adipose tissue; WAT, white adipose tissue. **e**,**f** Violin plots representing enrichment scores of each APC-associated markers based on previous studies across the four APCs from this paper. G1, *Ebf2*+ subpopulation; G2, *Fabp4*+ subpopulation; G3, *Cd142*+ subpopulation; G4, *Ly6a*+ subpopulation. The scores were calculated by the average expression of each gene signature.

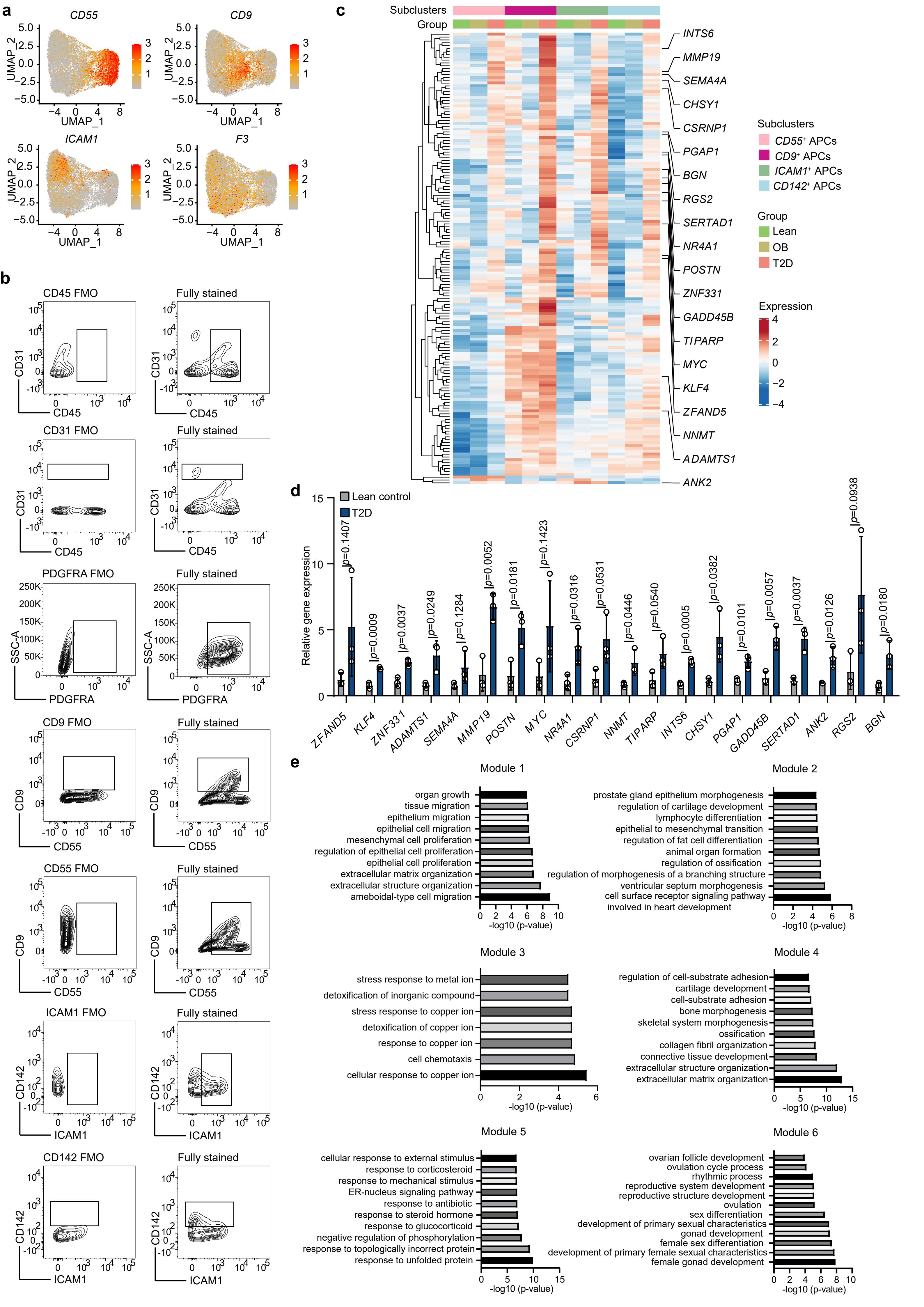
a
CD55
CD9
ICAM1
F3
UMAP_1
UMAP_2
b
CD45 FMO
Fully stained
CD31
CD45
CD31 FMO
PDGFRA FMO
SSC-A
PDGFRA
CD9 FMO
CD9
CD55
CD55 FMO
ICAM1 FMO
CD142
ICAM1
CD142 FMO
c
Subclusters
Group
INTS6
MMP19
SEMA4A
CHSY1
CSRNP1
PGAP1
BGN
RGS2
SERTAD1
NR4A1
POSTN
ZNF331
GADD45B
TIPARP
MYC
KLF4
ZFAND5
NNMT
ADAMTS1
ANK2
Subclusters
CD55⁺ APCs
CD9⁺ APCs
ICAM1⁺ APCs
CD142⁺ APCs
Group
Lean
OB
T2D
Expression
d
Lean control
T2D
Relative gene expression
p=0.1407
p=0.0009
p=0.0037
p=0.0249
p=0.1284
p=0.0052
p=0.0181
p=0.1423
p=0.0316
p=0.0531
p=0.0446
p=0.0540
p=0.0005
p=0.0382
p=0.0101
p=0.0057
p=0.0037
p=0.0126
p=0.0938
p=0.0180
ZFAND5
KLF4
ZNF331
ADAMTS1
SEMA4A
MMP19
POSTN
MYC
NR4A1
CSRNP1
NNMT
TIPARP
INTS6
CHSY1
PGAP1
GADD45B
SERTAD1
ANK2
RGS2
BGN
e
Module 1
organ growth
tissue migration
epithelium migration
epithelial cell migration
mesenchymal cell proliferation
regulation of epithelial cell proliferation
epithelial cell proliferation
extracellular matrix organization
extracellular structure organization
ameboidal-type cell migration
-log10 (p-value)
Module 2
prostate gland epithelium morphogenesis
regulation of cartilage development
lymphocyte differentiation
epithelial to mesenchymal transition
regulation of fat cell differentiation
animal organ formation
regulation of ossification
regulation of morphogenesis of a branching structure
ventricular septum morphogenesis
cell surface receptor signaling pathway involved in heart development
-log10 (p-value)
Module 3
stress response to metal ion
detoxification of inorganic compound
stress response to copper ion
detoxification of copper ion
response to copper ion
cell chemotaxis
cellular response to copper ion
-log10 (p-value)
Module 4
regulation of cell-substrate adhesion
cartilage development
cell-substrate adhesion
bone morphogenesis
skeletal system morphogenesis
ossification
collagen fibril organization
connective tissue development
extracellular structure organization
extracellular matrix organization
-log10 (p-value)
Module 5
cellular response to external stimulus
response to corticosteroid
response to mechanical stimulus
ER-nucleus signaling pathway
response to antibiotic
response to steroid hormone
response to glucocorticoid
negative regulation of phosphorylation
response to topologically incorrect protein
response to unfolded protein
-log10 (p-value)
Module 6
ovarian follicle development
ovulation cycle process
rhythmic process
reproductive system development
reproductive structure development
ovulation
sex differentiation
development of primary sexual characteristics
gonad development
female sex differentiation
development of primary female sexual characteristics
female gonad development
-log10 (p-value)

Supplementary Fig. 3 This figure is related to Figure 2. **a** UMAP showing the expression level and distribution of representative marker genes. F3 (CD142). **b** FMO controls of all markers used in APC subpopulation identification. **c** A heatmap of expression of genes in module 3 and 4. Lean, lean individuals; OB, participants with obesity; T2D, patients with type 2 diabetes and obesity. **d** mRNA levels of module genes in $CD9^{+}CD55^{low}$ APCs from T2D patients (n=3) compared to that of lean control subjects (n=3), assessed by qPCR assay. **e** Representative gene ontology (GO) terms enriched in different gene modules. Data are means ± SD. Two-tailed unpaired Student's *t* test was used to evaluate statistical significance. Source data are provided as a Source data file.

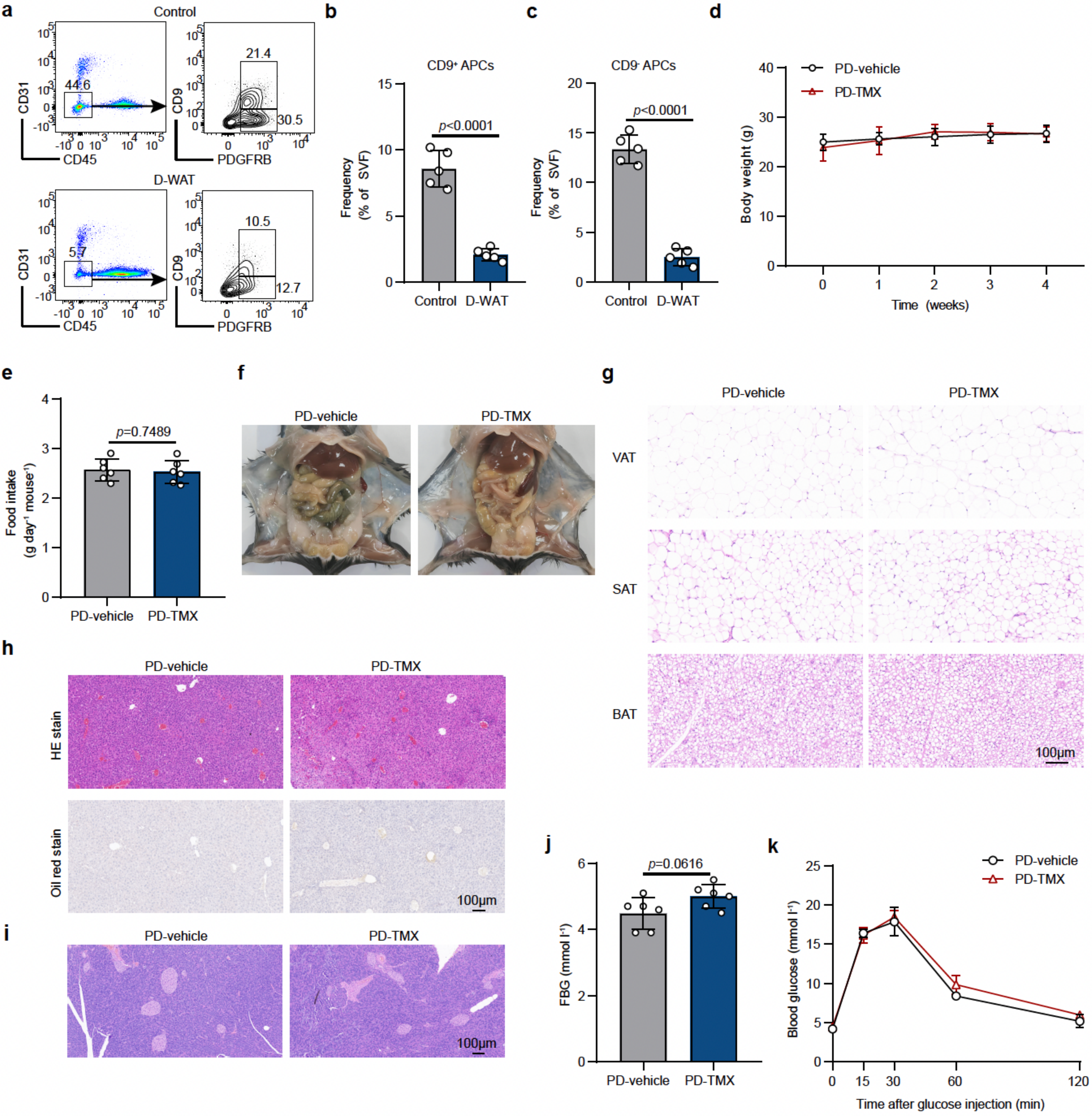
a
Control
CD31
CD45
44.6
CD9
PDGFRB
21.4
30.5
D-WAT
5.7
10.5
12.7
b
CD9+ APCs
Frequency (% of SVF)
p<0.0001
Control
D-WAT
c
CD9- APCs
Frequency (% of SVF)
p<0.0001
Control
D-WAT
d
PD-vehicle
PD-TMX
Body weight (g)
Time (weeks)
e
Food intake (g day-1 mouse-1)
p=0.7489
PD-vehicle
PD-TMX
f
PD-vehicle
PD-TMX
g
PD-vehicle
PD-TMX
VAT
SAT
BAT
100μm
h
PD-vehicle
PD-TMX
HE stain
Oil red stain
100μm
i
PD-vehicle
PD-TMX
100μm
j
FBG (mmol l-1)
p=0.0616
PD-vehicle
PD-TMX
k
PD-vehicle
PD-TMX
Blood glucose (mmol l-1)
Time after glucose injection (min)

Supplementary Fig. 4 This figure is related to Figure 3. D-WAT (4 mg per mouse) was injected *in situ* into the eWAT of 8-weeks old C57BL/6 mice (n=5 per group). Three days after D-WAT treatment, the frequencies of APC subpopulations in the eWAT were detected by flow cytometry (**a**) and quantified (**b,c**). For **d-k**, eight-weeks old PD mice were treated with tamoxifen or vehicle, after which metabolic parameters were determined. **d** Body weight (n=6 per group). PD, *Pdgfra*-CreERT2;DTA$^{flox/-}$ mice; TMX, tamoxifen. **e** Daily food intake (n=6 per group). Four weeks after tamoxifen treatment, GTT assay was performed and metabolic tissues were collected for further analysis. **f** Ventral aspect of control and tamoxifen treated PD mice. **g** H&E staining of adipose tissues of control and tamoxifen treated PD mice. VAT, visceral adipose tissue; SAT, subcutaneous adipose tissue; BAT, brown adipose tissue. **h** H&E and oil red staining of liver tissue. **i** H&E staining of pancreatic tissue. **j** Fasting blood glucose levels (n=6 per group). **k** Glucose tolerance test (n=5 per group). Data are means ± SD. For statistical analysis, two-tailed unpaired Student's *t* test was used. Source data are provided as a Source data file.

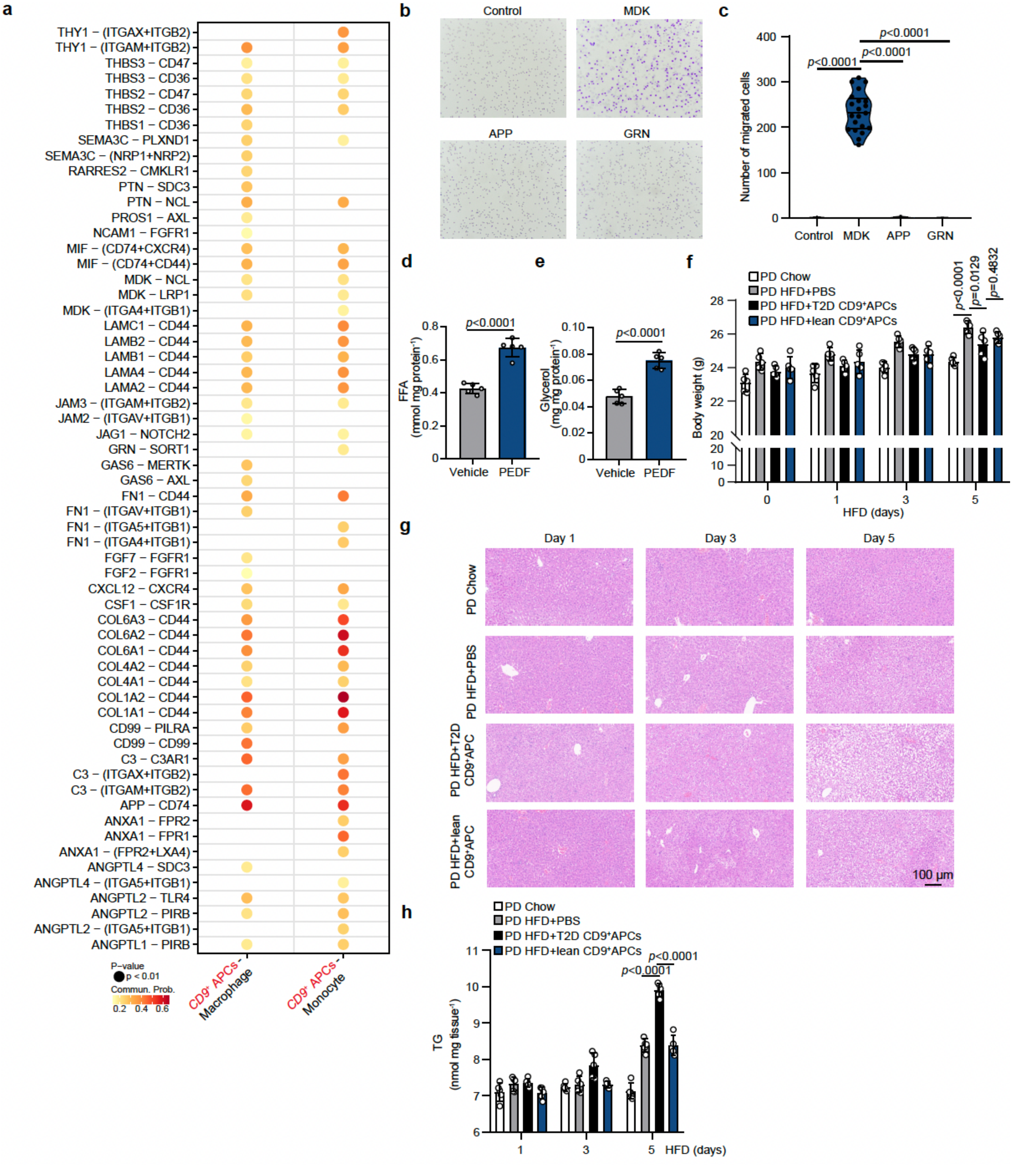
a
THY1 − (ITGAX+ITGB2)
THY1 − (ITGAM+ITGB2)
THBS3 − CD47
THBS3 − CD36
THBS2 − CD47
THBS2 − CD36
THBS1 − CD36
SEMA3C − PLXND1
SEMA3C − (NRP1+NRP2)
RARRES2 − CMKLR1
PTN − SDC3
PTN − NCL
PROS1 − AXL
NCAM1 − FGFR1
MIF − (CD74+CXCR4)
MIF − (CD74+CD44)
MDK − NCL
MDK − LRP1
MDK − (ITGA4+ITGB1)
LAMC1 − CD44
LAMB2 − CD44
LAMB1 − CD44
LAMA4 − CD44
LAMA2 − CD44
JAM3 − (ITGAM+ITGB2)
JAM2 − (ITGAV+ITGB1)
JAG1 − NOTCH2
GRN − SORT1
GAS6 − MERTK
GAS6 − AXL
FN1 − CD44
FN1 − (ITGAV+ITGB1)
FN1 − (ITGA5+ITGB1)
FN1 − (ITGA4+ITGB1)
FGF7 − FGFR1
FGF2 − FGFR1
CXCL12 − CXCR4
CSF1 − CSF1R
COL6A3 − CD44
COL6A2 − CD44
COL6A1 − CD44
COL4A2 − CD44
COL4A1 − CD44
COL1A2 − CD44
COL1A1 − CD44
CD99 − PILRA
CD99 − CD99
C3 − C3AR1
C3 − (ITGAX+ITGB2)
C3 − (ITGAM+ITGB2)
APP − CD74
ANXA1 − FPR2
ANXA1 − FPR1
ANXA1 − (FPR2+LXA4)
ANGPTL4 − SDC3
ANGPTL4 − (ITGA5+ITGB1)
ANGPTL2 − TLR4
ANGPTL2 − PIRB
ANGPTL2 − (ITGA5+ITGB1)
ANGPTL1 − PIRB
P−value
p < 0.01
Commun. Prob.
0.2 0.4 0.6
CD9+ APCs - Macrophage
CD9+ APCs - Monocyte
b
Control
MDK
APP
GRN
c
Number of migrated cells
400
300
200
100
0
Control MDK APP GRN
p<0.0001
p<0.0001
p<0.0001
d
FFA (mmol mg protein-1)
0.8
0.6
0.4
0.2
0
Vehicle PEDF
p<0.0001
e
Glycerol (mg mg protein-1)
0.10
0.08
0.06
0.04
0.02
0
Vehicle PEDF
p<0.0001
f
PD Chow
PD HFD+PBS
PD HFD+T2D CD9+APCs
PD HFD+lean CD9+APCs
Body weight (g)
28
26
24
22
20
20
10
0
0 1 3 5
HFD (days)
p<0.0001
p=0.0129
p=0.4832
g
Day 1
Day 3
Day 5
PD Chow
PD HFD+PBS
PD HFD+T2D CD9+APC
PD HFD+lean CD9+APC
100 µm
h
PD Chow
PD HFD+PBS
PD HFD+T2D CD9+APCs
PD HFD+lean CD9+APCs
TG (nmol mg tissue-1)
11
10
9
8
7
6
1 3 5 HFD (days)
p<0.0001
p<0.0001

Supplementary Fig. 5 $CD9^{+}CD55^{low}$ APCs triggered a series of metabolic impairments. **a** The significant ligand-receptor pairs involved in the interaction between *CD9*$^{+}$ APCs and macrophages or monocytes. **b**,**c** Human blood monocytes were isolated and treated with recombinant human MDK (0.5 μg ml^{-1}), APP (0.5 μg ml^{-1}) and GRN (0.5 μg ml^{-1}) protein for 24 hours, migrated monocytes were detected (**b**) and quantified (**c**). Data are representative of three independent experiments. **d**,**e** Human primary adipocytes were treated with PEDF (100 nmol l^{-1}) for 24 hours, levels of FFA (**d**) and glycerol (**e**) in conditioned medium were determined. FFA, free fatty acid. Panels **f-h** are related to Figure 5. **f** Body weight of mice in four groups at the indicated timepoint (n=5 per group). **g**,**h** Lipid deposition was determined in liver of receipt mice receiving human APCs transfer. **g** Representative microscopic images with H&E staining in liver. **h** Hepatic triglyceride (TG) levels (n=5 per group). Data are means ± SD. For statistical analysis, **d,e** Two-tailed unpaired Student's *t* test; for **c,f,h,** One-way ANOVA was used followed by Tukey's multiple comparison test. Source data are provided as a Source data file.

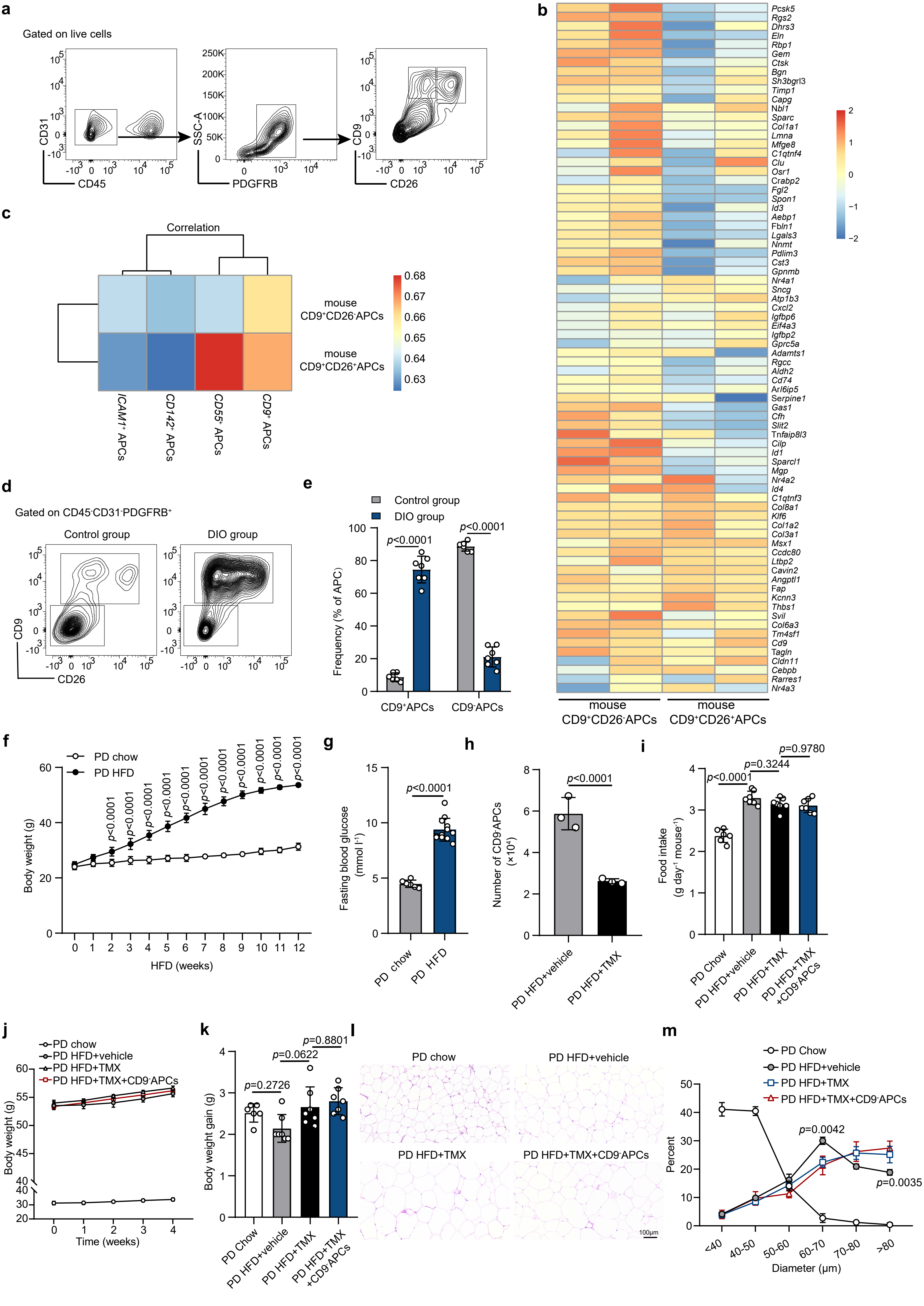
a
Gated on live cells
CD31
CD45
SSC-A
PDGFRB
CD9
CD26
b
Pcsk5
Rgs2
Dhrs3
Eln
Rbp1
Gem
Ctsk
Bgn
Sh3bgrl3
Timp1
Capg
Nbl1
Sparc
Col1a1
Lmna
Mfge8
C1qtnf4
Clu
Osr1
Crabp2
Fgl2
Spon1
Id3
Aebp1
Fbln1
Lgals3
Nnmt
Pdlim3
Cst3
Gpnmb
Nr4a1
Sncg
Atp1b3
Cxcl2
Igfbp6
Eif4a3
Igfbp2
Gprc5a
Adamts1
Rgcc
Aldh2
Cd74
Arl6ip5
Serpine1
Gas1
Cfh
Slit2
Tnfaip8l3
Cilp
Id1
Sparcl1
Mgp
Nr4a2
Id4
C1qtnf3
Col8a1
Klf6
Col1a2
Col3a1
Msx1
Ccdc80
Ltbp2
Cavin2
Angptl1
Fap
Kcnn3
Thbs1
Svil
Col6a3
Tm4sf1
Cd9
Tagln
Cldn11
Cebpb
Rarres1
Nr4a3
mouse CD9+CD26-APCs
mouse CD9+CD26+APCs
c
Correlation
mouse CD9+CD26-APCs
mouse CD9+CD26+APCs
ICAM1+ APCs
CD142+ APCs
CD55+ APCs
CD9+ APCs
d
Gated on CD45-CD31-PDGFRB+
Control group
DIO group
CD9
CD26
e
Control group
DIO group
Frequency (% of APC)
p<0.0001
p<0.0001
CD9+APCs
CD9-APCs
f
PD chow
PD HFD
Body weight (g)
HFD (weeks)
p<0.0001
g
Fasting blood glucose (mmol l-1)
p<0.0001
PD chow
PD HFD
h
Number of CD9-APCs (×10^4)
p<0.0001
PD HFD+vehicle
PD HFD+TMX
i
Food intake (g day-1 mouse-1)
p<0.0001
p=0.3244
p=0.9780
PD Chow
PD HFD+vehicle
PD HFD+TMX
PD HFD+TMX +CD9-APCs
j
PD chow
PD HFD+vehicle
PD HFD+TMX
PD HFD+TMX+CD9-APCs
Body weight (g)
Time (weeks)
k
Body weight gain (g)
p=0.2726
p=0.0622
p=0.8801
PD Chow
PD HFD+vehicle
PD HFD+TMX
PD HFD+TMX +CD9-APCs
l
PD chow
PD HFD+vehicle
PD HFD+TMX
PD HFD+TMX+CD9-APCs
100μm
m
PD Chow
PD HFD+vehicle
PD HFD+TMX
PD HFD+TMX+CD9-APCs
Percent
p=0.0042
p=0.0035
Diameter (μm)
<40
40-50
50-60
60-70
70-80
>80

Supplementary Fig. 6 This figure is related to Figure 6. **a-c** Mouse APCs was isolated and performed bulk RNA-seq. **a** Sorting strategy of mouse APCs. **b** A heatmap showing the expression patterns of marker genes of human *CD9*$^{+}$ APCs defined by our scRNA-seq data in FACS-sorted mouse CD9^{+} APCs. **c** Person correlation analysis between gene expression in identified human APC subsets by scRNA-seq and gene expression in mouse CD9^{+} APCs by bulk RNA-seq. **d**,**e** APC subpopulations in eWAT of DIO (n=7) and control mice (n=6) were determined by flow cytometry (**d**) and quantified (**e**). DIO, diet induced obesity. Eight-weeks old PD mice were fed a HFD for 3 months and body weight was monitored weekly (chow, n= 6; HFD, n=21) (**f**). **g** Fasting blood glucose level of PD mice fed with HFD or chow diet for 3 months (chow, n= 6; HFD, n=12). **h** After tamoxifen or vehicle treatment, the eWAT of each tamoxifen or vehicle treated obese PD mouse was collected and absolute number of CD9^{-}APCs in the eWAT was determined by flow cytometry (n=3 per group). TMX, tamoxifen. **i** Food intake (PD chow, n=6; all other groups, n=7). **j** Body weight (PD chow, n=6; all other groups, n=7). **k** Body weight gain (PD chow, n=6; all other groups, n=7). **l** H&E staining of the eWAT of mice in four groups. **m.** Quantification of adipocyte diameter (n=3 per group). Data are means ± SD. For statistical analysis, **e,i-k,m,** One-way ANOVA was used followed by Tukey's multiple comparison test; for **f-h,** Two-tailed unpaired Student's *t* test. Source data are provided as a Source data file.

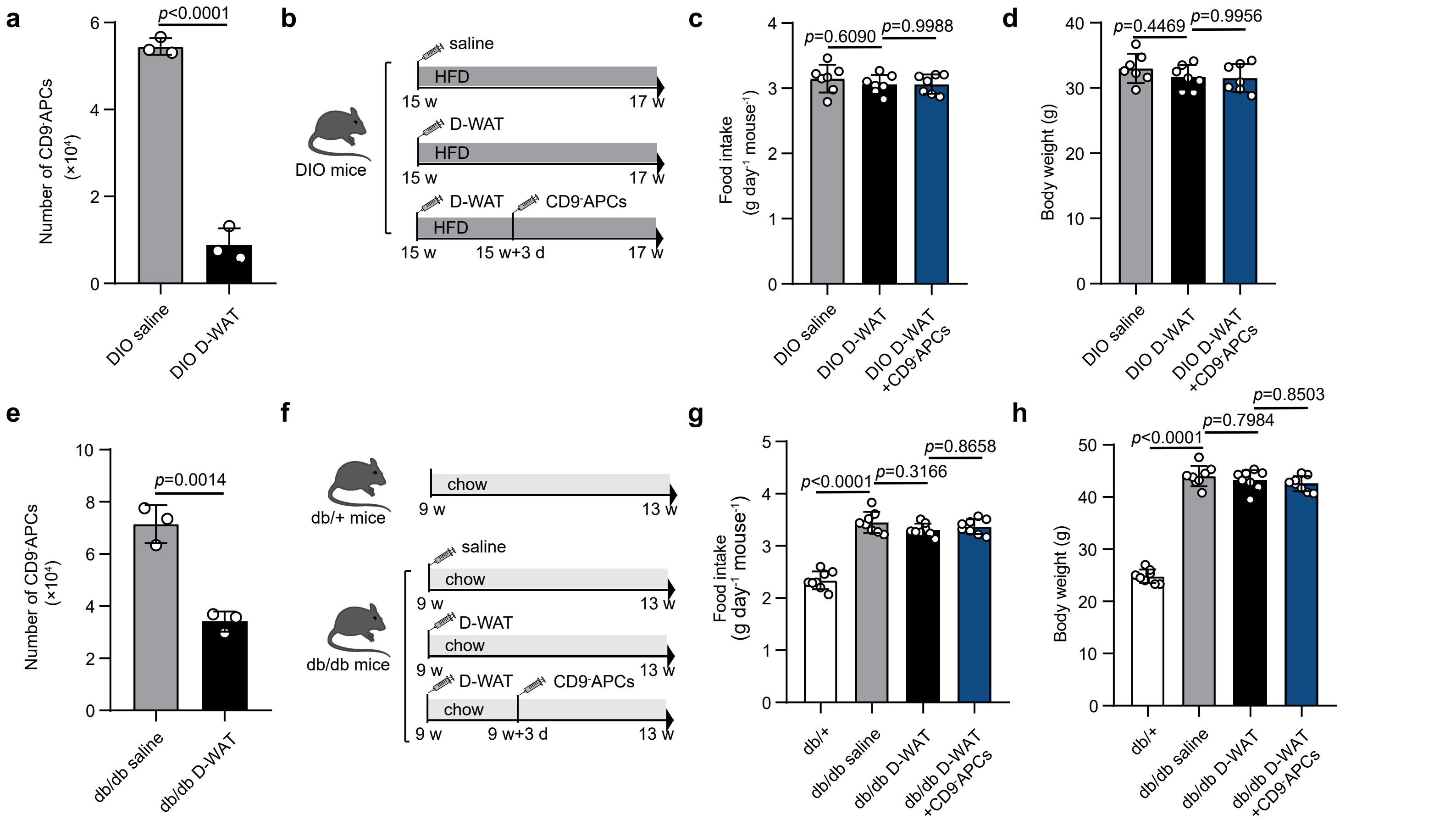
a
b
c
d
e
f
g
h
p<0.0001
Number of CD9- APCs (×10⁴)
DIO saline
DIO D-WAT
DIO mice
saline
D-WAT
CD9- APCs
HFD
15 w
17 w
15 w+3 d
p=0.6090
p=0.9988
Food intake (g day-1 mouse-1)
DIO D-WAT +CD9- APCs
p=0.4469
p=0.9956
Body weight (g)
p=0.0014
db/db saline
db/db D-WAT
db/+ mice
db/db mice
chow
9 w
13 w
9 w+3 d
p<0.0001
p=0.3166
p=0.8658
db/+
db/db D-WAT +CD9- APCs
p=0.7984
p=0.8503

Supplementary Fig. 7 This figure is related to Figure 7. **a** DIO mice were treated with D-WAT (8mg per mouse) or same dosage of saline. Three days later, the eWAT of each D-WAT or saline treated mouse was collected and absolute number of $CD9^{-}$APCs in the eWAT was determined by flow cytometry (n=3 per group). DIO, diet induced obesity. **b** Schematic representation of the intervention strategy. **c** Food intake (n=7 per group). **d** Body weight at the end of treatment (n=7 per group). **e** *Db/db* mice were treated with D-WAT (8mg per mouse) or same dosage of saline. Three days later, the eWAT of each D-WAT or saline treated mouse was collected and absolute number of $CD9^{-}$APCs in the eWAT was determined by flow cytometry (n=3 per group). **f** Schematic representation of the intervention strategy. **g** Food intake (n=8 per group). **h** Body weight at the end of treatment (n=8 per group). Data are means ± SD. For statistical analysis, **a,e,** Two-tailed unpaired Student's *t* test; for **c,d,j,h,** One-way ANOVA was used followed by Tukey's multiple comparison test. Source data are provided as a Source data file.

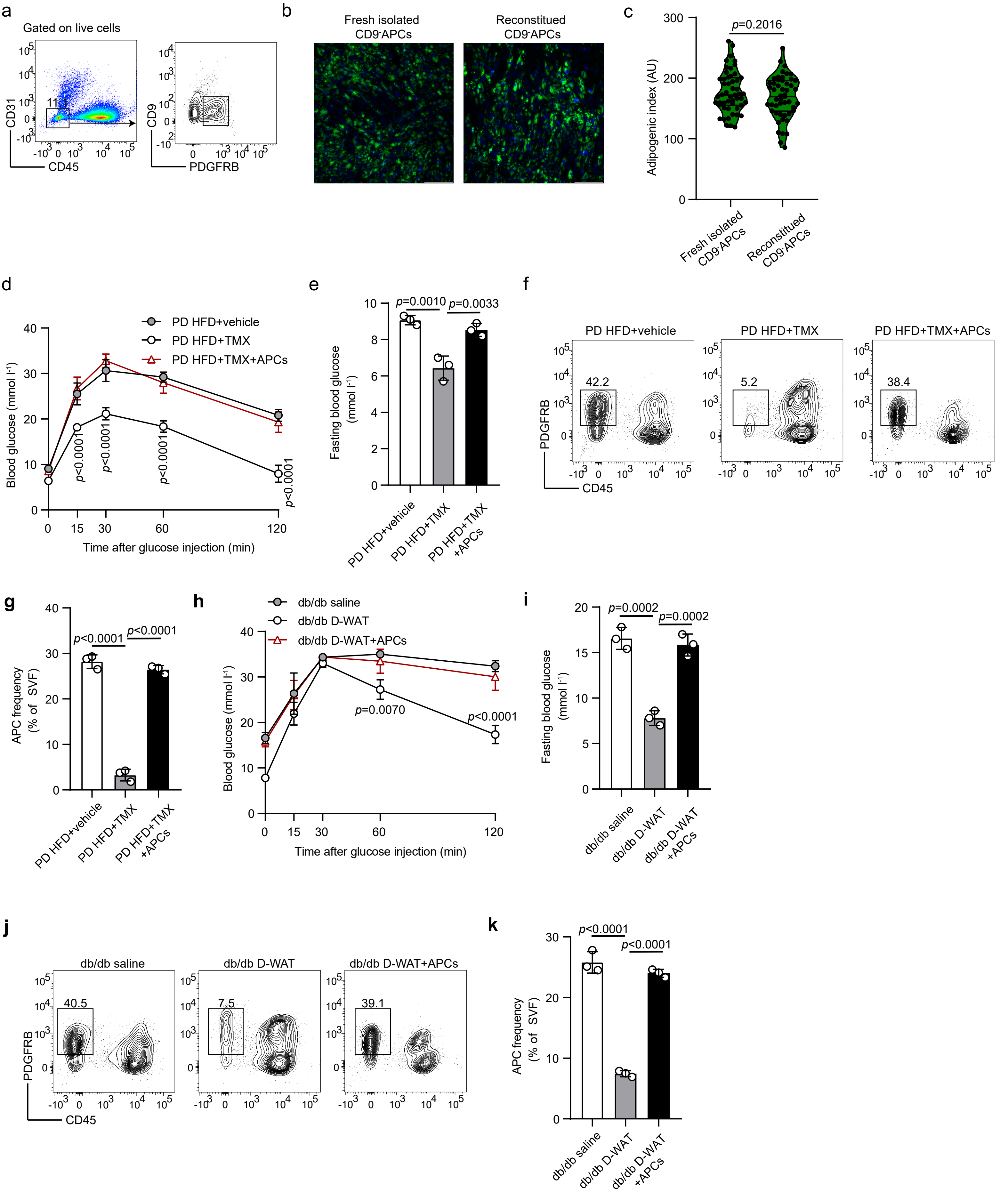

a
Gated on live cells
CD31
CD45
11.1
CD9
PDGFRB
b
Fresh isolated CD9⁻APCs
Reconstitued CD9⁻APCs
c
p=0.2016
Adipogenic index (AU)
Fresh isolated CD9⁻APCs
Reconstitued CD9⁻APCs
d
PD HFD+vehicle
PD HFD+TMX
PD HFD+TMX+APCs
Blood glucose (mmol l⁻¹)
Time after glucose injection (min)
p<0.0001
p<0.0001
p<0.0001
p<0.0001
e
p=0.0010
p=0.0033
Fasting blood glucose (mmol l⁻¹)
PD HFD+vehicle
PD HFD+TMX
PD HFD+TMX +APCs
f
PD HFD+vehicle
PD HFD+TMX
PD HFD+TMX+APCs
42.2
5.2
38.4
PDGFRB
CD45
g
p<0.0001
p<0.0001
APC frequency (% of SVF)
PD HFD+vehicle
PD HFD+TMX
PD HFD+TMX +APCs
h
db/db saline
db/db D-WAT
db/db D-WAT+APCs
Blood glucose (mmol l⁻¹)
p=0.0070
p<0.0001
Time after glucose injection (min)
i
p=0.0002
p=0.0002
Fasting blood glucose (mmol l⁻¹)
db/db saline
db/db D-WAT
db/db D-WAT +APCs
j
db/db saline
db/db D-WAT
db/db D-WAT+APCs
40.5
7.5
39.1
PDGFRB
CD45
k
p<0.0001
p<0.0001
APC frequency (% of SVF)
db/db saline
db/db D-WAT
db/db D-WAT +APCs

Supplementary Fig. 8 About 3.5×10^4 $CD9^-$ APCs isolated from untreated obese PD mice were transplanted into the eWAT of APC-depleted PD mice. One week after transplantation, reconstituted $CD9^-$ APCs were isolated by flow cytometry and cultured in 96 well plates. Meanwhile, freshly isolated $CD9^-$ APCs from vehicle treated obese PD mice were used as a control group. After exposure to standard differentiation factors *in vitro* for 8 days, the adipogenic capacity was analyzed (**a**-**c**). **a** Soring strategy of mouse $CD9^-$ APCs. **b** Staining of adipocytes (with Bodipy lipid stain). **c** Quantification of adipogenesis. For **d**-**g**, one week after tamoxifen injection in obese PD mice, total APCs from untreated obese PD mice were sorted and transplanted into APC depleted mice (n=3 per group). TMX, tamoxifen. Three weeks thereafter, glucose tolerance test was conducted (**d**) and fasting blood glucose levels were determined (**e**). After that, the eWAT of mice were collected to detect APC by flow cytometry (**f**,**g**). For **h**-**k**, three days after D-WAT treatment, total APCs from untreated *db/db* mice were sorted and transplanted into D-WAT treated *db/db* mice (n=3 per group). Three weeks later, glucose tolerance test was conducted (**h**) and fasting blood glucose levels were determined (**i**). Meanwhile, APCs in mouse eWAT were detected by flow cytometry (**j**) and quantified (**k**). Data are means ± SD. For statistical analysis, **c**, Two-tailed unpaired Student's *t* test; **d**,**e**,**g**-**i**,**k** One-way ANOVA was used followed by Tukey's multiple comparison test. Source data are provided as a Source data file.

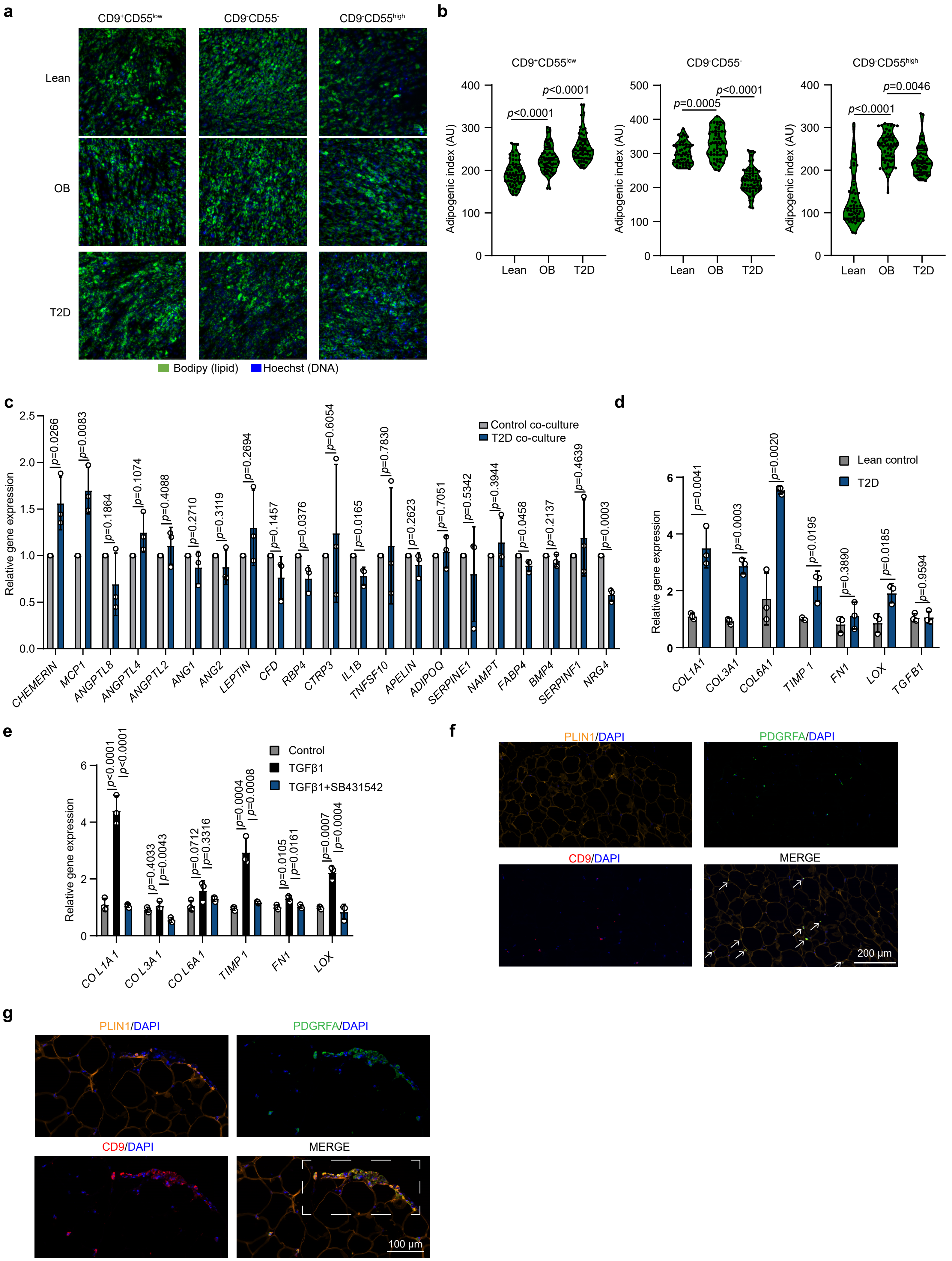

a
CD9+CD55low
CD9-CD55-
CD9-CD55high
Lean
OB
T2D
Bodipy (lipid)
Hoechst (DNA)
b
Adipogenic index (AU)
Lean
OB
T2D
p<0.0001
p=0.0005
p=0.0046
c
Relative gene expression
Control co-culture
T2D co-culture
CHEMERIN
MCP1
ANGPTL8
ANGPTL4
ANGPTL2
ANG1
ANG2
LEPTIN
CFD
RBP4
CTRP3
IL1B
TNFSF10
APELIN
ADIPOQ
SERPINE1
NAMPT
FABP4
BMP4
SERPINF1
NRG4
d
Lean control
T2D
COL1A1
COL3A1
COL6A1
TIMP 1
FN1
LOX
TGFB1
e
Control
TGFβ1
TGFβ1+SB431542
f
PLIN1/DAPI
PDGRFA/DAPI
CD9/DAPI
MERGE
200 μm
g
PLIN1/DAPI
PDGRFA/DAPI
CD9/DAPI
MERGE
100 μm

Supplementary Fig. 9 CD9^{+}CD55^{low} APCs from T2D patients retain adipogenic potential and exhibit a functional pro-fibrosis phenotype. **a**,**b** Distinct APC subpopulations were isolated from lean control subjects, participants with obesity, and T2D patients with obesity. Freshly isolated APC subpopulations were cultured with differentiation medium for 12 days to analyze adipogenic capacity. Lean, lean individuals; OB, participants with obesity; T2D, patients with type 2 diabetes and obesity. **a** Staining of adipocytes (with Bodipy lipid stain). **b** Quantification of adipogenesis. **c** Human primary adipocytes were co-cultured with CD9^{+}CD55^{low} APCs from lean control subjects or T2D patients with obesity for 3 days. Gene expression of adipokines were determined by qRT-PCR. Data are representative of three independent experiments. **d** mRNA levels of fibrosis related genes in CD9^{+}CD55^{low} APCs from lean control subjects (n=3) or T2D patients with obesity (n=3). **e** CD9^{+}CD55^{low} APCs from lean control subjects were treated with TGFβ1 and SB431542 for 3 days. The expression of fibrosis related genes was detected by qRT-PCR (n=3). **f**,**g** Omental adipose tissues from T2D patients with obesity were costained for PLIN1, PDGFRA, and CD9. Data are means ± SD. For statistical analysis, the following tests were used. **b**,**e** One-way ANOVA was used followed by Tukey's multiple comparison test. **c**,**d** Two-tailed unpaired Student's *t* test. Source data are provided as a Source data file.
