# Supplementary material for: Single-cell analysis reveals a subpopulation of adipose progenitor cells that impairs glucose homeostasis
Source: Nat Commun. 2024 Jun 6;15:4827. doi: 10.1038/s41467-024-48914-w (PMC11156882; doi:10.1038/s41467-024-48914-w)
Supplement: Supplementary file 3 — Reporting Summary [file 41467_2024_48914_MOESM3_ESM.pdf]

nature portfolio

Corresponding author(s): Yan Bi, Lei Shen

Last updated by author(s): May 7, 2024

Reporting Summary

Nature Portfolio wishes to improve the reproducibility of the work that we publish. This form provides structure for consistency and transparency in reporting. For further information on Nature Portfolio policies, see our Editorial Policies and the Editorial Policy Checklist.

Statistics

For all statistical analyses, confirm that the following items are present in the figure legend, table legend, main text, or Methods section.

n/a	Confirmed	
☐	☒	The exact sample size (*n*) for each experimental group/condition, given as a discrete number and unit of measurement
☐	☒	A statement on whether measurements were taken from distinct samples or whether the same sample was measured repeatedly
☐	☒	The statistical test(s) used AND whether they are one- or two-sided<br>*Only common tests should be described solely by name; describe more complex techniques in the Methods section.*
☒	☐	A description of all covariates tested
☐	☒	A description of any assumptions or corrections, such as tests of normality and adjustment for multiple comparisons
☐	☒	A full description of the statistical parameters including central tendency (e.g. means) or other basic estimates (e.g. regression coefficient) AND variation (e.g. standard deviation) or associated estimates of uncertainty (e.g. confidence intervals)
☐	☒	For null hypothesis testing, the test statistic (e.g. *F*, *t*, *r*) with confidence intervals, effect sizes, degrees of freedom and *P* value noted<br>*Give P values as exact values whenever suitable.*
☒	☐	For Bayesian analysis, information on the choice of priors and Markov chain Monte Carlo settings
☒	☐	For hierarchical and complex designs, identification of the appropriate level for tests and full reporting of outcomes
☐	☒	Estimates of effect sizes (e.g. Cohen's *d*, Pearson's *r*), indicating how they were calculated

Our web collection on statistics for biologists contains articles on many of the points above.

Software and code

Policy information about availability of computer code

Data collection	Single-cell suspensions were loaded onto a Chromium Single Cell Controller Instrument (10x Genomics) to generate single-cell gel beads in emulsions (GEMs). Illumina sequencer was used for RNA sequencing.
Data analysis	Softwares were used as follows:<br>Cell Ranger v3.0.2; HISAT2 v2.2.1; DEseq2 v1.26.0;<br>R packages:<br>Seurat v3.2.2; SeuratObject v4.0.0; harmony v1.0; Monocle2 v2.14.0; Monocle3 v0.2.2; Slingshot v1.4.0; org.Hs.eg.db v3.13.0; clusterProfiler v3.14.3; WGCNA v1.70-3; CellphoneDB v2.0.0; igraph v1.5.1; RColorBrewer v1.1-2; ComplexHeatmap v2.2.0; cowplot v1.1.1; dplyr v1.1.2; ggplot2 v3.4.2;<br>Python packages: Scrublet v0.2.3;<br>SPSS v22.0;<br>GraphPad Prism 9;<br>FlowJo v10.8.1;

For manuscripts utilizing custom algorithms or software that are central to the research but not yet described in published literature, software must be made available to editors and reviewers. We strongly encourage code deposition in a community repository (e.g. GitHub). See the Nature Portfolio guidelines for submitting code & software for further information.

Data

Policy information about availability of data

All manuscripts must include a data availability statement. This statement should provide the following information, where applicable:

- Accession codes, unique identifiers, or web links for publicly available datasets
- A description of any restrictions on data availability
- For clinical datasets or third party data, please ensure that the statement adheres to our policy

The reference files for human genome (GRCh38 version) and mouse genome (mm10 version) were built from https://www.10xgenomics.com/support/software/cell-ranger/latest/tutorials/cr-tutorial-mr. The processed public scRNA datasets were download from Gene Expression Omnibus: GEO, https://www.ncbi.nlm.nih.gov/geo/ including GSE128889 (Merrick et al. mouse SAT), GSE176067 (Emont et al. human WAT) and ArrayExpress database (www.ebi.ac.uk/arrayexpress) with the accession numbers E-MTAB-6677 (Schwalie et al. mouse SAT). Raw data of scRNA-seq in this study are uploaded in the Genome Sequence Archive for Human with accession number HRA002549 that are publicly accessible at https://ngdc.cncb.ac.cn/gsa-human/browse/HRA002549. Source data are provided as a Source Data file. All other information is available within the manuscript, supplementary information file, or upon request to the corresponding author. All original code has been deposited at GitHub and is available at https://github.com/dyanhua/APCs_scRNA-Seq. Any additional information required to reanalyze the data reported in this paper is available from the lead contact upon request.

Research involving human participants, their data, or biological material

Policy information about studies with human participants or human data. See also policy information about sex, gender (identity/presentation), and sexual orientation and race, ethnicity and racism.

Reporting on sex and gender	The gender of participants enrolled in this study has been disclosed in Supplementary Table 1.
Reporting on race, ethnicity, or other socially relevant groupings	All subjects enrolled in this study are Han Chinese.
Population characteristics	Demographics of the enrolled subjects are summarized in Supplementary table1.
Recruitment	A total of 48 subjects, including 37 participants with obesity underwent laparoscopic Roux-en-Y gastric bypass (RYGB) surgery, and 11 lean control subjects received elective abdominal surgery (e.g. hernia or hemangioma resection) were enrolled at Drum Tower Hospital Affiliated to Nanjing University Medical School. All subjects were enrolled following the inclusion and exclusion criteria, as described in the main text.
Ethics oversight	This study was approved by the Ethics Review Committee of Nanjing Drum Tower Hospital Affiliated to Nanjing University Medical School (Approval number: 2017-030-02).

Note that full information on the approval of the study protocol must also be provided in the manuscript.

Field-specific reporting

Please select the one below that is the best fit for your research. If you are not sure, read the appropriate sections before making your selection.

☒ Life sciences ☐ Behavioural & social sciences ☐ Ecological, evolutionary & environmental sciences

For a reference copy of the document with all sections, see nature.com/documents/nr-reporting-summary-flat.pdf

Life sciences study design

All studies must disclose on these points even when the disclosure is negative.

Sample size	Sample size was chosen based on knowledge from previous studies in the field. Key experiments were repeated by 2 independent researchers. For animal experiments, we tried our best to ensure that the experimental mice were of the same age. According to the number of newborn mice, the number of each experimental group was about 5-8. Variability in the qPCR analysis or other in vitro assays used in this study tends to be low, so n>=3 is the accepted norm in this field.
Data exclusions	No data were excluded in this study.
Replication	All experiments were replicated at least twice as mentioned in the manuscript. In vitro experiments were performed with at least 3 replicates. All replication attempts indicated similar results.
Randomization	Animals were randomly grouped into control and treated groups for all the experiments in this study. Samples were allocated to groups based on disease status.
Blinding	The quantitative histological analysis was performed by two investigators who had no knowledge of the origin of the slides. For quantifications that were done with flow cytometry, blinding was performed by labeling the test tubes numerically without prior knowledge of the treatment of the sample.

Reporting for specific materials, systems and methods

We require information from authors about some types of materials, experimental systems and methods used in many studies. Here, indicate whether each material, system or method listed is relevant to your study. If you are not sure if a list item applies to your research, read the appropriate section before selecting a response.

Materials & experimental systems

n/a	Involved in the study
☐	☒ Antibodies
☒	☐ Eukaryotic cell lines
☒	☐ Palaeontology and archaeology
☐	☒ Animals and other organisms
☒	☐ Clinical data
☒	☐ Dual use research of concern
☒	☐ Plants

Methods

n/a	Involved in the study
☒	☐ ChIP-seq
☐	☒ Flow cytometry
☒	☐ MRI-based neuroimaging

Antibodies

Antibodies used

APC anti-human CD45 (clone HI30), Biolegend, cat#304012, dilution 1:100;
FITC anti-human CD31 (clone WM59), BD Biosciences, cat#557508, dilution 1:100;
PE anti-human CD140a (clone αR1), BD Biosciences, cat#556002, dilution 1:100;
BUV395 anti-human CD9 (clone M-L13), BD Biosciences, cat#743052, dilution 1:100;
BV510 anti-human CD55 (clone IA10), BD Biosciences, cat#742678, dilution 1:100;
AF700 anti-human ICAM1 (clone HA58), eBioscience, cat#56-0549-42, dilution 1:100;
BV421 anti-human CD142 (clone HTF-1), BD Biosciences, cat#744003, dilution 1:100;
APC anti-mouse CD45 (clone 30-F11), BD Biosciences, cat#559864, dilution 1:100;
BV786 anti-mouse CD31 (clone MEC13.3), BD Biosciences, cat#740870, dilution 1:100;
PE anti-mouse CD140b (clone APB5), Biolegend, cat#136005, dilution 1:100;
BB700 anti-mouse CD9 (clone KMC8), BD Biosciences, cat#742131, dilution 1:100;
BV421 anti-mouse CD26 (clone H194-112), BD Biosciences, cat#740021, 1:100.
Dead cells were stained with fixable viability stain 780,BD Biosciences, cat#565388, dilution 1:1000.
Immunofluorescence antibodies:
mouse monoclonal anti-PDGFRb (clone 42G12), Abcam, cat#ab69506, dilution 1:50;
rabbit monoclonal anti-PDGFRa (clone EPR22059-270), Abcam, cat#ab203491, dilution 1:50;
rabbit monoclonal anti-PLIN1 (clone EPR3753-2), Abcam, cat#ab172907,dilution 1:50;
rabbit monoclonal anti-CD9 (clone EPR23105-121), Abcam, cat#ab236630, dilution 1:50.

Validation

The validation of all of the antibodies depends on product datasheet and published literature.

Animals and other research organisms

Policy information about studies involving animals; ARRIVE guidelines recommended for reporting animal research, and Sex and Gender in Research

Laboratory animals

The mice used in this study were included in method and figure legend section of the manuscript. C57BL/6, db/db,Pdgfra-CreERT2 mice and Rosa26-LSL-DTA mice were used. All animals were purchased from GemPharmatech (Nanjing, China). All mice were kept in a SPF level facility and provided with adequate food and water, as well as normal light, temperature and humidity (12-h light/dark cycle, 60-70% humidity). Mice used in this study were 6-8 weeks old unless otherwise indicated in the text.

Wild animals

The study did not involve wild mice.

Reporting on sex

All mice used in this study were males.

Field-collected samples

The study did not involve samples collected from the field.

Ethics oversight

All animal studies were approved by the Research Animal Care Committee of Drum Tower Hospital Affiliated to Nanjing University Medical School, Nanjing, China.

Note that full information on the approval of the study protocol must also be provided in the manuscript.

Plants

Seed stocks	N/A
Novel plant genotypes	N/A
Authentication	N/A

Flow Cytometry

Plots

Confirm that:

- [x] The axis labels state the marker and fluorochrome used (e.g. CD4-FITC).
- [x] The axis scales are clearly visible. Include numbers along axes only for bottom left plot of group (a 'group' is an analysis of identical markers).
- [x] All plots are contour plots with outliers or pseudocolor plots.
- [x] A numerical value for number of cells or percentage (with statistics) is provided.

Methodology

Sample preparation	Periumbilical adipose tissue samples at the omental region were obtained from participants with obesity, T2D patients with obesity, and control subjects perioperatively. Adipose tissue samples (approximately 10 g) were collected and transported to laboratory immediately. For human SVFs preparation, fresh adipose samples were cut into small pieces and digested with 0.1% type II collagenase. Mouse adipose SVFs were prepared using 0.1% type I collagenase . Briefly, eWAT was physically dissociated using scissors and incubated for 45 minutes in digest solution (1mg ml-1 type I collagenase in RPMI supplemented with 5% fetal calf serum, 1% L-glutamine, 1% penicillin-streptomycin, and 10 mM HEPES). Resulting dissociated tissue was passed through 100µm nylon mesh, centrifuged, and adipocytes were removed from the supernatant. Red blood cells were lysed using RBC lysis buffer.
Instrument	Samples were analyzed on an LSR Fortessa II flow cytometer (BD Biosciences) or sorted on a FACSAria II flow cytometer (BD Biosciences)
Software	All flow data were acquired by BD FACSDiva software and analyzed by FlowJo software version 10.8.1 (Tree Star, Inc)
Cell population abundance	We provided FACS-gating strategies for all flow cytometry analysis and FACS cell sorting that confirm the abundance of the analyzed or sorted relevant cell populations.
Gating strategy	We provided FACS-gating strategies for all flow cytometry analysis and FACS cell sorting, specifying the preliminary FSC/SSC gates and how positive and negative staining cell populations are defined. FMO was used as a control.

- [x] Tick this box to confirm that a figure exemplifying the gating strategy is provided in the Supplementary Information.
