# Supplementary material for: Single-cell analysis reveals a subpopulation of adipose progenitor cells that impairs glucose homeostasis
Source: Nat Commun. 2024 Jun 6;15:4827. doi: 10.1038/s41467-024-48914-w (PMC11156882; doi:10.1038/s41467-024-48914-w)
Supplement: Supplementary file 2 — Peer Review File [file 41467_2024_48914_MOESM2_ESM.pdf]

nature portfolio

Peer Review File

Single-cell analysis reveals a subpopulation of adipose progenitor cells that impairs glucose homeostasis

Open Access This file is licensed under a Creative Commons Attribution 4.0 International License, which permits use, sharing, adaptation, distribution and reproduction in any medium or format, as long as you give appropriate credit to the original author(s) and the source, provide a link to the Creative Commons license, and indicate if changes were made. In the cases where the authors are anonymous, such as is the case for the reports of anonymous peer reviewers, author attribution should be to 'Anonymous Referee' followed by a clear attribution to the source work. The images or other third party material in this file are included in the article's Creative Commons license, unless indicated otherwise in a credit line to the material. If material is not included in the article's Creative Commons license and your intended use is not permitted by statutory regulation or exceeds the permitted use, you will need to obtain permission directly from the copyright holder. To view a copy of this license, visit http://creativecommons.org/licenses/by/4.0/.

REVIEWER COMMENTS

Reviewer #1 (Remarks to the Author):

The authors have identified an adipose progenitor cell (APC) sub population in human visceral adipose tissue that participates in Type 2 Diabetes (T2D) development. By comparing the abundance of APC subpopulations in lean, obese and obese T2D, it appeared that the CD9 APC subset was higher in obese and T2D patients compared to lean donors. Then, different approaches strongly suggest that the CD9+CD55low subset is associated with the development of T2D, and an elegant series of experiments functionally demonstrated that this APC subpopulation plays a crucial role in driving T2D progression in diet-induced obese mice. Finally, a detailed analysis of secretomes revealed that this pathological APC subpopulation displays a chemotactic capacity and regulates adipose lipolysis, both capacities tested in vitro and in vivo.
The manuscript is clearly written and reports a very significant work in the field. Data support the conclusions. However, some claims should be modulated (see below). The methodology is sound.

Major concerns
1) Fig.1h: CD55+ APCs are reported as multipotent progenitors. Is this population similar to the CD26+ APC subpopulation previously reported (Merrick et al. 2019)?

2) A high abundance of PDGFRα+CD9high APCs in adipose tissues of obese patients has been previously reported. This subpopulation correlates with fibrosis level of the tissue and with T2D (Marcellin et al. 2017; Ref 14 in the submitted manuscript). Therefore, the sentence “APC CD9 cells appear to be a novel subset of APC in human visceral adipose tissue” (p5, line 20) should be modulated.

3) According to the FACS-based selection of APCs illustrated in Fig.2b, the sub population having a potential fibrotic behaviour could be CD9-CD55- giving rise to ICAM1+ cells. How the PDGFRα+CD9high could be mapped in the CD9+CD55high sub populations should be more discussed.

4) Images presented in Fig.3a and g are of a weak quality and should be improved to be more convincing.

5) FACS analyses reports the maintenance of the human APCs several weeks after transplantation in immunocompetent mice, likely thanks to the adoptive cell transfer approach used by the authors. The approach used has to be described in details.

6) Authors performed experiments in obese mice to deplete all the APC subpopulations and to investigate their role in obesity-related metabolic disorders. They conclude that APCs depletion is sufficient to achieve metabolic benefits. This conclusion should be more discussed and the authors should propose how to integrate in this model data showing that APCs are critical to generate new adipocytes in response to pathological conditions (Virtue, S.; Vidal-Puig, A. Adipose Tissue Expandability, Lipotoxicity and the Metabolic Syndrome—An Allostatic Perspective.Biochim. Biophys.

Acta 2010, 1801, 338–349.s). Previously, Dacquinag et al. (Cell Death and Differentiation, 2015) performed a similar experiment using a peptide D-WAT, similar than this used in the submitted manuscript, and showed that APC depletion prevents dyslipidemia. The authors proposed that the metabolic benefit of the APC depletion was due to beiging of white adipose tissue. These data should also be incorporated in the discussion.

Reviewer #2 (Remarks to the Author):

The article ” scRNA-seq reveals a subpopulation of adipose progenitor cell that impairs glucose homeostasis” by H.Wang et al. identifies CD9+CD55low adipocyte progenitor cells (APC) as a cell subpopulation increased in human obesity-related T2D and suggest that these cells have a direct detrimental impact on adipose tissue and systemic metabolism. The regulatory APC populations have been proposed by several previous studies and this one is an additional report that such cell populations might exist. Both human APCs and multiple murine models are employed and the study presents interesting findings with potential therapeutic impact. However, there are few pitfalls that need to be addressed before strong conclusions regarding the role of CD9+CD55low cell population can be drawn. The major problem are the lack of correct controls in the cell transplantation experiments and the lack of specificity towards a particular APC population of cells (in this case CD9+CD55low) in the APC depletion experiments.

These are specific comments:
Transplantation experiments.
- Data shown in figure 3 are obtained only from 3-5 animals and unfortunately doesn’t have a suitable control. The injection of human cells is a large stress factor and could have caused inflammation and immune response that subsequently could have affected WAT and systemic insulin sensitivity. Cells that are not expected to contribute to hyperglycemia should have been used as a control (for example CD55high) and effects caused by CD9+CD55low transfer should have been compared to the effects obtained with the injection of other APC population. PBS is not a suitable control and there is a large possibility that the results are misleading.
o For the experiments shown in 3a, were the percentages of remaining APCs quantified?
- Transplantation data presented in figure 5 also lack reliable control. The control should have been CD9+CD55low cell population from lean individuals. Mice that didn’t receive any transplantation cannot serve a control for transplanted ones due to multiple side effects of such intervention. If the authors state, that CD9+CD55low population is changing phenotype in obesity/T2D, the direct comparison of this cell population from lean and obese/T2D patients would be needed.

Flow cytometry characterization of APCs
- Please add CD34 expression in your progenitor violin plots 1d as a general marker for human adipocyte progenitors.
- This reviewer would like to see FMO controls for CD31, PDGFRA, CD55, CD9, ICAM and CD142 in the same picture as 2a. The definition of positive cells is not quite obvious and flow cytometry is not well-described in the method section.

Depletion/treatment experiments. Major problem with the depletion experiments using D-WAT and tamoxifen treatments are non-specific depletion of all APC populations therefore although these data proves the importance of APCs in general they don’t address a specific role of CD9+CD55low

cell populations
- Figure 6. Do the mice gain weight on HFD between moths 3 and 4 after they are treated with tamoxifen or vehicle (fig 6)? The result that depletion of APCs improves metabolic phenotype goes against multiple previous data where studies suggested that differentiation of new adipocytes is metabolically beneficial compared to the increased size of mature adipocytes. Could it be that tamoxifen injection and APC depletion has non-specific effects that are associated with metabolic phenotype.
Could the authors show the data of weight, food intake and fat cell size in the PD mice injected with tamoxifen on HFD. In addition, how can the authors exclude that tamoxifen alone has a metabolic effect when mice are on HFD. There was no effect on chow, but metabolic conditions are changing substantially in obesity.
- Figure 7. In the treatments of DIO and db/db mice with D-WAT, which APC population was depleted? Please present the data of depletion for different APC populations? What was food intake rate, what was the weight of these mice after the treatment.
Bioinformatic analysis
- Extended figure 2. Could you please include references for clusters shown in ext fig. 2c,d. The references are missing in both the text and the figure legend therefore it's difficult to understand that the authors mean… Do the authors map their clusters to cells obtained from visceral or subcutaneous clusters.
- CD9 cells do not correspond to any of previously identified clusters. Could the authors comment on this fact? What is the difference from the multiple other studies and the ones shown in extended figure 2? What about purity of the sample?
- CD9-/CD55-/ICAM1- population was decreased in obese and obese T2D patients. What are these cells? Do they express CD34? PDRFRA? Can they differentiate or if this decrease only relative as CD9+ cell population is increased?

Other:
- Could you please change colors in figure 1c to visualize contribution of each patient to the clusters,
- In general, figure legends needs to be expanded to . In many cases, it is not clear what is shown, and n numbers are lacking. Please add n to each panel where relevant.
- The lightness of the microscopy pictures should be increased. It is impossible to see the staining (neither on the screen, nor in the printed version).
- The method section does not provide all the necessary details to reproduce the data shown.

Reviewer #3 (Remarks to the Author):

Wang, et al. utilize single-cell RNA sequencing (scRNA-seq) to study adipose tissue composition of type 2 diabetes (T2D) patients. The key findings are:
1. T2D patients have altered adipose progenitor cell (APC) composition.
2. The CD9+ APCs population is a distinct APC subpopulation and contributes to T2D development.
3. Depletion of CD9+ APCs in mice improves obesity-related glycemic disturbances.

The role of adipose progenitors in the development of T2D is poorly understood. Wang, et al. seek to address this critical question through the application of single-cell RNA-sequencing technology and in vivo APC transfer assays. The study demonstrates significant dedication to the recruitment, processing, and analysis of clinically relevant donor samples. As presented, however, the findings

presented in this manuscript should be better supported with a more comprehensive interpretation of existing single-cell data, a critical review of underlying assumptions, and improvement in the experimental design.

Major concerns:

• In Fig. 1, the authors characterize the stromal vascular fractions derived from human visceral adipose tissues. It is important to recognize that the captured transcriptomic profiles represent a snapshot of the dynamic tissue. Throughout the manuscript, APCs_CD9 is depicted as a stable subpopulation with a distinct role. However, the slingshot trajectory analysis (Fig. 1g) positions the APCs_CD9 cluster in a transition state, situated between the APCs_CD55 progenitors and the further developed APCs_ICAM1 (committed preadipocytes) and APCs_CD142 (adipogenesis-regulatory cells). The APCs_CD9 cells have intermediate levels of marker gene expressions (Fig. 1d) and enrichment (Fig. 1h, Extended data fig. 2c,d) in between those of early progenitors and developed cells. Altogether, the profile of APCs_CD9 cells resembles a dynamic intermediate developmental cell state and not a static pathogenic cell population.

• In the subsequent figures, the authors designed experiments with the assumption that CD9+CD55low is a distinct cell type. In Fig. 4 and Fig. 5, the authors focus solely on comparing CD9+CD55low cells derived from lean and obese T2D donors. It is important to consider the possibility that the distinctions observed between lean and obese donors may not be exclusive to CD9+ cells but could potentially extend to all APCs.

• An appropriate control group is missing in the APCs transfer experiments presented in Fig. 3, Fig. 5g-n, and Extended data Fig. 5f,g. The use of PBS as a control does not appropriately control for the impact of allogenic cell injection. Does the injection of CD9-CD55high cells also induce lipolysis?

• The authors claim that the majority of APCs in the adipose tissue of obese mice are CD9+ cells, and consequently, the metabolic effects induced by the ablation of all APCs presented in Fig. 6 and Fig. 7 are attributed to the removal of CD9+ APCs. The assumption is based on the flow cytometry profile derived from a diet-induced obesity mouse model, which suggests that CD9+ APCs make up ~75% of the total APCs in the obese mouse model. However, this attribution is valid only if the ablation of CD9- APCs has no discernable physiological impact.

• The authors have integrated the scRNA-seq results from this study with previously published APCs datasets (page 5, line 17-18, Fig. 1h, Extended data fig. 2c,d). However, the authors did not indicate which reference studies were used.

• On page 6, in line 9, the authors have misinterpreted the PCA plot. They assert that CD9+CD55low APCs exhibit the most distinct separation from the rest of the dataset. However, it is important to note that the first principal component, responsible for 40% of the dataset's variance, distinguishes the CD55+ population from the other populations. The second principal component, which accounts for 26% of the variance, is the one that distinguishes the CD9+ population. Therefore, it is the CD55+ population that exhibits the most obvious separation from the others.

• In Fig. 4c, the authors have elected to only highlight proteins enriched in the control/non-T2D group. However, given that the primary focus is on examining the secretome of APCs derived from T2D patients, it would be more informative to highlight specific proteins enriched in the T2D group

to be highlighted. Additionally, the protein names are presented as two clusters, raising the question of whether the heatmap is aligned correctly with the protein annotations.

• Different marker genes for the APCs_CD9 cells are employed in different. Genes analyzed by qPCR, as depicted in Extended Data Fig. 3b, show limited overlap with the APCs_CD9 marker genes presented in Extended Data Fig. 2a, or the CD9+CD55low APCs marker genes displayed in Fig. 2d.

Minor concerns:

• The gene SFRP4 (secreted frizzled related protein 4) is incorrectly spelled as SFBP4 on page 4, line 17.

• The authors should offer a rationale for selecting the APCs_CD55 cluster as the root state of the Slingshot pseudo-time analysis.

• On page 9, line 19, it should read “little” instead of “litter.”

• The authors should clarify in the figure legends the meaning of the blue data points in the volcano plot presented in Fig. 5a.

<u>Reviewer #1:</u>

Major concerns:

1. Fig.1h: CD55+ APCs are reported as multipotent progenitors. Is this population similar to the CD26+ APC subpopulation previously reported (Merrick et al. 2019)?

<u>Response:</u>

We thank the reviewer for raising this point.

To determine whether CD55$^+$APC is related to CD26$^+$APC, the signature genes of CD55$^+$APC were compared to that of three APC subsets defined by Merrick *et al.* As shown in Fig. 1g, the signature genes of APCs_CD55 subpopulation were enriched in CD26$^+$ (encoded by *DPP4*) APC subpopulation, which was previously identified as multipotent progenitor cell by Merrick *et al*. We have added the relevant reference in the revised manuscript (Page 6, line 11).

Moreover, we compared the expression of signature genes of CD26$^+$APC subpopulation reported by Merrick *et al.* to the four APC subsets discovered in our study. As shown in reply Figure 1, the highest expression of signature genes of CD26$^+$APC was detected in our APCs_CD55 subpopulation, but not in other three subpopulations.

These data support that APCs_CD55 subpopulation strongly resembled previously identified CD26$^+$APC subpopulation.

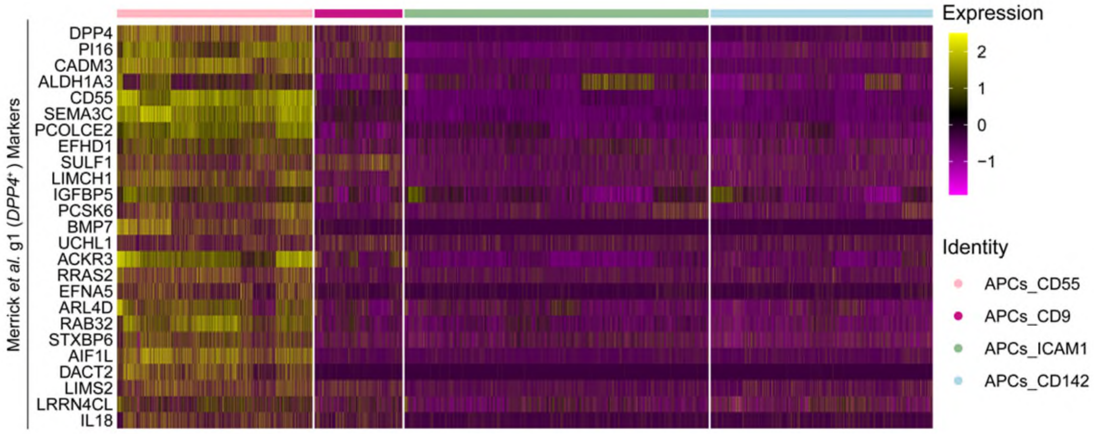

Reply Figure 1: Comparison of the data reported by Merrick *et al.* with our dataset. A heatmap of the signature genes of the *DPP4*$^+$APC expressed in the four APC subsets defined in our study.

2. A high abundance of PDGFRα+CD9high APCs in adipose tissues of obese patients has been previously reported. This subpopulation correlates with fibrosis level of the tissue and with T2D (Marcellin et al. 2017; Ref 14 in the submitted manuscript). Therefore, the sentence "APC CD9 cells appear to be a novel subset of APC in human visceral adipose tissue" (p5, line 20) should be modulated.

Response:

In accord with the reviewer's suggestion, we removed this sentence in the revised manuscript.

3. According to the FACS-based selection of APCs illustrated in Fig.2b, the sub population having a potential fibrotic behavior could be CD9-CD55- giving rise to ICAM1+ cells. How the PDGFRα+CD9high could be mapped in the CD9+CD55high sub populations should be more discussed.

Response:

We appreciate your comment.

As illustrated in Supplementary Fig. 2b, genes related to fibrosis were not enriched in APCs_ ICAM1. GO analysis further indicated that genes associated with the extracellular structure organization pathway were not enriched in the APCs_ ICAM1 (Supplementary Fig. 2c).

PDGFRα^{+}CD9^{high} APCs reported by Marcellin *et al.* exhibited two dominant features: high expression of CD9 and pro-fibrosis capacity. In our study, as depicted in Supplementary Fig. 8d, expression of genes related to fibrosis was highly upregulated in CD9^{+}CD55^{low} APCs from T2D patients compared to that from lean subjects. Thus, CD9^{+}CD55^{low} APCs identified in our study corresponds to PDGFRα^{+}CD9^{high} APCs reported by Marcellin *et al.*

We have included this description in the *Discussion* section (Page 23, lines 7-9).

4. Images presented in Fig.3a and g are of a weak quality and should be improved to be more convincing.

Response:

Thank you very much.

We have replaced the micrographs with better quality of the figures (Fig. 3a, Fig. 3g,

Fig. 6k, Fig. 7h, and Fig. 7s) in the revised manuscript.

5. FACS analyses reports the maintenance of the human APCs several weeks after transplantation in immunocompetent mice, likely thanks to the adoptive cell transfer approach used by the authors. The approach used has to be described in details.

Response:

In response to this suggestion, we have added the details of adoptive cell transfer to the *Method* section (Page 25, lines 9-21; Page 33, lines 17-21; Page 34, lines 1-9).

6. Authors performed experiments in obese mice to deplete all the APC subpopulations and to investigate their role in obesity-related metabolic disorders. They conclude that APCs depletion is sufficient to achieve metabolic benefits. This conclusion should be more discussed and the authors should propose how to integrate in this model data showing that APCs are critical to generate new adipocytes in response to pathological conditions (Virtue, S.; Vidal-Puig, A. Adipose Tissue Expandability, Lipotoxicity and the Metabolic Syndrome—An Allostatic Perspective.Biochim. Biophys. Acta 2010, 1801, 338–349.s). Previously, Dacquinag et al. (Cell Death and Differentiation, 2015) performed a similar experiment using a peptide D-WAT, similar than this used in the submitted manuscript, and showed that APC depletion prevents dyslipidemia. The authors proposed that the metabolic benefit of the APC depletion was due to beiging of white adipose tissue. These data should also be incorporated in the discussion.

Response:

We thank the reviewer for raising these valuable comments. Although we could not rule out the possibility that APC depletion may interfere with adipogenesis, the influence may be minor. The reasons are: (1) Previous studies using different lineage tracing mouse models investigated the timing of APC activation and adipogenesis in response to HFD, showing that new adipocytes form in eWAT between five and eight weeks of HFD feeding[1,2]. In this study, mice were fed a HFD for more than eight weeks before APC were depleted. So, by the time of APC depletion, adipogenesis may not be affected. (2) Another study has reported that $CD9^+$ APCs are the predominant subset in

the eWAT of obese mice and can hardly differentiate into mature adipocyte[3]. Consistent with previous studies, depleted APCs in this study were mainly $CD9^+$ APCs.

Nevertheless, whether APC depletion impedes adipocyte generation is an important issue to investigate. Accordingly, we have added the following text in the *Discussion* section (Page 20, lines 3-5):

“In addition, it is worth noting that APCs are critical to generate new adipocytes during the progression of obesity, whether APC depletion impedes adipocyte generation needs further investigation.”

References:

1. Wang QA, Tao C, Gupta RK, Scherer PE. Tracking adipogenesis during white adipose tissue development, expansion and regeneration. Nature medicine 19, 1338-1344 (2013).
2. Jeffery, E., Church, C.D., Holtrup, B., Colman, L. & Rodeheffer, M.S. Rapid depot-specific activation of adipocyte precursor cells at the onset of obesity. Nature cell biology 17, 376-385 (2015).
3. Marcelin, G., et al. A PDGFRalpha-Mediated Switch toward CD9(high) Adipocyte Progenitors Controls Obesity-Induced Adipose Tissue Fibrosis. Cell metabolism 25, 673-685 (2017).

We appreciate the second issue raised by the reviewer.

Study from Dacquinag *et al.* indicates that APC depletion could induce beige adipocyte differentiation. The experimental strategy performed in their study was different from that of ours. They depleted mouse APCs through *s.c.* injection of D-WAT peptide before HFD feeding was given, whereas APCs were depleted through *in situ* injection in eWAT of obese mice in our study. Since functionality of APC changes during obesity progression, depleting APCs at different stage of obesity may cause different consequences.

As suggested, we have added the following sentences in the *Discussion* section (Page 20, lines 5-8):

“Moreover, another study reported that APC depletion before the development of obesity could induce beige adipocyte differentiation, indicating that depleting APCs at different stage of obesity may cause diverse consequences.”

Reviewer #2:

Transplantation experiments.

1. Data shown in figure 3 are obtained only from 3-5 animals and unfortunately doesn't have a suitable control. The injection of human cells is a large stress factor and could have caused inflammation and immune response that subsequently could have affected WAT and systemic insulin sensitivity. Cells that are not expected to contribute to hyperglycemia should have been used as a control (for example CD55high) and effects caused by CD9+CD55low transfer should have been compared to the effects obtained with the injection of other APC population. PBS is not a suitable control and there is a large possibility that the results are misleading.

Response:

Thank you for your valuable suggestion. Your constructive suggestion was very helpful for us to improve the quality of this work.

In accord with the reviewer's suggestion, we included $CD9^-$ APCs from T2D patients as a control group. The results showed that mice received $CD9^+CD55^{low}$ APCs transplantation had increased fasting glucose and impaired glucose tolerance compared to that received $CD9^-$ APCs transplantation (Fig. 3d-f, m-o).

These findings support that $CD9^+CD55^{low}$ APCs, not $CD9^-$ APCs, promote glucose intolerance. We have updated the *Results* in the revised manuscript (Page 9, lines 13-22; Page 10, lines 12-22).

2. For the experiments shown in 3a, were the percentages of remaining APCs quantified?

Response:

Thank you for raising this question.

The frequencies of remaining APC subpopulations were detected by flow cytometry (Supplementary Fig. 4a-c). After D-WAT treatment, frequencies of both $CD9^+$ APCs and $CD9^-$ APCs in the eWAT of C57BL/6 mice were decreased. These data have been included in the revised manuscript (Page 9, lines 11-13).

3. Transplantation data presented in figure 5 also lack reliable control. The control should have been CD9+CD55low cell population from lean individuals. Mice that didn't receive any transplantation cannot serve a control for transplanted ones due to multiple side effects of such intervention. If the authors state, that CD9+CD55low population is changing phenotype in obesity/T2D, the direct comparison of this cell population from lean and obese/T2D patients would be needed.

Response:

Thanks for your constructive comments.

We used $CD9^{+}CD55^{low}$ APCs from lean control subjects as the control. Compared to the mice transplanted with $CD9^{+}CD55^{low}$ APCs from lean control subjects, mice transplanted with $CD9^{+}CD55^{low}$ APCs from obese T2D patients showed higher levels of serum FFA and glycerol (Fig. 5k,l). Accordingly, we have revised the text in the *Result* section (Page 13, line 20-22; Page 14, lines 1-6).

Flow cytometry characterization of APCs

4. Please add CD34 expression in your progenitor violin plots 1d as a general marker for human adipocyte progenitors.

Response:

Thanks for your advice. Expression of CD34 has been included in Fig. 1d in the revised manuscript.

5. This reviewer would like to see FMO controls for CD31, PDGFRA, CD55, CD9, ICAM and CD142 in the same picture as 2a. The definition of positive cells is not quite obvious and flow cytometry is not well-described in the method section.

Response:

Thanks for your positive comments.

All FMO controls have been added in the revised manuscript (Supplementary Fig. 3b). Meanwhile, more details about flow cytometry have been added in the *Method* section (Page 31, lines 15-22; Page 32, lines 11-17).

6. Depletion/treatment experiments. Major problem with the depletion experiments using D-WAT and tamoxifen treatments are non-specific depletion of all APC populations therefore although these data proves the importance of APCs in general they don't address a specific role of CD9+CD55low cell populations.

Response:

We appreciate your valuable comments to improve our experiments. Currently, it is technically hard to deplete one specific APC subpopulation. Therefore, we reconstituted $CD9^-$ APCs in APC-depleted mice to exclude the influence of $CD9^-$APC depletion on glycemic metabolism:

1. In obese PD mice, approximately 3.5×10^4 $CD9^-$ APCs were depleted after tamoxifen treatment. So, as shown in Fig. 6a, $CD9^-$ APCs from obese PD mice were transplanted into APC-depleted mice. In agreement with our findings in Fig. 3 that $CD9^-$ APCs did not impair glycemic homeostasis, APC depletion significantly improved glycemic disturbance in obese PD mice, whereas $CD9^-$APC recovery did not alter these metabolic benefits.

2. In DIO and *db/db* mice, D-WAT treatment depleted approximately 4×10^4 and 3.5×10^4 $CD9^-$ APCs respectively (Supplementary Fig. 7a,e). As shown in Supplementary Fig. 7b,f, $CD9^-$ APCs were transferred into D-WAT treated mice. Consistent with findings in PD mice, $CD9^-$APC recovery did not change the metabolic benefits achieved by APC depletion in DIO and *db/db* mice.

These data provide evidence that depleting $CD9^+$ APCs in obese and diabetic mice could achieve glycemic improvement. Accordingly, text in the *Results* and *Discussion* section has been revised (Page 15, lines 6-21; Page 17, lines 8-12, 17-22; Page 17, lines 2-14, 18-22; Page 18, lines 3-4; Page 19, line 16-17).

7. Figure 6. Do the mice gain weight on HFD between moths 3 and 4 after they are treated with tamoxifen or vehicle (fig 6)? The result that depletion of APCs improves metabolic phenotype goes against multiple previous data where studies suggested that differentiation of new adipocytes is metabolically beneficial compared to the increased size of mature adipocytes. Could it be that tamoxifen

injection and APC depletion has non-specific effects that are associated with metabolic phenotype.

Response:

Thanks for your comments.

As shown in supplementary Fig. 6j, after tamoxifen or vehicle treatment, body weight was not statistically different among groups of obese PD mice.

Although we could not rule out the possibility that APC depletion may interfere with adipogenesis, the influence may be minor. The reasons are: (1) Previous studies using different lineage tracing mouse models investigated the timing of APC activation and adipogenesis in response to HFD, showing that new adipocytes form in eWAT between five and eight weeks of HFD feeding[1,2]. In this study, mice were fed a HFD for more than eight weeks before APC were depleted. So, by the time of APC depletion, adipogenesis may not be affected. (2) Another study has reported that $CD9^+$ APCs are the predominant subset in the eWAT of obese mice and can hardly differentiate into mature adipocyte[3]. Consistent with previous studies, depleted APCs in this study were mainly $CD9^+$ APCs.

Nevertheless, whether APC depletion impedes adipocyte generation is an important issue to investigate. Accordingly, we have added the following text in the *Discussion* section (Page 20, lines 3-5):

"In addition, it is worth noting that APCs are critical to generate new adipocytes during the progression of obesity, whether APC depletion impedes adipocyte generation needs further investigation."

References:

1. Wang QA, Tao C, Gupta RK, Scherer PE. Tracking adipogenesis during white adipose tissue development, expansion and regeneration. Nature medicine 19, 1338-1344 (2013).

2. Jeffery, E., Church, C.D., Holtrup, B., Colman, L. & Rodeheffer, M.S. Rapid depot-specific activation of adipocyte precursor cells at the onset of obesity. Nature cell biology 17, 376-385 (2015).

3. Marcelin, G., et al. A PDGFRalpha-Mediated Switch toward CD9(high) Adipocyte Progenitors Controls Obesity-Induced Adipose Tissue Fibrosis. Cell metabolism 25, 673-685 (2017).

In addition, to further determine whether tamoxifen injection per se could alter

glycemic metabolism, PD mice and *Pdgfra*-CreERT2 mice fed a HFD for 3 months were treated with tamoxifen or vehicle (Reply Fig. 2a). As shown in the following figure, four weeks after tamoxifen or vehicle treatment in PD mice, glycemic disturbance was notably improved in tamoxifen treated mice compared to mice injecting vehicle (Reply Fig. 2b,c). However, tamoxifen treatment had no effect in HFD-fed *Pdgfra*-CreERT2 mice, as compared to PD mice injecting vehicle. These data indicate that tamoxifen injection per se could not impact glycemic metabolism.

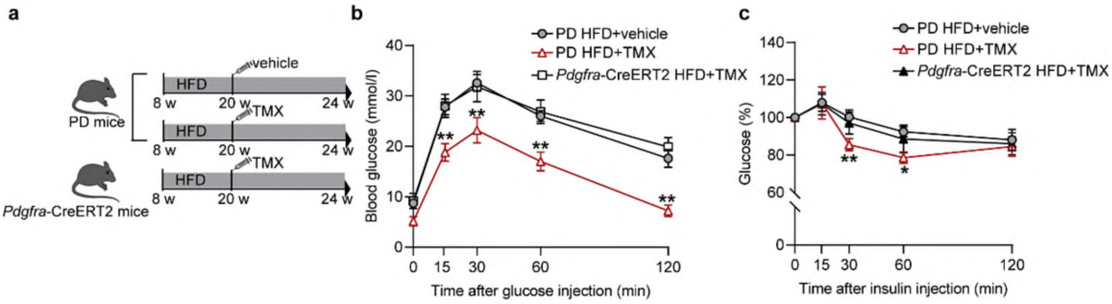

Reply Figure 2: Glycemic disturbance was not improved by tamoxifen treatment in obese *Pdgfra*-CreERT2 mice. **a**, PD mice and *Pdgfra*-CreERT2 mice were fed a HFD for 3 months and treated with tamoxifen (TMX). One month after tamoxifen treatment, GTT assay (**b**) and ITT assay (c) were conducted (n=5 per group). *, P<0.05; **, P<0.01 (One-way ANOVA followed by Tukey's multiple comparison test).

8. Could the authors show the data of weight, food intake and fat cell size in the PD mice injected with tamoxifen on HFD. In addition, how can the authors exclude that tamoxifen alone has a metabolic effect when mice are on HFD. There was no effect on chow, but metabolic conditions are changing substantially in obesity.

<u>Response:</u>

Thanks for your valuable comments.

The data of body weight, food intake and adipocyte size in APC-depleted PD mice have been added in the Supplementary Fig. 6i-m. Food intake and body weight were not statistically different among groups of obese PD mice (Supplementary Fig. 6i-k). HE staining showed that the eWAT of APC-depleted mice contained a higher proportion of large adipocytes compared to the vehicle-treated mice (Supplementary Fig. 6l,m).

These important data have been incorporated into the *Results* section in the revised manuscript (Page 15, lines 11-12, 19-21).

In addition, as we replied to the above comment, glycemic disturbance was not improved after tamoxifen injection in obese *Pdgfra*-CreERT2 mice, indicating that tamoxifen administration per se could not alter glycemic metabolism.

9. Figure 7. In the treatments of DIO and db/db mice with D-WAT, which APC population was depleted? Please present the data of depletion for different APC populations? What was food intake rate, what was the weight of these mice after the treatment.

Response:

We appreciate these helpful comments.

To address the Reviewer's question, we analyzed the effect of D-WAT on APC frequency, food intake and body weight. As illustrated in Fig. 7 and Supplementary Fig. 7, after D-WAT treatment, both $CD9^+$ APCs and $CD9^-$ APCs were reduced in DIO and *db/db* mice, whereas no significant differences were observed on food intake and body weight. These important data have been incorporated in the revised manuscript (Page 16, lines 21-22; Page 17, lines 13-14).

Bioinformatic analysis

10. Extended figure 2. Could you please include references for clusters shown in ext fig. 2c,d. The references are missing in both the text and the figure legend therefore it's difficult to understand that the authors mean. Do the authors map their clusters to cells obtained from visceral or subcutaneous clusters.

Response:

Thanks for your valuable comment.

The relevant references have been cited in the revised manuscript. The adipose tissue depot used for analysis was added in the figure legends (Fig. 1g, Supplementary Fig. 2e,f). Accordingly, these references were incorporated in the main text (Page 6, line 11).

11. CD9 cells do not correspond to any of previously identified clusters. Could the authors comment on this fact? What is the difference from the multiple other studies and the ones shown in extended figure 2? What about purity of the sample?

Response:

We appreciate this insightful question raised by the reviewer.

To better address this important issue, we performed additional analysis. In this study, to map our APC clusters with previous findings, we selected three elegant studies which have been incorporated into the main text (Page 6, line 11). Among these studies, findings from two studies (Merrick *et al.* and Schwalie *et al.*) contribute to the consensus of APC heterogeneity in mouse, while Emont *et al.* conducted single-cell sequencing on human visceral and subcutaneous adipose tissues. To better visualize our APC data compared to the published literature, we integrate our single-cell dataset with those reported by above studies and performed unsupervised cell clustering. We found that four APC subpopulations identified in our study could be finely distinguished in the integrated dataset. Moreover, *Icam1+* committed progenitor and *Dpp4+* multipotential progenitor identified by individual studies were generally preserved after integration. In agreement, as shown in Fig.1g and Supplementary Fig. 2e,f, APCs_CD55 cells resembled multipotent progenitors; APCs_ICAM1 cells expressed a gene signature similar to that of committed preadipocytes; APCs_CD142 cells tended to be adipogenesis-regulatory cells, whereas APCs_CD9 did not correspond well with a particular subpopulation.

Based on these findings, we conclude that APCs_CD9 do not correspond to any of previously identified clusters. The main text has been revised (Page 6, lines 10-14).

12. CD9-/CD55-/ICAM1- population was decreased in obese and obese T2D patients. What are these cells? Do they express CD34? PDRFRA? Can they differentiate or if this decrease only relative as CD9+ cell population is increased?

Response:

Thanks for your valuable comments.

CD34 was expressed in CD9^{-}CD55^{-}ICAM1^{-} APC subpopulations, as validated by

both single-cell analysis (Reply Fig. 3a) and flow cytometry (Reply Fig. 3b).

Furthermore, in response to standard differentiation factors *in vitro*, these cells can differentiate into adipocyte (Reply Fig. 3c).

In addition, as shown in Fig. 2e, we analyzed the relative frequency of each APC subpopulation in total APC. So, due to the increased frequency of $CD9^{+}CD55^{low}$ APCs, the frequency of $CD9^{-}CD55^{-}ICAM1^{-}$ APCs was decreased in obese and T2D patients.

Promoted by your comments, the expression level of CD34 has been added in Fig. 1d in the main text.

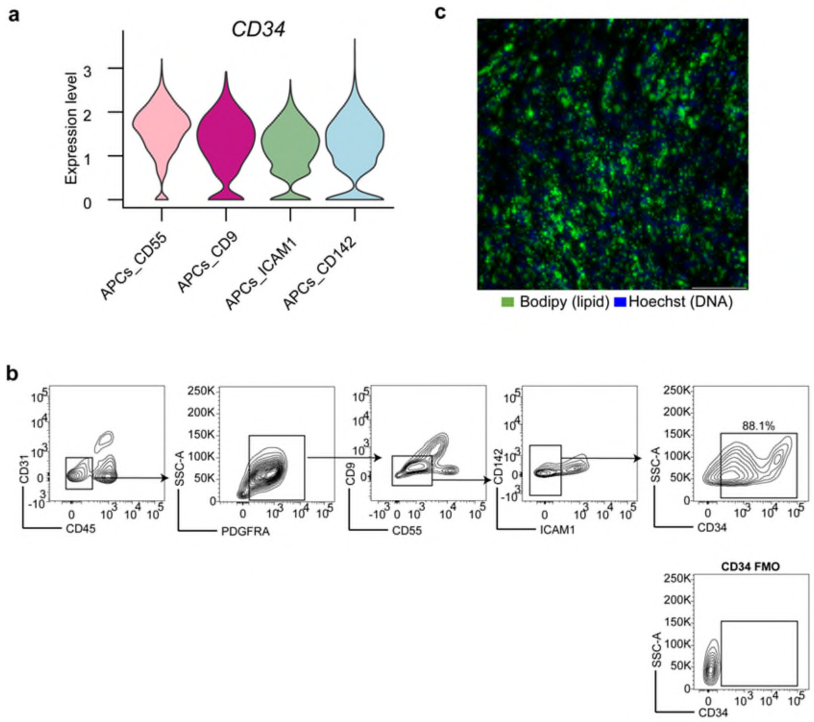

Reply Figure 3: a, Violin plot showing the expression level of CD34 in four APC subpopulations; **b**, Detection of CD34 expression in $CD9^{-}CD55^{-}ICAM1^{-}$APCs by flow cytometry; **c**, $CD9^{-}CD55^{-}ICAM1^{-}$APCs were sorted by flow cytometry and cultured *in vitro*. Twelve days after standard differentiation factors treatment, adipogenic differentiation was analyzed by BODIPY staining.

Other:

13. Could you please change colors in figure 1c to visualize contribution of each patient to the clusters.

<u>Response:</u>

As suggested, we changed colors in Fig. 1c and added a new figure depicting the contribution of each donor (Supplementary Fig. 2a). Thank you.

14. In general, figure legends needs to be expanded. In many cases, it is not clear what is shown, and n numbers are lacking. Please add n to each panel where relevant.

Response:

We thank the reviewer for the opportunity to clarify and improve our figure legends. As suggested, we have added more details in the figure legends.

15. The lightness of the microscopy pictures should be increased. It is impossible to see the staining (neither on the screen, nor in the printed version).

Response:

Thank you. We have replaced these micrographs (Fig. 3a, Fig. 3g, Fig. 6k, Fig. 7h, Fig. 7s) with high quality images in the revised manuscript.

16. The method section does not provide all the necessary details to reproduce the data shown.

Response:

Thanks for your comment.

Additional experimental details have been included in the *Methods* section to strengthen the understanding and reproducibility of this study.

<u>Reviewer #3:</u>

Major concerns:

1. In Fig. 1, the authors characterize the stromal vascular fractions derived from human visceral adipose tissues. It is important to recognize that the captured transcriptomic profiles represent a snapshot of the dynamic tissue. Throughout the manuscript, APCs_CD9 is depicted as a stable subpopulation with a distinct role. However, the slingshot trajectory analysis (Fig. 1g) positions the APCs_CD9 cluster in a transition state, situated between the APCs_CD55 progenitors and the further developed APCs_ICAM1 (committed preadipocytes) and APCs_CD142 (adipogenesis-regulatory cells). The APCs_CD9 cells have intermediate levels of marker gene expressions (Fig. 1d) and enrichment (Fig. 1h, Extended data fig. 2c,d) in between those of early progenitors and developed cells. Altogether, the profile of APCs_CD9 cells resembles a dynamic intermediate developmental cell state and not a static pathogenic cell population.

<u>Response:</u>

We thank the reviewer for raising this important question and hope to explain this question through the following points.

Firstly, single-cell sequencing is now widely used to elucidate APC heterogeneity. Although the lineage hierarchy of adipose progenitors has been recently identified, researchers now reach a consensus about the existence of functionally different APC subpopulations[2].

Secondly, due to differences in metabolic status, adipose tissue depot and species, identification of APC subpopulations varies between studies. Here, the APCs_CD9 subpopulation identified in this study is mainly ascribed to T2D patients which previous studies do not include.

Thirdly, the existence of $CD9^+$ APCs was validated by flow cytometry in our study. Using these cells from T2D patients, we conducted substantial experiments and proved its pathogenic role in disease development. Importantly, transplanted $CD9^+$ APCs could still be detected in receipt mice.

Collectively, we believe that the APCs_CD9 identified in our study is a stable

pathogenic cell population.

Reference:

1. Merrick D, et al. Identification of a mesenchymal progenitor cell hierarchy in adipose tissue. Science 364, (6438) (2019).
2. Ferrero, R., Rainer, P. & Deplancke, B. Toward a Consensus View of Mammalian Adipocyte Stem and Progenitor Cell Heterogeneity. Trends in cell biology 30, 937-950 (2020).

2. In the subsequent figures, the authors designed experiments with the assumption that CD9+CD55low is a distinct cell type. In Fig. 4 and Fig. 5, the authors focus solely on comparing CD9+CD55low cells derived from lean and obese T2D donors. It is important to consider the possibility that the distinctions observed between lean and obese donors may not be exclusive to CD9+ cells but could potentially extend to all APCs.

Response:

Thanks for your valuable comments.

We agree with the reviewer that elucidating the functional alteration of individual APC subpopulation in diabetic patients is quite essential. Indeed, altered transcriptional signatures were observed in four APC subpopulations (Fig. 2h). However, in this study, our core content and main innovations are to uncover the most pivotal subpopulation contributing to T2D development and to explore the therapy potential of targeting these cells.

As depicted in Fig. 2e-h, both number and functionality of $CD9^{+}$APC are dramatically changed in diabetic patients. Moreover, cell transplantation assays further confirmed that $CD9^{-}$APC transplantation showed no detrimental effect on glycemic homeostasis. Based on these concerns, we mainly investigated the underlying mechanisms of $CD9^{+}$ APCs in driving glycemic disturbance.

Nevertheless, we believe that this is an important question to address in our further directions. We have added the following text into the *Discussion* section in our revised manuscript (Page 23, lines 17-21):

"Besides, although the role and underlying mechanism of $CD9^{+}CD55^{low}$ APCs in diabetes are disclosed, altered transcript profiles are also observed in other APC

subpopulations. How the cellular fate changes and to what extent these alterations affect systemic metabolism need further investigation”.

3. An appropriate control group is missing in the APCs transfer experiments presented in Fig. 3, Fig. 5g-n, and Extended data Fig. 5f,g. The use of PBS as a control does not appropriately control for the impact of allogenic cell injection. Does the injection of CD9-CD55high cells also induce lipolysis?

Response:

We thank the reviewer for the valuable comments. Following the suggestions proposed by you and reviewer 2, we performed additional transfer experiments as detailed below.

1. In Fig. 3:

As suggested, we have supplemented another transplantation group using $CD9^-$ APCs from T2D patients. The results showed that mice transplanted with $CD9^+$ APCs showed more severe glucose tolerance impairment and higher fasting blood glucose compared to mice transplanted with the same number of $CD9^-$ APCs (Fig. 3d-f, m-o). Meanwhile, transplanting $CD9^-$ APCs had no effect as compared to injecting PBS.

These findings support that $CD9^+CD55^{low}$ APCs, not $CD9^-$ APCs, impair glycemic homeostasis. Based on these new data, we have revised the *Results* section (Page 9, lines 13-22; Page 10, lines 12-22).

2. In Fig. 5:

We have supplemented another transplantation group using $CD9^+CD55^{low}$ APCs from lean control subject. Compared to mice transplanted with $CD9^+CD55^{low}$ APCs from lean control subjects, mice transplanted with $CD9^+CD55^{low}$ APCs from obese T2D patients showed higher levels of serum FFA and glycerol (Fig. 5k,l). Meanwhile, transplanting $CD9^+CD55^{low}$ APCs from lean control subject had no effect as compared to injecting PBS. Accordingly, we have revised the text in the Result section (Page 13, line 20-22; Page 14, lines 1-6).

4. The authors claim that the majority of APCs in the adipose tissue of obese mice are CD9+ cells, and consequently, the metabolic effects induced by the ablation of

all APCs presented in Fig. 6 and Fig. 7 are attributed to the removal of CD9+ APCs. The assumption is based on the flow cytometry profile derived from a diet-induced obesity mouse model, which suggests that CD9+ APCs make up ~75% of the total APCs in the obese mouse model. However, this attribution is valid only if the ablation of CD9- APCs has no discernable physiological impact.

Response:

We appreciate these valuable comments, which are also proposed by reviewer 2 (Comment 6). Currently, it is technically hard to deplete one specific APC subpopulation. As we replied to reviewer 2, we reconstituted $CD9^-$ APCs in APC-depleted mice to exclude the influence of $CD9^-$APC depletion on glycemic metabolism:

1. In obese PD mice, approximately 3.5×10^4 $CD9^-$ APCs were depleted mice after tamoxifen treatment. As shown in Fig. 6a, $CD9^-$ APCs from obese PD mice were transplanted into APC-depleted mice. In agreement with our findings in Fig. 3 that $CD9^-$ APCs did not impair glycemic homeostasis, APC depletion significantly improved glycemic disturbance in obese PD mice, whereas $CD9^-$APC recovery did not alter these metabolic benefits.

2. In DIO and *db/db* mice, D-WAT treatment depleted approximately 4×10^4 and 3.5×10^4 $CD9^-$ APCs respectively (Supplementary Fig. 7a,e). As shown in Supplementary Fig. 7b,f, $CD9^-$ APCs were transferred into D-WAT treated mice. Consistent with findings in PD mice, $CD9^-$APC recovery did not change the metabolic benefits achieved by APC depletion in DIO and *db/db* mice.

These data provide evidence that depleting $CD9^+$ APCs in obese and diabetic mice could achieve glycemic improvement. Accordingly, text in the *Results* and *Discussion* section has been revised (Page 15, lines 6-21; Page 17, lines 8-12, 17-22; Page 17, lines 2-14, 18-22; Page 18, lines 3-4; Page 19, line 16-17).

5. The authors have integrated the scRNA-seq results from this study with previously published APCs datasets (page 5, line 17-18, Fig. 1h, Extended data fig. 2c,d). However, the authors did not indicate which reference studies were used.

Response:

We apologize for not citing the references used in Fig. 1h in our first draft and we have supplemented these relevant references in the revised manuscript (Page 6, line 11). Thank you.

6. On page 6, in line 9, the authors have misinterpreted the PCA plot. They assert that CD9+CD55low APCs exhibit the most distinct separation from the rest of the dataset. However, it is important to note that the first principal component, responsible for 40% of the dataset's variance, distinguishes the CD55+ population from the other populations. The second principal component, which accounts for 26% of the variance, is the one that distinguishes the CD9+ population. Therefore, it is the CD55+ population that exhibits the most obvious separation from the others.

Response:

We thank the reviewer for pointing this out. These sentences have been revised as follows:

"Principal component analysis (PCA) showed that the first principal component distinguished $CD9^{-}CD55^{high}$ APCs, while the second principal component separated $CD9^{+}CD55^{low}$ APCs from the others" (Page 7, lines 14-16).

7. In Fig. 4c, the authors have elected to only highlight proteins enriched in the control/non-T2D group. However, given that the primary focus is on examining the secretome of APCs derived from T2D patients, it would be more informative to highlight specific proteins enriched in the T2D group to be highlighted. Additionally, the protein names are presented as two clusters, raising the question of whether the heatmap is aligned correctly with the protein annotations.

Response:

We are sorry for the confusing. The misunderstanding of Fig. 4c may be due to the color and layout of two groups. To make Fig. 4c more clear, we have changed the layout and color of each group in the revised vision.

8. Different marker genes for the APCs_CD9 cells are employed in different. Genes analyzed by qPCR, as depicted in Extended Data Fig. 3b, show limited overlap with the APCs_CD9 marker genes presented in Extended Data Fig. 2a, or the CD9+CD55low APCs marker genes displayed in Fig. 2d.

Response:

We apologize for the confusion. In Supplementary Fig. 3b of our first draft, genes analyzed by qPCR were the up-regulated genes in obese and T2D patients, which were included in module 3 and 4 (Fig. 2h). To make the article more clear, we added a heatmap depicting genes of module 3 and 4 as a new Supplementary Fig. 3c in the revised manuscript.

Minor concerns:

9. The gene SFRP4 (secreted frizzled related protein 4) is incorrectly spelled as SFBP4 on page 4, line 17.

Response:

This mistake has been corrected in the revised manuscript (Page 5, line 18).

10. The authors should offer a rationale for selecting the APCs_CD55 cluster as the root state of the Slingshot pseudo-time analysis.

Response:

We appreciate your helpful suggestion.

As described above, APC_CD55 cluster mapped well with APC_CD26 cluster, which has been identified as multipotent progenitor cells. Based on that, APC_CD55 cluster was selected as the root state of pseudo-time analysis. As suggested, these important data have been incorporated in the revised manuscript (Page 6, line 22; Page 7, line 1).

11. On page 9, line 19, it should read “little” instead of “litter.”

Response:

This mistake has been corrected in the revised manuscript (Page 11, line 10).

12. The authors should clarify in the figure legends the meaning of the blue data points in the volcano plot presented in Fig. 5a.

Response:

The volcano plot shows the differentially secreted proteins in culture medium of $CD9^{+}CD55^{low}$ APCs from obese T2D patients, as compared to lean control subjects. The blue data points indicated the decreased proteins, whereas the red data points indicated the increased proteins. Accordingly, both Fig. 5a and its figure legend have been revised.

REVIEWER COMMENTS

Reviewer #2 (Remarks to the Author):

The authors have made an extensive revision, adding additional control groups and supporting experiments, which has increased the quality of the manuscript. Clarity of the manuscript is also much improved.
- Although the specific depletion of CD9+CD55low population is still questionable, the authors added additional reconstitution experiments and explained challenges in specific removal of CD9+CD55 population.
- Another remaining concern is a fact that CD9+CD55low population could be found only by this current study, but not by multiple earlier studies. Although authors now state this fact, the reason for this is not clear an explanation is not given. Why was this population unique for this study? However in general, questions that were raised are addressed in an acceptable way and the manuscript is substantially improved.

Reviewer #3 (Remarks to the Author):

In the revised manuscript, the authors repeated the initially presented studies, incorporating additional controls. I commend the authors for composing the revision within a short time frame, which involved recruiting human visceral adipose tissue donors, conducting in vivo transplantation experiments, subjecting the animals to 2-3 months high fat diets, and performing functional characterizations. The inclusion of additional controls and the revised figure legends has substantially enhanced the interpretability the results. The authors maintain that the claims presented in the initial manuscript remain unchanged. However, as presented, major concerns raised in the initial review were not addressed:

1. While I fully agree with the authors regarding the existence of functionally distinct APC subpopulations and CD9+ APCs, I invited the authors to discuss on the developmental state of the APCs_CD9 population in the context of trajectory analysis presented in Fig. 1h. If this population is indeed stable, why is the cluster located in the intermediate point of the two trajectories rather than the endpoint of a separate trajectory?

2. In Fig. 1 to 3, the authors identified CD9+CD55low APCs as a population of interest with pathogenic implications. In Fig. 4 and 5, the authors presented data indicating functional differences between CD9+CD55low APCs derived from obese T2D patients and lean controls. Contrary to these findings, the authors generically defined CD9+CD55low APCs as a pathogenic APC subpopulation in the conclusion. This contradicts the presented results:
- CD9+CD55low APCs are readily detected in all lean individuals profiled (Fig. 1e, Fig. 2e), and there are no indications these individuals will ever become obese or develop diabetes.
- CD9+CD55low APCs from lean individuals display no pathogenicity in the transplantation experiments presented in Fig. 4g and in Fig. 5.

Do the authors imply that obesity and T2D status cause CD9+CD55low APCs to become pathogenic? If that's the case, please revise the manuscript to emphasize that only the diabetic CD9+CD55low APCs are pathogenic. The current results, as presented, do not support CD9+CD55low APCs as a generic pathogenic APC subpopulation.

3. In Fig. 6 and 7, the authors indiscriminately ablated all APC populations in the adipose tissue of obese mice and observed metabolic phenotypic changes. Reviewer #2 and I both raised the concern that these phenotypes may be due to non-specific effects of ablation. In the revised study, the authors attempted to reconstitute the CD9- APCs to mimic a CD9+CD55low specific ablation. However, the authors did not demonstrate that the injected CD9-APCs were functionally reconstituted. Furthermore, the authors did not address the initial concern that the metabolic phenotype is due to the non-specific effect of cell ablations (tamoxifen injection, cell death, etc.). A possible negative control could be to inject unsorted APCs following APCs ablation and demonstrate a full rescue of the metabolic phenotype.

Reviewer #4 (Remarks to the Author):

"Single-cell analysis reveals a subpopulation of adipose progenitor cell that impairs glucose homeostasis" by Hongdong Wang and colleagues is an important and impactful study based on a large body of clinical and preclinical data. They identify a CD9+CD55low population of APCs that is pro-diabetic in adoptive transfer experiments. Their function is being linked to the production of specific bioactive proteins that upregulate adipocyte lipolysis. Importantly, depletion of these cells in mice either genetically or with an experimental drug D-WAT improved obesity-related glycemic disturbance.

The concerns and suggestions from the previous reviews have been largely addressed. New data and Figures are compelling. I have minor suggestions to be incorporated for the revision.

Because the depletion reconstitution experiments provide crucial evidence for the role of CD9+CD55low cells, methods used for depletion should be specified in the abstract.
There is no information that I could find on D-WAT. What is D-WAT exactly? Was this peptide made from L or D amino-acids as in the original citation? Where and how was it synthesized? Purity? QC?

A follow-up paper by the same group shows that long-term repercussion of APC depletion with D-WAT is adipocyte hypertrophy, fibrosis, and metabolic dysfunction. https://doi.org/10.1038/s42255-020-00320-4
See Extended Data Fig. 9
https://www.nature.com/articles/s42255-020-00320-4/figures/17
This should be cited and discussed.

For clarity, it would be better to define APCs_CD9 = CD9+CD55low as they are first described and then stick to one name throughout. Same for the other populations.

A recent report showing that aging increases the expression of CD9 and other fibrogenic genes in

fibroblastic ASPCs and blocks their differentiation into beige adipocytes should be cited. DOI: 10.1101/2023.03.20.533514

The title wording should be "Single-cell analysis reveals a subpopulation of adipose progenitor cells that impairs glucose homeostasis"

REVIEWER COMMENTS

Reviewer #2 (Remarks to the Author):

The authors have made an extensive revision, adding additional control groups and supporting experiments, which has increased the quality of the manuscript. Clarity of the manuscript is also much improved.

1. Although the specific depletion of CD9+CD55low population is still questionable, the authors added additional reconstitution experiments and explained challenges in specific removal of CD9+CD55 population.

Response:

We appreciate your constructive comments for improving the quality of this work. Thank you.

2. Another remaining concern is a fact that CD9+CD55low population could be found only by this current study, but not by multiple earlier studies. Although authors now state this fact, the reason for this is not clear an explanation is not given. Why was this population unique for this study? However in general, questions that were raised are addressed in an acceptable way and the manuscript is substantially improved.

Response:

Thanks for your comment.

Due to differences in metabolic status, adipose tissue depot and species, identification of APC subpopulations varies between studies [1-3]. Here, the $CD9^{+}CD55^{low}$ APC subpopulation identified in this study is mainly ascribed to T2D patients which previous studies do not include.

We have added this information into the *Discussion* section (Page 19, lines 4-7).

References:

1. Merrick D, et al. Identification of a mesenchymal progenitor cell hierarchy in adipose tissue. Science 364,6438 (2019).
2. Schwalie PC, et al. A stromal cell population that inhibits adipogenesis in mammalian fat depots. Nature 559, 103-108 (2018).
3. Emont MP, et al. A single-cell atlas of human and mouse white adipose tissue. Nature 603, 926-933 (2022).

Reviewer #3 (Remarks to the Author):

In the revised manuscript, the authors repeated the initially presented studies, incorporating additional controls. I commend the authors for composing the revision within a short time frame, which involved recruiting human visceral adipose tissue donors, conducting in vivo transplantation experiments, subjecting the animals to 2-3 months high fat diets, and performing functional characterizations. The inclusion of additional controls and the revised figure legends has substantially enhanced the interpretability the results. The authors maintain that the claims presented in the initial manuscript remain unchanged. However, as presented, major concerns raised in the initial review were not addressed:

1. While I fully agree with the authors regarding the existence of functionally distinct APC subpopulations and CD9+ APCs, I invited the authors to discuss on the developmental state of the APCs_CD9 population in the context of trajectory analysis presented in Fig. 1h. If this population is indeed stable, why is the cluster located in the intermediate point of the two trajectories rather than the endpoint of a separate trajectory?

Response:

Thanks for your comment.

In this study, Monocle3 was employed to generate the UMAP graph-based visualization of trajectory, in which *CD9*$^+$ APCs located in the intermediate position. Prompted by your comment, we performed more analysis using another classic R package “Monocle2” to further predict the cellular trajectories of these cell clusters. As shown in the following figure, *CD55*$^+$ APCs can transition through the first and second branch points to become *CD9*$^+$ APCs. Some *CD55*$^+$ APCs continue transit through the third branch point to form the cluster of *ICAM1*$^+$ or *CD142*$^+$ APCs. We have incorporated these new data and the following text into our revised manuscript (Figure 1i, Page 7, lines 3-6; Page 30, lines 10-22; Page 31, lines 1-3):

“Another trajectory analysis inferred that *CD55*$^+$ APCs can transition through the first and second branch points to become *CD9*$^+$ APCs. Some *CD55*$^+$ APCs continue

transit through the third branch point to form the cluster of *ICAM1*$^+$ or *CD142*$^+$ APCs".

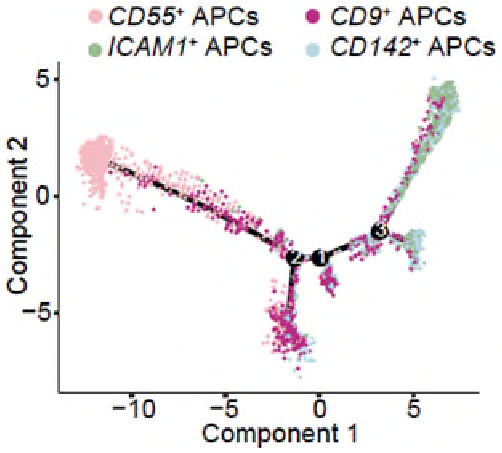

Figure legend: Cell trajectory analysis of APCs by Monocle 2.

2. In Fig. 1 to 3, the authors identified CD9+CD55low APCs as a population of interest with pathogenic implications. In Fig. 4 and 5, the authors presented data indicating functional differences between CD9+CD55low APCs derived from obese T2D patients and lean controls. Contrary to these findings, the authors generically defined CD9+CD55low APCs as a pathogenic APC subpopulation in the conclusion. This contradicts the presented results:

CD9+CD55low APCs are readily detected in all lean individuals profiled (Fig. 1e, Fig. 2e), and there are no indications these individuals will ever become obese or develop diabetes.

CD9+CD55low APCs from lean individuals display no pathogenicity in the transplantation experiments presented in Fig. 4g and in Fig. 5.

Do the authors imply that obesity and T2D status cause CD9+CD55low APCs to become pathogenic? If that's the case, please revise the manuscript to emphasize that only the diabetic CD9+CD55low APCs are pathogenic. The current results, as presented, do not support CD9+CD55low APCs as a generic pathogenic APC subpopulation.

Response:

Thanks for pointing this out. We agree with you that CD9$^+$CD55^{low} APCs from diabetic patients are dysfunctional and contribute to glycemic disturbance, whereas

$CD9^+CD55^{low}$ APCs from lean individuals are not pathogenic. As suggested, we have revised the manuscript (Page 18, lines 17,20; Page 19, line 8; Page 24, lines 13,14,16).

3. In Fig. 6 and 7, the authors indiscriminately ablated all APC populations in the adipose tissue of obese mice and observed metabolic phenotypic changes. Reviewer #2 and I both raised the concern that these phenotypes may be due to non-specific effects of ablation. In the revised study, the authors attempted to reconstitute the CD9- APCs to mimic a CD9+CD55low specific ablation. However, the authors did not demonstrate that the injected CD9-APCs were functionally reconstituted. Furthermore, the authors did not address the initial concern that the metabolic phenotype is due to the non-specific effect of cell ablations (tamoxifen injection, cell death, etc.). A possible negative control could be to inject unsorted APCs following APCs ablation and demonstrate a full rescue of the metabolic phenotype.

Response:

Thank you very much. Your constructive suggestions are very helpful for us to improve the quality of this work.

A previous study has reported that $CD9^-$ APCs of obese mice retain adipogenic capacity[1]. Although it is technically hard to trace the newly differentiated adipocytes in $CD9^-$APC transplanted mice, we isolated the reconstituted $CD9^-$ APCs and analyzed their adipogenic potential *in vitro*. As depicted in the following figure (**a**), one week after cell transplantation, reconstituted $CD9^-$ APCs were isolated by flow cytometry and cultured in 96 well plates, with freshly isolated $CD9^-$ APCs from vehicle treated obese PD mice cultured as a control group. The adipogenic index was comparable in two groups, indicating that adipogenic capacity of transplanted $CD9^-$ APCs was unimpaired (Figure **b**,**c**).

Meanwhile, promoted by your suggestion, unsorted APCs were transplanted into APC depleted diabetic mice to further ascertain the specific effect of APC depletion on glycemic regulation. One week after tamoxifen injection in obese PD mice, total APCs from untreated obese PD mice were sorted and transplanted into APC depleted

mice. Three weeks thereafter, improved glucose tolerance was observed in APC-depleted mice, but not in mice received total APCs transplantation (Figure **d**,**e**). Meanwhile, reconstituted APC was confirmed by flow cytometry (Figure **f**,**g**). As for *db/db* mice, three days after D-WAT treatment, total APCs from untreated *db/db* mice were sorted and transplanted into D-WAT treated mice. Consistent with findings in PD mice, total APC transplantation reversed the metabolic benefits achieved by APC depletion in *db/db* mice (Figure **h**,**i**). Also, flow cytometry confirmed the reconstitution of total APC (Figure **j**,**k**). Collectively, these data provide evidence that metabolic improvement after APC depletion is not due to non-specific effect of cell ablations.

Accordingly, we have incorporated these new data into the revised manuscript (Supplementary Fig.8, Page 19, lines 21-22; Page 20, lines 1-4).

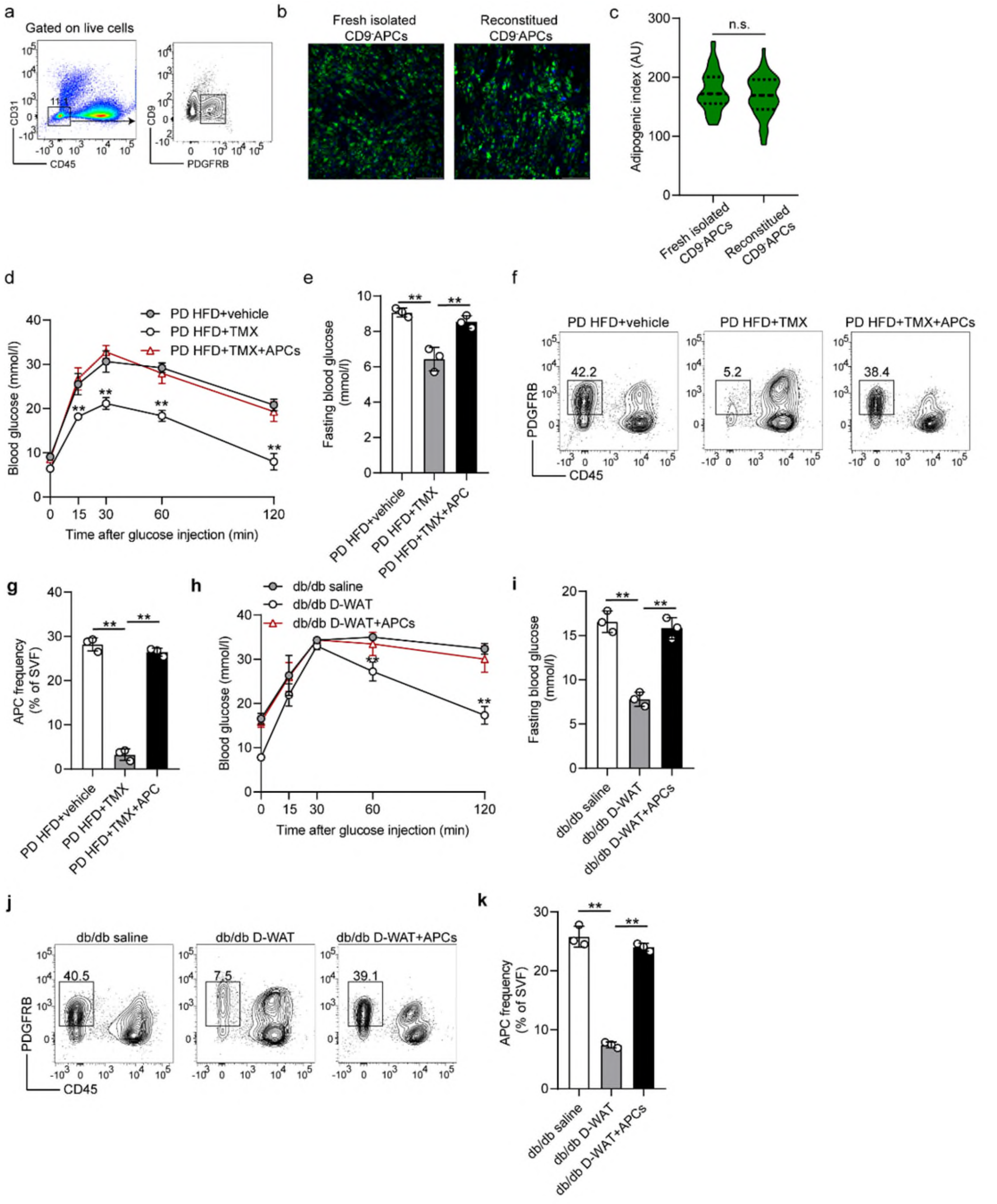

Figure legend: About 3.5×10^4 $CD9^-$ APCs isolated from untreated obese PD mice were transplanted into the eWAT of APC-depleted PD mice. One week after transplantation, reconstituted $CD9^-$ APCs were isolated by flow cytometry and cultured in 96 well plates. Meanwhile, freshly isolated $CD9^-$ APCs from vehicle treated obese PD mice were used as a control group. After exposure to standard differentiation factors *in vitro* for 8 days, the adipogenic capacity was analyzed (**a-c**). **a** Soring strategy of mouse $CD9^-$ APCs. **b** Staining of adipocytes (with Bodipy lipid stain). **c** Quantification of adipogenesis. For **d-g**, one week after tamoxifen injection in obese PD mice, total APCs from untreated obese PD mice were sorted and transplanted into APC depleted mice (n=3 per group). Three weeks thereafter, glucose tolerance test was conducted (**d**) and fasting blood glucose levels were determined (**e**). After that, the eWAT of mice were collected to detect APC by flow cytometry (**f**,**g**). For **h-k**, three

days after D-WAT treatment, total APCs from untreated *db/db* mice were sorted and transplanted into D-WAT treated *db/db* mice (n=3 per group). Three weeks later, glucose tolerance test was conducted (**h**) and fasting blood glucose levels were determined (**i**). Meanwhile, APCs in mouse eWAT were detected by flow cytometry (**j**) and quantified (**k**). For statistical analysis, **c**, Two-tailed unpaired Student's *t* test, n.s., no significant; **d**,**e**,**g-i**,**k** One-way ANOVA was used followed by Tukey's multiple comparison test, ** P<0.01.

Reference:

1. Marcelin G, et al. A PDGFRalpha-Mediated Switch toward CD9(high) Adipocyte Progenitors Controls Obesity-Induced Adipose Tissue Fibrosis. Cell metabolism 25, 673-685 (2017).

Reviewer #4 (Remarks to the Author):

"Single-cell analysis reveals a subpopulation of adipose progenitor cell that impairs glucose homeostasis" by Hongdong Wang and colleagues is an important and impactful study based on a large body of clinical and preclinical data. They identify a CD9+CD55low population of APCs that is pro-diabetic in adoptive transfer experiments. Their function is being linked to the production of specific bioactive proteins that upregulate adipocyte lipolysis. Importantly, depletion of these cells in mice either genetically or with an experimental drug D-WAT improved obesity-related glycemic disturbance. The concerns and suggestions from the previous reviews have been largely addressed. New data and Figures are compelling. I have minor suggestions to be incorporated for the revision.

1. Because the depletion reconstitution experiments provide crucial evidence for the role of CD9+CD55low cells, methods used for depletion should be specified in the abstract.

Response:

Thanks for your comment. The abstract has been revised.

2. There is no information that I could find on D-WAT. What is D-WAT exactly? Was this peptide made from L or D amino-acids as in the original citation? Where and how was it synthesized? Purity? QC?

Response:

More details about D-WAT peptide have been added in the *Methods* section (Page 26, lines 12-14). Thank you.

3. A follow-up paper by the same group shows that long-term repercussion of APC depletion with D-WAT is adipocyte hypertrophy, fibrosis, and metabolic dysfunction. https://doi.org/10.1038/s42255-020-00320-4

See Extended Data Fig. 9

https://www.nature.com/articles/s42255-020-00320-4/figures/17

This should be cited and discussed.

Response:

As suggested, we have cited this study and added the following sentences in the *Discussion* section (Page 20, lines 14-17):

"Moreover, depleting APCs at different stage of obesity may cause diverse consequences. Depleting APCs in subcutaneous adipose tissue through subcutaneously injection of D-WAT before the development of obesity could induce beige adipocyte differentiation, while long-term APC depletion causes adipocyte hypertrophy and tissue fibrosis."

4. For clarity, it would be better to define APCs_CD9 = CD9+CD55low as they are first described and then stick to one name throughout. Same for the other populations.

Response:

As suggested, the names of four APC clusters have been revised to keep consistent throughout the manuscript.

5. A recent report showing that aging increases the expression of CD9 and other fibrogenic genes in fibroblastic ASPCs and blocks their differentiation into beige adipocytes should be cited. DOI: 10.1101/2023.03.20.533514

Response:

As suggested, we have incorporated this study into the *Discussion* section as follows (Page 23, lines 19-21):

“In addition, in inguinal adipose tissue of aged mice, impaired *de novo* differentiation of APC into beige adipocyte in response to cold exposure was related to upregulation of CD9 and other fibrotic genes.”

6. The title wording should be "Single-cell analysis reveals a subpopulation of adipose progenitor cells that impairs glucose homeostasis"

Response:

The title has been revised. Thank you for pointing this out.

REVIEWERS' COMMENTS

Reviewer #2 (Remarks to the Author):

The authors addressed raised questions. I have no further comments.

Reviewer #3 (Remarks to the Author):

In the latest revision, the authors have addressed most concerns raised in the initial and follow-up comments. The added monocle analysis and transplantation studies present further support for the claim that CD9+ APCs are a distinct and detrimental cell population. It would be insightful for the author to provide some discussions or speculations on why CD9+ APCs from lean individuals display no pathogenicity in the transplantation experiments, as presented in Fig. 4g and in Fig. 5.

Reviewer #4 (Remarks to the Author):

There are no more concerns.

REVIEWER COMMENTS

Reviewer #2 (Remarks to the Author):

The authors addressed raised questions. I have no further comments.

Response:

Thank you very much for providing constructive comments.

Reviewer #3 (Remarks to the Author):

In the latest revision, the authors have addressed most concerns raised in the initial and follow-up comments. The added monocle analysis and transplantation studies present further support for the claim that CD9+ APCs are a distinct and detrimental cell population. It would be insightful for the author to provide some discussions or speculations on why CD9+ APCs from lean individuals display no pathogenicity in the transplantation experiments, as presented in Fig. 4g and in Fig. 5.

Response:

We appreciate your time and expertise for improving the quality of our work. As suggested, the following sentences have been incorporated into the revised manuscript (Page 22, lines 7-11).

"It is also worth noting that, adipose tissue of lean subject harbors $CD9^{+}CD55^{low}$ APCs and transient transplantation of $CD9^{+}CD55^{low}$ APCs from lean subjects displays no significant pathogenicity. These findings indicate that $CD9^{+}CD55^{low}$ APCs are not genetically pathogenic but become detrimental during the progression of obesity."

Reviewer #4 (Remarks to the Author):

There are no more concerns.

Response:

Thank you very much for raising all the helpful comments.
